# Probabilistic classification of gene-by-treatment interactions on molecular count phenotypes

**DOI:** 10.1371/journal.pgen.1011561

**Published:** 2025-04-09

**Authors:** Yuriko Harigaya, Nana Matoba, Brandon D Le, Jordan M Valone, Jason L Stein, Michael I Love, William Valdar

**Affiliations:** 1 Department of Genetics, University of North Carolina at Chapel Hill, Chapel Hill, North Carolina, United States of America; 2 UNC Neuroscience Center, University of North Carolina at Chapel Hill, Chapel Hill, North Carolina, United States of America; 3 Carolina Institute for Developmental Disabilities, Carrboro, North Carolina, United States of America; 4 Department of Biostatistics, University of North Carolina at Chapel Hill, Chapel Hill, North Carolina, United States of America; Emory University School of Medicine, UNITED STATES OFAMERICA

## Abstract

Genetic variation can modulate response to treatment (G×T) or environmental stimuli (G×E), both of which can be highly consequential in biomedicine. An effective approach to identifying G×T signals and gaining insight into molecular mechanisms is mapping quantitative trait loci (QTL) of molecular count phenotypes, such as gene expression and chromatin accessibility, under multiple treatment conditions, which is termed response molecular QTL mapping. Although standard approaches evaluate the interaction between genetics and treatment conditions, they do not distinguish between meaningful interpretations such as whether a genetic effect is observed only in the treated condition or whether a genetic effect is observed always but accentuated in the treated condition. To address this gap, we have developed a downstream method for classifying response molecular QTLs into subclasses with meaningful genetic interpretations. Our method uses Bayesian model selection and assigns posterior probabilities to different types of G×T interactions for a given feature-SNP pair. We compare linear and nonlinear regression of log ⁡ -scale counts, noting that the latter accounts for an expected biological relationship between the genotype and the molecular count phenotype. Through simulation and application to existing datasets of molecular response QTLs, we show that our method provides an intuitive and well-powered framework to report and interpret G×T interactions. We provide a software package, ClassifyGxT [1].

## Author summary

Responses to treatment, such as drug, therapeutic intervention, and infection, can vary across individuals at least in part due to their genetic backgrounds. Such modulation of treatment response by genotypes, or equivalently, modulation of genotype effects by treatment is called gene-by-treatment (G×T) or gene-by-environment (G×E ) interactions. Understanding G×T or G×E interactions can potentially improve strategies for prevention and treatment of diseases, for example by selecting treatments for which a patient is most likely to respond given their genetic information, or enhanced screening for individuals most susceptible to environmental exposures. Most existing methods for G×T or GxE analysis focus on detecting interactions, not classifying them. For example, they do not distinguish between the genotype effect being present only upon treatment vs the genotype effect always being present but accentuated by treatment. Herein, we propose a statistical method to classify GxT or GxE types and supply suitable software. Our method provides an intuitive way to interpret GxT or GxE interactions.

## Introduction

Gene-by-treatment (G×T ) interactions describe associations between genotype and phenotype that are modulated by treatment or, equivalently, phenotypic responses to treatment that are modulated by genotype. Understanding G×T interactions is crucial for interpreting disease-associated genetic variants and, eventually, for clinical decision-making. These phenomena may also be called gene-by-environment (G×E) interactions in a broader context [2].

An effective approach to G×T discovery is quantitative trait loci (QTL) mapping, which examines statistical associations between genotype and phenotype. For G×T discovery, there exist at least three types of methods, all of which use phenotype data in control and treated conditions. In the first approach, which we call the “stratified” approach, the association between genotype and phenotype is examined separately in each of the conditions. Then, genetic variants involved in G×T interactions are identified as those that exhibit significant association with the genotype only in the treated condition [3,4]. In the second approach, which requires paired data and which we call the “delta” approach, tests association of the genotype with an outcome variable defined by the phenotypic difference between paired control and treated conditions [3,5–7]. In the third approach, which we term the “interaction” approach but is also called “response QTL mapping”, phenotype data in the control and treated conditions is jointly modeled using a linear model including the genotype, treatment, and G×T interaction terms [8–12]. Multiple studies undertaking these approaches have been effective in identifying a large number of G×T interactions, which have, in turn, led to valuable biological insights about genetic control of cellular and tissue responses to treatment.

**Fig 1:**
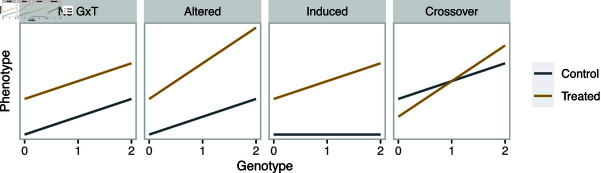
Illustration of the presence and absence of G×T interactions. Schematics of hypothetical linear regression where the phenotype is regressed on the genotype, coded as 0, 1, and 2, separately in the control (gray) and treated (brown) conditions. Shown are examples of lack of G×T interaction (no-G×T), the altered genotype effect pattern, where the genotype effect is present in both conditions but to different strengths, the induced genotype effect pattern, where the genotype effect emerges only upon treatment, and the crossover interaction, where the treatment has the opposite effect depending on the genotype.

Despite their effectiveness in G×T detection, however, the aforementioned approaches are not well suited to classifying the type of G×T interaction that is present. Specifically, the way in which genetics differ under alternative treatments can fall into starkly different classes. To illustrate this point, we consider graphical representation of G×T interactions through a linear regression framework, where the phenotype is regressed on the genotype at the site of a single nucleotide polymorphism (SNP) in the control and treated conditions separately (Fig 1). Without loss of generality, we will refer to genetic variants as SNPs. Whereas parallel regression lines indicate the absence of G×T interactions (“no-G×T ”), non-parallel regression lines represent the presence thereof. In some cases, association between the phenotype and genotype is present in both conditions but to different extents, which we call the “altered” genotype effect pattern. In other cases, association between the phenotype and genotype is present only in the treated condition, which we call the “induced” genotype effect pattern, where the SNP would be discovered as a putative *cis*-regulatory variant only upon treatment. Furthermore, G×T interaction can result in a situation where the treatment has the opposite effects (i.e., effects with different signs) depending on the genotype, which is often called a “crossover” interaction effect [13]. Formally distinguishing between these different types of G×T interactions can lead to deeper insights into mechanisms by which SNPs may affect the phenotype. For example, the induced pattern may occur when the transcription factor whose binding is modulated by the genetic variant is only activated after treatment, or when the transcriptional response necessitates co-factors that are only active after treatment [10,14]. The crossover interaction may imply a mechanism where the SNP modulates a regulatory element, which, in turn, mediates transcriptional activation in one condition and repression in the other condition to alter the basal level of gene expression. In the current practice, a typical mapping procedure returns a list of feature-SNP pairs with significant associations from *p*-value based hypothesis testing and does not provide probabilistic classification of these different types of G×T interactions without requiring *ad hoc* post-processing (see Results for details). This limitation can potentially hamper meaningful interpretation and prioritization for downstream analysis. Some studies have undertaken probabilistic approaches to detecting G×T or G×E interactions, though these proposed methods focused specifically on paired designs [15,16].

Another potential limitation of the current practice in G×T analysis is specifically relevant to molecular QTL mapping, where the phenotype is molecular count data, such as gene expression or chromatin accessibility. In a typical procedure for molecular QTL mapping, the molecular count data is transformed, commonly using the log ⁡ function, and a linear relationship is assumed between the genotype and the transformed molecular phenotype. Previous studies, however, have shown that, in most cases, the linearity holds in the original count scale, not in the transformed scale, a phenomenon known as *allelic additivity*, and that assuming linearity on the transformed scale can result in the genotype effect being estimated inaccurately [17–19]. Such inaccuracy can potentially lead to a pitfall in G×T analysis, which infers the *difference* in genotype effects between treatment conditions, even though it may not substantially impact single-condition molecular QTL mapping, which infers the *presence* of genotype effects. To our knowledge, the issue of allelic additivity and modeling of molecular counts for response QTL classification has not been addressed or evaluated in the published literature.

In this study, we developed a method for probabilistic classification of types of G×T interactions on molecular count phenotypes using Bayesian model selection (BMS). Our method takes a set of SNPs identified by standard mapping procedures, such as the “stratified”, “delta”, and “interaction” approaches, as input and generates posterior probabilities for candidate models representing different types of G×T interactions. Within this framework, we examined three modeling approaches: 1) applying a linear model to the log ⁡ transformation of the molecular phenotype (log ⁡ -LM); 2) applying a linear model to the rank-based inverse Normal transformation (RINT) [20] of the molecular phenotype (RINT-LM); and 3) applying a nonlinear model to the log ⁡ transformation of the molecular phenotype that explicitly accounts for allelic additivity (log ⁡ -NL). In our simulation experiments, in which we generated data according to the known nonlinear relationship between the genotype and transformed molecular phenotype, we observed that nonlinear regression (log ⁡ -NL) exhibited moderately but consistently higher accuracy than linear regression (log ⁡ -LM and RINT-LM). We also observed that empirical Bayes approaches to elicit priors can affect the posterior probabilities of the correct model as well as those of the incorrect models. We then illustrate the utility of our method through reanalysis of previously published gene expression and chromatin accessibility data in human primary neural progenitor cells (hNPCs) as examples.

## Results

### A Bayesian model selection (BMS) framework for classifyingG×Tinteractions with molecular count phenotypes

#### Overview of the framework.

We focus on the effects of a treatment on the association between a genotype and molecular phenotype, such as gene expression and chromatin accessibility measured by RNA-sequencing (RNA-seq) [21] and assay of transposase-accessible chromatin sequencing (ATAC-seq) [22] techniques. In what follows, we refer to the unit being measured for a given molecular phenotype as a feature: for gene expression, the feature is a gene; for chromatin accessibility, the feature is an accessible chromatin region, often called a candidate *cis*-regulatory element (cCRE).

We aim to provide a principled approach to interpreting and prioritizing feature-SNP pairs that have been identified by standard methods, such as the “stratified”, “delta”, and “interaction” approaches (Introduction), for further experimental or bioinformatic interrogation. The types of feature-SNP pairs include expression QTLs (eQTLs) and chromatin accessibility QTLs (caQTLs). The framework takes the genotype and molecular phenotype data across subjects in control and treated conditions for a pre-selected set of feature-SNP pairs as input, and outputs posterior probabilities of eight possible models, representing whether the regression coefficients for the genotype, treatment, and G×T interaction terms are non-zero. We note that the well-recognized challenges of specifying an appropriate false discovery rate in detecting G×T or G×E interactions [12] are not directly relevant to our method, which aims to *classify*, rather than *detect*, G×T interactions for feature-SNP pairs that have already been selected.

**Fig 2:**
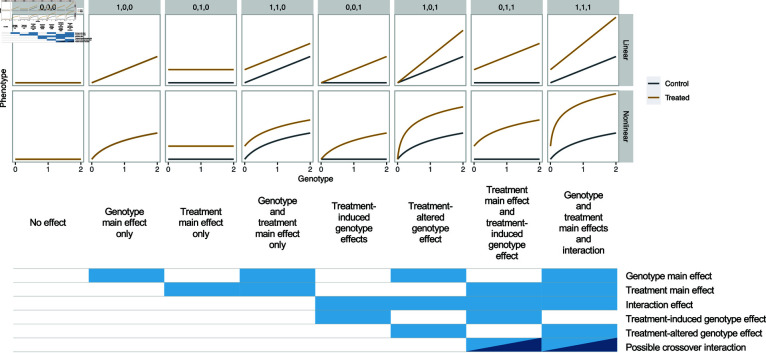
Illustration of types of G×T interactions. Schematics of hypothetical linear regression where the phenotype is regressed on the genotype, coded as 0, 1, and 2, separately in the control (gray) and treated (brown) conditions. Shown on the top are the m vectors. Top: Linear models. Bottom: Nonlinear models. Shown below the schematics is the taxonomy of G×T interactions. (1,1,1). See text for details.

We refer to the eight model categories using a vector of indicator variables $m=(m_{g},m_{t},m_{g\times t})$, where *m_g_*, *m_t_*, and *m_g×t_* are each 1 or 0 and denote the inclusion and exclusion of the genotype, treatment, and G×T interaction terms in the model, respectively (Fig 2). In the simplest form using linear regression, the model is


$$\begin{array}{ccc} y_{i}=\beta_{0}+m_{g}\beta_{g}g_{i}+m_{t}\beta_{t}t_{i}+m_{g\times t}\beta_{g\times t}g_{i}t_{i}+\varepsilon_{i}, & & \end{array}$$ (1)

where *i* indexes samples, *y_i_* is the log ⁡ -transformed molecular count data, *g_i_* is the genotype coded as {0,1,2} or the imputation-based allelic dosage $g_{i}\in [0,2]$, *t_i_* is an indicator variable for a treatment, $\beta ={\left(\beta_{0},\beta_{g},\beta_{t},\beta_{g\times t}\right)}^{T}$ denotes the regression coefficients, and $\varepsilon_{i}∼N(0,\sigma^{2})$ is the residual error with error variance *σ*^2^. Each of the eight models can be obtained by substituting elements of the corresponding **m** vector for *m_g_*, *m_t_*, and *m_g×t_* in Eq (1). For example, **m**=(0,0,0) corresponds to the intercept only model,


$$y_{i}=\beta_{0}+\varepsilon_{i}\;,$$

**m**=(1,1,0) corresponds to


$$y_{i}=\beta_{0}+\beta_{g}g_{i}+\beta_{t}t_{i}+\varepsilon_{i}\;,$$

and **m**=(1,1,1) corresponds to


$$y_{i}=\beta_{0}+\beta_{g}g_{i}+\beta_{t}t_{i}+\beta_{g\times t}g_{i}t_{i}+\varepsilon_{i}\;.$$

Thus, models corresponding to (0,0,1), (1,0,1), (0,1,1), and (1,1,1) include the G×T interaction term, whereas those corresponding to (0,0,0), (1,0,0), (0,1,0), and (1,1,0) do not. These model categories also apply to nonlinear regression that we propose in this work (Fig 2). It is useful to consider the biological meaning of the eight model categories through the taxonomy and corresponding nomenclature in Fig 2 and Table 1. Nonetheless, we will primarily refer to the models using the **m** vectors in the rest of the manuscript for simplicity.

We are primarily motivated by analyzing datasets generated in *in-vitro* cell systems with and without treatment. In this type of data, samples from both control and treatment conditions are available for the same donor; there may be replicate samples per combination of donor and condition; or replication may be consistently or sporadically absent. To account for a possible error correlation structure within a group of samples derived from the same donor, our framework can include effects of donors and/or genetic relatedness (kinship) between them as a random effect (SI Text).

**Table 1:** Types of G×T interactions.

m vector	Taxonomic nomenclature	Aggregate category
(0,0,0)	No effect	No G×T
(1,0,0)	Genotype main effect only	No G×T
(0,1,0)	Treatment main effect only	No G×T
(1,1,0)	Genotype and treatment main effects only	No G×T
(0,0,1)	Treatment-induced genotype effect	Induced
(1,0,1)	Treatment-altered genotype effect	Altered
(0,1,1)	Treatment main effect and treatment-induced genotype effect	Induced
(1,1,1)	Genotype and treatment main effects and interaction	Altered

#### A BMS approach to classifying G×T.

We propose a BMS approach, which consists of four steps, as in Bayesian approaches more generally. First, we specify models that consist of likelihood and prior probabilities (Methods). Second, we compute the posterior probability. Third, we summarize the posterior probability of interest, which is the probability corresponding to different types of G×T interactions. Fourth, we optionally make a decision based on individual or aggregated posterior probabilities. For this decision, we select the model with the highest posterior probability (i.e., the posterior mode).

We note that three of our models, (1,0,1), (0,1,1), and (0,0,1), contain the interaction term without one or both of the main effect terms, which could be seen as counter to the principle of marginality [23], which forbids such formulations, and in tension with the related principle of heredity [24], which gives them diminished or zero prior probabilities. Our reading of these principles, however, is that they primarily apply to model selection that proceeds sequentially (i.e., fitting main effects first, then interactions, not the other way around) or large-scale model search (where ruling out models *a priori* is computationally advantageous), and to modeled data where a zero main effect has no meaningful interpretation. By contrast, our model selection is not sequential, since we compare the full repertoire of possible models simultaneously; we are examining a small space of models; and the models with zero main effects are tied to interpretations that are specific and meaningful in this biological context.

In BMS, it is necessary to specify prior probabilities for the models that are being compared, which we call the model prior, and prior distributions for the parameters in the models, $\beta =(\beta_{g},\beta_{t},\beta_{g\times t})$, which we call the effect prior (Methods). For the model prior, we assign an equal probability of one-eighth to all models. We consider it a reasonable choice since our method is designed to classify G×T interactions for pre-selected feature-SNP pairs rather than to detect interactions among all candidate pairs that are dominated by the null model. In our specification of the effect prior (Methods), the hyperparameters $\phi =(\phi_{g},\phi_{t},\phi_{g\times t})$ control the effects relative to the residual error standard deviation, which correspond to the signal-to-noise ratios. The default specification that has been used in previous studies [25,26] is *ϕ* = ( 1 , 1 , 1 ) , which assumes roughly equal effect sizes that are similar to the residual error standard deviation. Here, we optimize the hyperparameters using empirical Bayes (see later sections).

The framework also provides three types of post-processing, which correspond to the third step of BMS described above. First, we compute posterior probabilities of aggregated categories, which can be more relevant than each of the eight models in practical settings. This is straightforward since the eight models are mutually exclusive and the posterior probabilities of aggregated categories can be obtained by summing posterior probabilities of the corresponding models. For example, the posterior probability of models where the G×T interaction is non-zero is computed as the sum of the posterior probabilities of (0,0,1), (1,0,1), (0,1,1), and (1,1,1); we refer to this aggregated category as “interaction” and denote it by (∗,∗,1). It is also possible and useful to compute the probability of the induced genotype effect, corresponding to (0,∗,1), where the association between the genotype and phenotype emerges only upon treatment, as well as the probability of the altered genotype effect, corresponding to (1,∗,1), where the genotype-phenotype association exists in the absence of treatment but is the strength of the association is altered upon treatment (Fig 1). Other possible aggregated categories of interest include that of the “restricted” treatment effect, corresponding to (∗,0,1), where the treatment only affects individuals with genotype levels 1 and 2, as well as the “varying” treatment effect thereof, corresponding to (∗,1,1), where the treatment affects all individuals but to different extents, while we do not specifically consider these further in this manuscript.

The second type of post-processing computes the posterior probability of a crossover interaction, where the treatment has the opposite effects (i.e., effects with different signs) depending on the genotype (Fig 1, Methods). The third classifies feature-SNP pairs into 27 groups that correspond to $m\in {\left\{-1,0,1\right\}}^{3}$, taking into account the signs of the regression coefficients (Methods).

#### Computational approaches.

The computation of posterior probabilities is analytically tractable with the linear model in Eq (1) but not with the nonlinear model or in the presence of random effects. Therefore, we used two approximations: 1) Markov Chain Monte Carlo (MCMC) followed by bridge sampling, and 2) maximum a posteriori (MAP) estimation followed by Laplace approximation (Methods and SI Text). In general, the MCMC approach is more accurate but computationally less efficient than the MAP approach (SI Text). Based on our analysis where we assessed the accuracy and efficiency of the two approaches (SI Text), we propose to use the latter to optimize the hyperparameters and then use the former for posterior inference.

#### Non-BMS approaches to classifyingG×T.

In this section, we discuss the advantage of BMS over alternative approaches for classification. We first consider an alternative approach that has been used in practice and that consists of multiple steps, combining aspects of both stratified and response QTL mapping. It proceeds as follows: 1) (molecular) QTL mapping is performed in the control and treated conditions to identify feature-SNP pairs that are significant after multiple testing correction; 2) feature-SNP pairs that are significant under treatment but not control conditions are designated as being significant for G×T; 3) for each identified G×T feature-SNP pair, further hypothesis testing is used to distinguish between, e.g., (0,0,1) and (0,1,1), by testing for the significance of the treatment main effect in an interaction model. Although conceptually simple, this approach yields a single, high variance estimate that lacks any kind of uncertainty quantification—that is, it provides no information about the expected rate of false positive classifications. Moreover, classification based on hypothesis testing is inherently unsatisfactory due to the asymmetry between the null and alternative hypotheses — that is, it only rejects or fails to reject and cannot select the null model. Other possible approaches include significance-guided model selection, such as forward selection and backward elimination [27], which involve sequential hypothesis testing. However, the same caveat applies to these hypothesis testing-based approaches.

An approach more akin to BMS is to fit all the eight models and choose a model using a model selection criterion, such as Akaike’s An Information Criterion (AIC) [28] or the Bayesian information criterion (BIC) [29]. Though this approach is entirely feasible for our purpose due to the small number of models being compared, it also lacks uncertainty quantification, at least without additional enclosing procedures such as resampling [30–32]. For example, consider a scenario where BMS assigns posterior probability of 1.0 to (0,1,1) and another scenario where posterior probabilities 0.4 and 0.6 are assigned to (0,0,1) and (0,1,1), respectively. In either scenario, criterion-based selection would choose (0, 1, 1) with no indication of the fact that the latter scenario implies substantially higher uncertainty.

#### Modeling the relationship between molecular phenotype and genotype:transformations and allelic additivity.

In the current practice of molecular QTL mapping, molecular count phenotypes are subject to a variance-stabilizing transformation, such as the log ⁡ transformation, and the effects of genotype are modeled linearly on the transformed scale. As described earlier, however, the allelic additivity assumption [18,19] posits that the effect of genotype is linear on the original count scale, such that it is preferable to model the log-transformed count *y_i_* using a nonlinear model as


$$y_{i}=\log(\mu_{g_{i}})+\varepsilon_{i}\;,$$

where *μ_gi_* is defined for $g_{i}\in \{AA,AB,BB\}$ as


$$\mu_{AA}=\exp(\beta_{0})\;,\;\mu_{BB}=\exp(\beta_{0}+2\beta_{g})\;,\;\text{and}\;\mu_{AB}=\frac{1}{2}\mu_{AA}+\frac{1}{2}\mu_{BB}\;,$$

for a SNP with the major allele A and the minor allele B.

**Fig 3:**
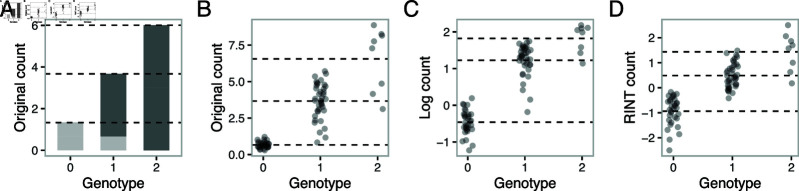
The assumption of allelic additivity. A. Barplots illustrating that allelic additivity results in the linear relationship between the phenotype and the genotype. The dark and pale gray boxes represent the hypothetical molecular count signals from the two alleles. B. An example of molecular count data on the original scale. Circles represent samples; dashed horizontal lines correspond to the mean expression values conditional on the genotype. C. The same as molecular count data in B but on the log ⁡ scale. Although the variance is more homogeneous than in B, the phenotype with respect to the genotype is no longer linear. This type of pattern is captured by log ⁡-NL in each condition. D. The same as the molecular count data in B but with RINT transformation.

To illustrate the allelic additivity assumption, we consider the effect of a genetic variant in a *cis*-regulatory region on the expression of a target gene. A common assumption is that gene regulatory feedback mechanisms are rare and that gene expression from different alleles is independent. This leads to the assumption that, in a diploid cell, the total gene expression count is the sum of gene expression count from the two alleles and that the gene expression count is linear with respect to the genotype (Fig 3A). Fig 3B shows an example of previously published gene eQTL data in hNPCs in the original count scale, which is consistent with this idea. However, after variance-stabilizing transformation to achieve homoscedastic error, the mean expression values are no longer linear with respect to the genotype (Fig 3C). Similarly, RINT-transformation does not preserve the linear relationship unless there are approximately equal numbers of major and minor allele homozygous donors (Fig 3D). With the RINT transformation, moreover, adjustment for covariates, such as sex, cannot be handled properly, and large effects can be greatly underestimated. A similar argument also applies to molecular count data other than gene expression. Regardless, in practice, linearity is commonly assumed, which can lead to inaccurate inference. Although some previous studies have addressed this issue by accounting for the nonlinearity between transformed molecular count data and genotype [18,19] for eQTL mapping under a single condition, to our knowledge, this type of nonlinear model has not been used in response QTL mapping. As we shall see, failing to account for this nonlinear relationship can lead to reduced accuracy, and reduced separation of posterior probabilities of correct models from incorrect ones.

To account for allelic additivity, we adapted the nonlinear model for single-condition molecular QTL mapping described above to G×T analysis (Eq (11)). The method, which we term log ⁡ -NL, explicitly models the nonlinear (NL) relationship between the genotype and the log ⁡-transformed molecular count phenotype. We call the commonly used methods, which assume a linear relationship between the genotype and the log ⁡- or RINT-transformed phenotype, as log ⁡-LM and RINT-LM, respectively, referring to the types of transformation. Throughout the manuscript, we consider comparisons between these three approaches.

### Assessing the allelic additivity in hNPCs with growth stimulation

Previous studies have analyzed gene expression data from genetically diverse donors in the postmortem human tissues and found little evidence to suggest deviations from the allelic additivity [18,19]. To assess whether the conclusion also holds in *in-vitro* cell systems, we reanalyzed previously published eQTL data in hNPCs, a system in which numerous associations between regulatory features and SNPs have been identified [11,33–35]. In this analysis, we focused on 3073 feature-SNP pairs with significant G×T pairs (Methods) and fit the log ⁡-transformed data to the nonlinear and linear models by maximum likelihood estimation (MLE) for each condition separately (Methods). For comparison, we also fit a larger, more flexible model with a categorical genotype variable consisting of three levels, which can describe both nonlinear and linear relationships.

The analysis led to the following observations. First, across the feature-SNP pairs, the maximized likelihood values are higher for the nonlinear model than for the linear model or similar between the models (S1 Fig). Since the number of fitted parameters is identical, this suggests that the nonlinear allelic additivity model is more adequate than the linear model. Second, the values are similar between the nonlinear and larger models, suggesting that the nonlinear model sufficiently captures the complexity of the data with fewer parameters (S1 Fig). Third, the values are lower for the linear model than for the larger model, or they are similar between the models (S1 Fig). Overall, the results show little evidence for deviations from the allelic additivity, consistent with previous studies [18,19].

We next examined whether the allelic additivity holds for chromatin accessibility, focused on 83488 feature-SNP pairs with significant G×T pairs (Methods), and observed essentially the same trends as for the gene expression data (S1 Fig). To our knowledge, this is the first evidence to suggest that chromatin accessibility signals with respect to the genotype are linear in the original count scale in the majority of cases.

### Simulation: classifying G×Tinteractions using BMS

We evaluated our G×T classification procedure using simulation. Since we observed little evidence of deviations from allelic additivity in experimental data, we assumed this mechanism to simulate data from the eight models specified in Eq (11) (Methods). The true parameter values were set according to the nonlinear regression results for the response eQTLs in hNPCs (Methods). The data comprised one observation per condition for 80 donors (total sample size *n*=160). We simulated four scenarios, by generating data with and without a donor random effect, and performing analyses with or without a donor random effect term in the model. Specifically, these correspond to a setting without random effect (scenario 1), that with donor random effect in model fitting but not in data generation (scenario 2), that with donor random effect in data generation but not in model fitting (scenario 3), and that with donor effect in both data generation and model fitting (scenario 4). We used MAP estimation followed by Laplace approximation to elicit the effect prior in a data-driven manner. For posterior inference, we additionally used MCMC followed by bridge sampling (Methods and SI Text). We consistently observed convergence of MCMC (S1 Table). In addition to assessing each of the eight models, we computed posterior probabilities of the aggregated model categories no-G×T, induced, and altered. Within the BMS framework, we compared the performance of the three modeling approaches, log ⁡-NL, log ⁡-LM, and RINT-LM as follows. First, we examined the distributions of posterior probabilities of the correct models (i.e., the data-generating model) as well as those of the incorrect models. Second, we stratified the data based on the correct model and the posterior mode model and inspected the distribution of posterior probabilities in each stratum. Third, we constructed receiver operating characteristics (ROC) curves assessing classification error at different posterior probability thresholds. Fourth, we assessed calibration of the procedure, that is, whether the posterior probabilities inferred for each model match the empirical frequencies with which those models were used to generate data [36]. Fifth, we investigated the quality of effect estimation by computing the root mean squared error (RMSE) across feature-SNP pairs.

**Fig4:**
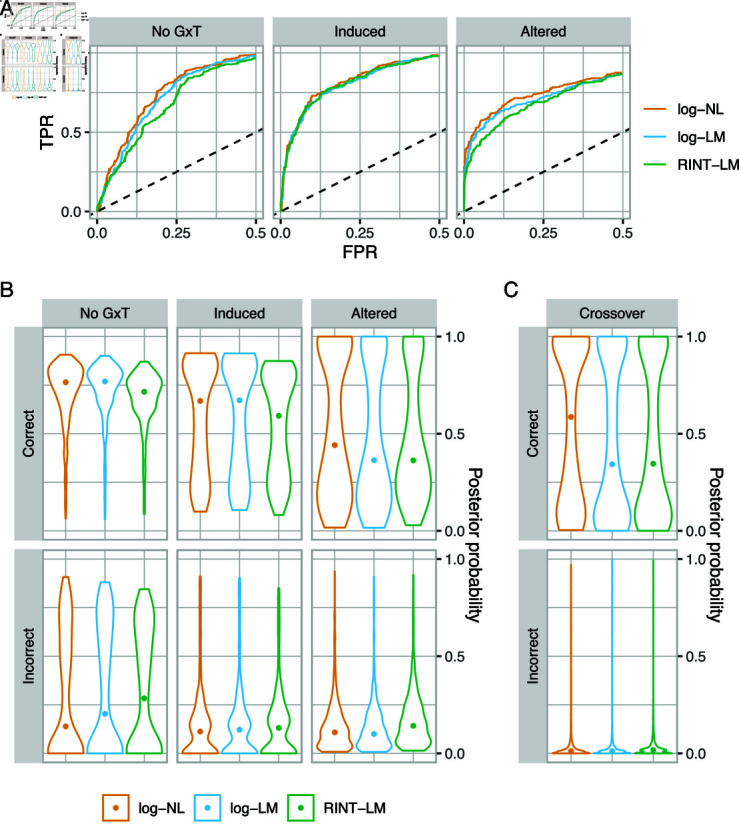
BMS on simulated data. A. Partial ROC curves comparing log ⁡ -NL, log ⁡ -LM, and RINT-LM for the no-G×T, induced, and altered model categories. B. Violin plots showing distributions of the posterior probability of the correct (top) and incorrect (bottom) models for log ⁡ -NL, log ⁡ -LM, and RINT-LM. Shown in the top of each panel is the model category. C. The same as A but for crossover interactions.

The results were similar across the scenarios and led to the following points. Overall, the three modeling approaches show comparable performance as assessed by ROC curves (S2 Fig) as well as by calibration (S3 Fig) [37]. In the stratified analysis, the posterior mode models matched the correct models for a large fraction of instances across all methods (S4 Fig) [37]. Nonetheless, a superior performance of log ⁡-NL was evident in partial ROC curves for no-G×T and altered categories (Figs 4A and S5) [37]. There was also a consistent tendency for log ⁡-NL to outperform log ⁡-LM and RINT-LM, as assessed by the distribution of the posterior probability of the correct (i.e., data-generating) model and incorrect (i.e., not data-generating) models thereof (Figs 4B and S6) [37]. Specifically, for the aggregated model categories, no-G×T, induced, and altered, log ⁡-NL gave higher median posterior probabilities of the correct model as well as lower median posterior probabilities for the incorrect models than other approaches. This was also the case for the individual model categories except for (1,0,0) and (1,0,0), where the median posterior probability was higher for log ⁡ -LM than for log ⁡ -NL (S7 Fig) [37]. The posterior probability of the correct models may be low for a fraction of the simulation instances, due to the level of error variance chosen for the simulation; still, there exist clear distributional differences between the correct and incorrect models. The performance of BMS for selecting correct models can rather be evaluated by the calibration and ROC curves (Figs 4A, S3, and S2) [37]. Consistent with the superior performance of log ⁡ -NL, we observed that log ⁡ -LM gave strongly biased estimates for genotype and G×T interaction effects and that RINT-LM did so for all three effects (Table 2, S8 Fig) [37]. These trends did not significantly differ across computational strategies. However, with SNPs for which the minor allele homozygote was absent, we observed occasional, moderate inaccuracies as well as rare failures of MAP estimation followed by Laplace approximation (SI Text and S9 Fig) [37]. Hence, we propose to restrict the use of MAP estimation followed by Laplace approximation to hyperparameter optimization and to perform posterior inference using MCMC followed by bridge sampling in practice (SI Text and S10 Fig).

**Table 2:** RMSE of effect estimates across methods.

	Genotype			Treatment			G×T		
**Scenario**	**log ⁡ -NL**	**log ⁡ -LM**	**RINT-LM**	**log ⁡ -NL**	**log ⁡ -LM**	**RINT-LM**	**log ⁡ -NL**	**log ⁡ -LM**	**RINT-LM**
1	0.228	0.474	0.517	0.172	0.224	0.428	0.250	0.333	0.372
2	0.234	0.455	0.496	0.190	0.215	0.393	0.253	0.336	0.368
3	0.267	0.475	0.535	0.212	0.263	0.450	0.261	0.333	0.378
4	0.252	0.447	0.497	0.183	0.210	0.376	0.256	0.334	0.366

Root mean squared errors (RMSE) of genotype, treatment, and G×T interaction effects relative to residual standard errors across 800 feature-SNP pairs. The estimates were obtained by Bayesian model averaging (Methods). Scenarios 1, 2, 3, and 4 represent a setting without random effect, that with donor random effect in model fitting but not in data generation, that with donor random effect in data generation but not in model fitting, and that with donor effect in both data generation and model fitting, respectively.

#### Sensitivity of the BMS to the effect prior specification.

Prior specification of the model parameters can affect the accuracy of BMS. To assess the sensitivity of our BMS to the effect prior specification, we performed BMS with varying values of the hyperparameters, $\phi =(\phi_{g},\phi_{t},\phi_{g\times t})$. Specifically, we set them to half and twice of the optimal values, which are restrictive and permissive, respectively. We then assessed the performance of BMS by ROC curves and the distribution of posterior probabilities. The results showed that the perturbation had little impact on the overall performance of BMS as assessed by ROC curves (S11 and S12 Figs). We, however, observed moderate but consistent impacts of the effect prior specification on the posterior probabilities. With the restrictive effect priors, more complex models were favored. By contrast, with the permissive effect priors, simpler models were favored (S13 Fig) [37].

#### Identifying crossover interactions through post-processing

In practice, one situation of interest is where the G×T interaction results in the treatment effect acting in the opposite direction depending on the genotype (crossover interaction) (Introduction). Probabilistic characterization of such an event is only possible and straightforward with BMS using MCMC. To identify feature-SNP pairs of this type, we performed post-processing to compute the posterior probability of crossover interaction for the 8000 datasets generated from the nonlinear allelic additivity model (Methods). In this post-processing method, we approximated the posterior probability of a crossover interaction by the fraction of MCMC samples that satisfy the necessary and sufficient condition, which is that the differences in the mean values at *g*=0 and *g*=2 have the opposite signs. The ground truth was obtained by examining the simulated effects of the homozygous genotype (*g*=0 or *g*=2) under alternate treatments and prior to the addition of noise. The posterior probabilities of crossover interaction were then obtained from log ⁡ -NL, log ⁡ -LM, and RINT-LM models for both truly crossover and non-crossover cases. We observed that log ⁡ -NL gave higher median posterior probability than log ⁡ -LM and RINT-LM in cases where there was a true crossover interaction, and lower median posterior probability when there was not (Fig 4C).

Overall, the results from the simulation analysis suggest strong performance of our BMS framework as well as advantages of log ⁡ -NL over log ⁡ -LM and RINT-LM.

### Classifying G×Tinteractions for response eQTLs in hNPCs with growth stimulation

As an example application of our BMS framework, we computed posterior probabilities for the types of G×T interactions for a set of response eQTLs identified in hNPCs by Matoba *et al.* [11]. In particular, we focused on 98 response eQTLs on autosomes with the CHIR treatment (Methods). The response eQTLs represent pairs of genes and index SNPs for which the G×T interaction term was significantly non-zero based on hypothesis testing. CHIR, also known as CHIR99021, is an activator of the canonical Wnt pathway, which has been implicated in proliferation of hNPCs, cortical patterning, and complex brain traits [38–43]. As in the simulation experiments, we compared the three modeling approaches (log ⁡ -NL, log ⁡ -LM, RINT-LM), but using mixed effect models to account for the genetic relatedness between the donors (Methods). We used MAP estimation followed by Laplace approximation for hyperparameter optimization, and MCMC followed by bridge sampling for posterior inference (Methods and SI Text). We consistently observed convergence of MCMC (S2 Table).

**Fig 5:**
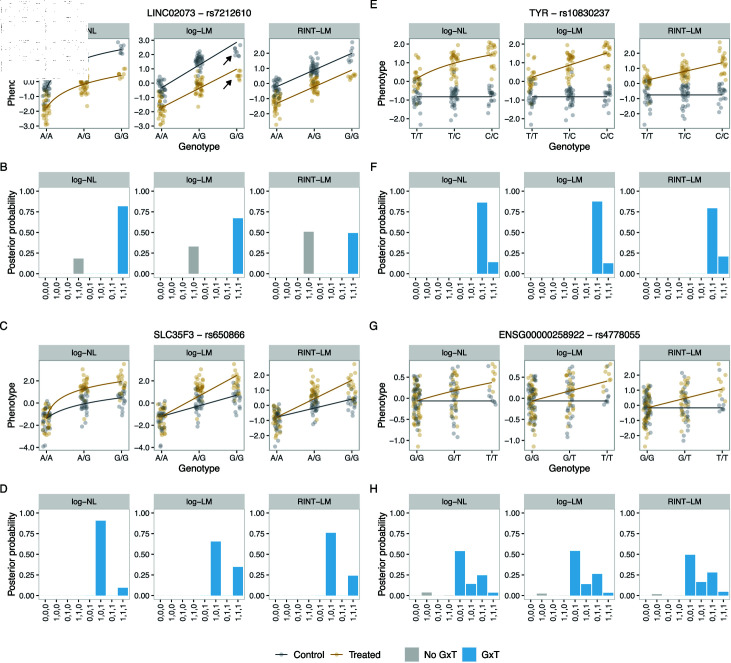
Representative BMS results for the response eQTL in hNPCs. The upper panels show fitted functional relationships between the genotype and the transformed molecular count phenotype based on the posterior mode model. The circles represent the data. The gray and brown colors represent the control and treated conditions, respectively. The arrows indicate deviations from regression lines (A, C, E, G). The lower panels show posterior probability of the different types of G×T interactions. The gray and blue colors represent the probability of the no-G×T and “interaction” categories, respectively. (B, D, F, H). Within each subfigure, the left, middle, and right panels show results from log ⁡ -NL, log ⁡ -LM, and RINT-LM, respectively.

Overall, the analysis led to the following two points. First, the BMS results were largely concordant with the previous response QTL mapping results at the level of calling significant interactions of genotype and treatment (S14 Fig). Specifically, of the 98 previously identified response eQTLs, G×T models achieved the highest posterior probabilities in 94 eQTL analyzed by log ⁡ -NL, with 95 for log ⁡ -LM, and 92 for RINT-LM. The small discrepancy is likely due to the difference in the data preprocessing (Methods). Second, the three approaches gave comparable but not identical classification and inference. Specifically, for the 90 out of 98 feature-SNP pairs, the posterior mode models were identical between approaches. Examples of feature-SNP pairs with varying degrees of concordance from the three modeling approaches are shown in Fig 5. For the long intergenic non-protein coding RNA LINC02073 and its neighboring SNP, rs7212610, the highest posterior probability was given to (1,1,1) by both log ⁡ -NL and log ⁡ -LM, but not by RINT-LM (Fig 5A and 5B). Furthermore, log ⁡ -NL captures the reduction of the genotype effect size upon treatment with higher certainty than log ⁡ -LM, likely due to its ability to account for the nonlinear relationship between the genotype and phenotype. The result is consistent with the idea that accuracy in effect size estimation can have a nonnegligible impact on the detection of G×T interactions. For the *SLC35F3* gene encoding a thiamine transporter and its neighboring SNP, rs650866, all three modeling approaches assigned the highest and second highest posterior probabilities to (1,0,1) and (1,1,1), respectively. However, log ⁡ -NL gave larger certainty on the posterior mode model than log ⁡ -LM and RINT-LM did. A possible explanation for this observation is that the inadequate linear constraint of log ⁡ -LM as well as the nonparametric transformation in the RINT-LM approach led to an increased probability of an incorrect inference of the treatment effect for the major allele homozygote (Fig 5C and 5D). Indeed, the mean phenotype values for the major allele homozygote were -1.45 and -1.39 with the standard errors of 0.97 and 0.87 for the control and treated conditions, respectively, which did not differ significantly (*P*=0.68 from a paired *t*-test).

We also computed aggregated probabilities of interest. For 16 and 75 feature-SNP pairs, the results from the three modeling approaches agreed in assigning the highest posterior probability to the induced and altered genotype effect patterns, respectively, among the three aggregated categories (no-G×T, induced, and altered). The TYR gene encoding a tyrosinase and its neighboring SNP, rs10830237, as well as the novel long non-coding RNA gene ENSG00000258922 and its neighboring SNP, rs4778055, provide examples of the induced category (Fig 5E, 5F, 5G and 5H).

Overall, the analysis illustrates the utility of our framework in that it provides interpretable posterior probabilities rather than hard classifications without uncertainty quantification. It also suggests potential advantages in employing the log ⁡-NL approach for response QTL. We note that similar results were obtained using mixed effect models to account for possible error correlation structures between samples derived from the same donor (donor random effect) rather than polygenic (kinship) effect (Methods and S15 Fig).

#### Identifying crossover interactions and accounting for signs of effects in the hNPCdata.

**Fig 6:**
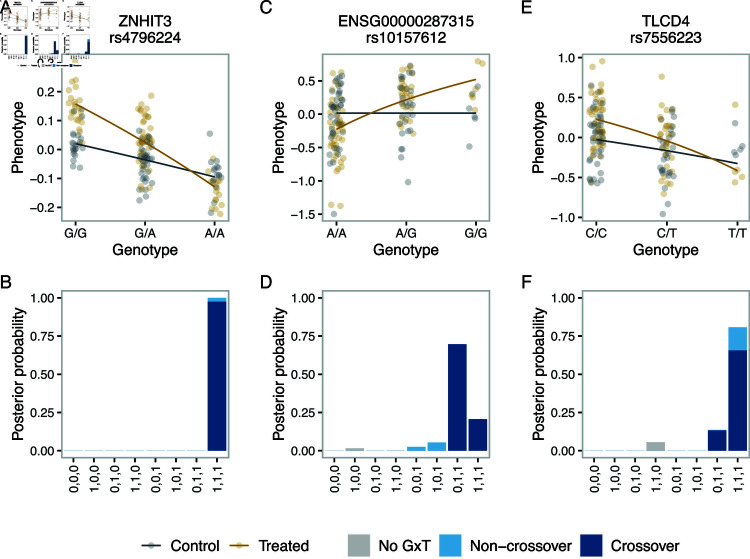
Examples of response eQTLs with the crossover interaction in hNPCs. The upper panels show fitted functional relationships between the genotype and the transformed molecular count phenotype based on the posterior mode model obtained by BMS with log ⁡ -NL. The circles represent the data. The gray and brown colors represent the control and treated conditions, respectively (A, C, E). The lower panels show posterior probability of the different types of G×T interactions obtained by BMS with log ⁡-NL. The gray color represents the probability of the no-G×T category. The pale and dark blue colors represent the probability of the “non-crossover” and crossover interactions, respectively (B, D, F).

As discussed earlier, a crossover interaction, where the treatment has the opposite effect depending on the genotype, can imply a gene regulatory mechanism where a regulatory element mediates transcription activation and repression depending on the treatment condition (Introduction). To identify gene-SNP pairs with this type of association, we performed post-processing of the log ⁡-NL results to compute the posterior probability of crossover interaction for the previously identified 98 response eQTLs (Methods). Examples with high posterior probability of crossover interactions are shown in Fig 6. Interestingly, for the *ZNHIT3* gene encoding the zinc finger HIT domain-containing protein 3, which is known to be defective in a severe encephalopathy, and a neighboring SNP, rs4796224, the posterior probability of crossover interaction was close to one (Fig 6A and 6B). In this case, the model category (1,1,1) solely received non-zero probability. By contrast, for the ENSG00000287315 gene encoding a novel antisense transcript and a neighboring SNP, rs10157612, the model categories (1,1,0) and (1,1,1) both received non-zero posterior probability (Fig 6C and 6D). For the *TLCD4* gene encoding the TLC domain-containing protein 4 and a neighboring SNP, rs7556223, log ⁡ -NL, log ⁡ -LM, the posterior probability of a crossover interaction was lower than for other examples, representing the uncertainty (Fig 6E and 6F). We emphasize that this utility is uniquely provided by BMS and presents its additional advantage.

In practice, the distinction between positive and negative effects can be crucial for the interpretation and prioritization of response molecular QTLs. Post-processing of the output from our BMS framework can generate posterior probability of 27 categories that correspond to $m\in {\left\{-1,0,1\right\}}^{3}$, accounting for the sign of the effect sizes. The results of such analysis are summarized as heatmaps in S14 Fig.

### Classifying G×Tinteractions with respect to chromatin accessibility

To illustrate the usability of our framework on a molecular phenotype other than gene expression, we reanalyzed 1775 autosomal response caQTLs identified previously with the CHIR treatment [11]. Analogously to the analysis of the response eQTLs, this analysis led to the following observations. First, the BMS results are reasonably concordant with the response caQTL results at the level of calling significant interactions of genotype and treatment (S16 Fig). Specifically, for 1588, 1588, and 1523 of the 1775 feature-SNP pairs, the log ⁡ -NL, log ⁡ -LM, and RINT-LM approaches, respectively, assigned the highest posterior probability to one of the G×T interaction models. The three approaches gave comparable but not identical classification and inference. For the 1516 feature-SNP pars, the posterior mode models were identical between them. However, we observed a number of examples where the posterior probability patterns substantially differed between the approaches yet gave the same highest posterior probability model (S17 Fig). Among the three aggregated categories, no-G×T, induced, and altered, for 538 and 848 feature-SNP pairs, the results from the three modeling approaches agreed in assigning the highest posterior probability to the induced and altered effect patterns, respectively. An example of the induced effect pattern is shown in S17 Fig. We also observed a number of feature-SNP pairs with the crossover interaction. Examples are shown in S18 Fig. The classification results accounting for the sign of the effect size are summarized as heatmaps in S16 Fig.

### Computational performance

Computational cost is a key issue in applying Bayesian methods, particularly those involving MCMC, to large genomics data. To systematically evaluate the computational performance of our framework, we measured the runtime and memory usage of BMS using MCMC followed by bridge sampling, or MAP estimation followed by Laplace approximation, on a Linux-based system. In this series of analyses, we focused on five randomly selected feature-SNP pairs from each set of simulated and experimental data and repeated measurements 10 times per feature-SNP pair. In all instances, the memory usage was less than 0.6 gigabytes (S3 and S4 Tables). The mean values of the 10 runtime measurements ranged from 107 to 306 seconds for MCMC followed by bridge sampling (S3 Table) and from 0.15 to 0.50 seconds for MAP estimation followed by Laplace approximation (S4 Table). Thus, MAP estimation followed by Laplace approximation was approximately one thousand times faster than MCMC followed by bridge sampling. Roughly put, even with MCMC, hundreds and thousands of feature-SNP pairs can be analyzed within a day if they are processed as parallel jobs on a standard computer cluster. For example, hyperparameter optimization for 1775 caQTLs over 1728 grid points using MAP estimation followed by Laplace approximation with all three modeling approaches required approximately 700 minutes; subsequent posterior inference for the same 1775 caQTLs using the hyperparameter-optimized MCMC followed by bridge sampling required approximately 100 minutes.

## Discussion

We have developed a method to help interpret and prioritize response molecular QTLs (i.e., pre-selected feature-SNP pairs with significant G×T interactions). The method uses BMS to assign posterior probability to different types of G×T interactions. Within this framework, we compared three different modeling approaches, log ⁡ -NL, log ⁡ -LM, and log ⁡ -RINT. The first approach, log ⁡ -NL, assumes that molecular signals are additive with respect to allelic counts and explicitly models the nonlinear relationship between the genotype and the molecular counts after the log ⁡ transformation. The log ⁡-NL approach is justified based on previous studies as well as our analysis examining the adequacy of the model using experimental data [17–19], whereas the other approaches are commonly used. In our simulation experiments with realistic effect and sample sizes for *in-vitro* cell systems, log ⁡ -NL outperformed other approaches moderately but consistently. Our analysis of previously published experimental data illustrates the utility of our framework in extracting practically relevant information from response molecular QTL mapping data. Individual inspections of the experimental data and fitted models revealed cases where the failure of linear regression of log ⁡ -transformed counts in capturing the data characteristics led to posterior probability patterns that differ from those obtained by nonlinear regression. These observations collectively suggest the benefit of nonlinear regression over the commonly used linear regression.

Our method is analytically intractable and uses MCMC followed by bridge sampling as well as MAP estimation followed by Laplace approximation. Our measurements of runtime suggest that the latter is three orders of magnitude faster than the latter (S3 and S4 Tables). Based on the systematic performance evaluation as well as our experience of applying the method to simulated and empirical data with typical sample sizes for *in-vitro* experiments, even the slower method can process hundreds and thousands of feature-SNP pairs within a day through parallel computing on a cluster. Since our analysis suggested occasional, moderate inaccuracies of MAP estimation followed by Laplace approximation for SNPs without minor allele homozygotes, we recommend using this computational approach only for optimizing the hyperparameters unless SNPs lacking minor allele homozygotes are excluded from the analysis. Our computational approaches provide sufficient efficiency for analyzing response molecular QTLs representing a pre-selected set of feature-SNP pairs with significant G×T interactions.

A variety of statistical methods have been developed to identify G×T and G×E interactions [2]. Despite being methodologically simple, the “stratified” approach used in earlier studies [3,4] is limited in that an uncertainty measure is not readily available for a set difference and that it cannot detect subtle interactions where the association between the phenotype and the genotype is significant in both conditions but to different extents. Moreover, there is no sharing of information between the conditions even if the data is collected from identical or overlapping sets of individuals. Although the “delta” approach [3,5–7] can be better powered than other approaches in some situations, it removes the information regarding the genotype-phenotype association in individual conditions. Moreover, for a given genotype, it requires phenotypic measurements in both control and treated conditions and, thus, is not applicable to a setting, such as that of clinical trials, where an individual of a given genotype either receives or does not receive treatment. The “interaction” approach [8–12] is one of the most commonly used methods since this approach does not require paired data and potentially exhibits increased power, the latter of which may result from joint modeling of the phenotype data in the control and treated conditions. Our method is built on this type of approach.

The proposed BMS approach is distinct from the large majority of existing methods of G×T and G×E analyses in that the goal is to classify different types of interactions, whereas existing methods focus on detection with some notable exceptions, including those developed by Barber *et al.* [15] and Maranville *et al.* [16]. The pattern of posterior probabilities of G×T interaction resulting from our approach provides different strengths of evidence for different G×T interactions, which are intuitive and interpretable. It is straightforward to extract practically relevant information through post-processing. This includes the posterior probability of an event where the genotype effect is induced or altered by treatment, an event where the treatment effect is restricted to a specific genotype or varies depending on the genotype, and an event where the treatment has the opposite effect (i.e., effects with different signs) depending on the genotype. Moreover, it is possible to obtain probabilities of association patterns with sign constraints of interest. The feature-SNP pairs can then be prioritized based on the probability of specific association patterns, for example, cases of enhancer priming where activation of co-factors is required to induce the transcriptional effect of an enhancer on a gene promoter [10]. As such, researchers could obtain a set of feature-SNP pairs by setting a posterior probability threshold that is appropriate for their goal. The set can then be used for bioinformatic analyses to gain further biological insights, analogously to previous studies [10,11]. For example, the genes in the set can be examined for enrichment in biological pathways. The DNA regions around the SNPs in the set can be investigated for enriched sequence motifs. The cCREs in the set can be analyzed for informative characteristics, such as distances from the transcription start sites of the neighboring genes. Our method now allows for this type of analysis with a greater variety of association patterns than the approaches taken in the previous studies. Furthermore, a small number of top-ranked variants based on the posterior probabilities can be investigated in *in-vitro* cell systems using techniques, such as CRISPRi/a perturbation [44].

Our method differs from the existing probabilistic approach developed by Maranville *et al.* [16], which was an extension and improvement of earlier work by Barber *et al.* [15], at least in the following aspects. First, in our model specification, the control and treated conditions are asymmetric in that the former is considered basal, unlike in the bivariate outcome model used in the existing method [16]. This specification is based on the assumption that treatment with stimuli tends to induce a genetically regulated response rather than suppress it, as exemplified by a previous study in immune cells [10]. Nonetheless, in a situation where generic variation is expected to be suppressed, the coding of the treatment indicator variable can be reversed. Second, our method does not require paired data and, thus, can accommodate a wider range of settings. Third, our model assumes equal variances for residual errors on the log scale between the two conditions, whereas the existing method allows heterogeneous variances. That said, in principle, our model could be trivially modified to accommodate such additional heterogeneity. Fourth, we account for the possible error correlation structure between samples derived from the same donor by including random effects, whereas the existing method [16] accommodates the correlation structure by assuming that the errors follow a bivariate Normal distribution with a non-diagonal covariance matrix. Last and importantly, our method accounts for the inherent relationship between the genotype and molecular count phenotype using nonlinear regression while accounting for heteroskedasticity.

Prior specification is a key consideration in Bayesian analysis. BMS requires prior on the candidate models (model prior) as well as on the model parameters (effect prior) (Methods). In BMS, as in other Bayesian analyses that involve computation of the marginal likelihood, the effect prior requires proper calibration. Our extensive simulation analysis shows moderate prior sensitivity (S11, S12, and S13 Figs) [37], suggesting that data-driven prior elicitation, such as our use of empirical Bayes, can be desirable. Although our current implementation uses a grid search, this process can be made more efficient by employing other optimization techniques, such as a coordinate ascent algorithm [45]. For our model prior, we place a uniform prior as in previous studies using BMS for similar purposes [25], and we consider this an easily interpretable default for our setting. Nonetheless, other lines of research using BMS suggest advantages of model priors that are data-driven [46,47]. Although the advantage of eliciting the model prior in a data-driven manner was demonstrated in the context of *detecting* associations, the approach may also have advantage in the *classification* context described here. Determining how best model prior is elicited for our BMS framework is an important future direction.

Although our focus here was on molecular count phenotypes, our method can be applied to a broader range of phenotypes. In particular, certain continuous phenotypes can be adequately modeled using linear regression as an approximation (Eqs (5) and (12)). For this class of phenotypes, G×T interactions, or more broadly, G×E interactions, can be efficiently classified if random effects need not be modeled. BMS can then take advantage of the exact analytical form of the marginal likelihood, which is available for the linear models with a particular prior specification [48]. Unlike the method we proposed in the current work, this method does not require sampling or optimization and, thus, provides superior computational efficiency albeit with less flexibility. We note that such a method has the potential to contribute to the study of general G×E interactions in human genetics by allowing a comprehensive exploration of observational data, including those with large sample sizes. By contrast, this method may not be suited for pharmacogenetics applications since, unlike pharmacogenomics (i.e., G×T studies on molecular phenotypes), detecting G×T interactions for disease phenotypes is notoriously challenging.

Another possible extension of our method is to accommodate a continuous treatment covariate, such as stimulant concentration or time after stimuli [9,49,50], or more than two discrete treatment conditions [10]. In particular, it would be of interest to classify G×T interactions in time-course eQTL mapping data, termed dynamic eQTLs [9,49,50]. Such methods can be implemented in BMS with linear regression models containing a continuous time variable in place of a categorical condition variable. Non-monotonic relationships can be captured using second or higher-order polynomials [50]. A potential limitation in this approach is that the number of models increases exponentially with respect to the number of treatment/time covariates. Nonetheless, the approach would be computationally feasible with a linear approximation of the genotype-phenotype relationship, for which an analytical solution is available.

## Conclusion

We have developed a statistical framework for classifying G×T interactions for molecular phenotypes that facilitates the interpretation and prioritization of response molecular QTLs. Our method takes a set of response molecular QTLs identified by a standard method and assigns posterior probabilities to different types of G×T interactions using a BMS approach. In our simulation experiments, we compared linear and nonlinear regression of log-scale counts and observed moderate but consistent performance advantage of the latter over the former. We then applied our framework to experimental data generated in *in-vitro* cell system derived from genetically diverse donors with and without growth stimulation. Although both linear and nonlinear regression approaches were successful in recovering the G×T signals, we observed individual examples where the latter captured the data more adequately than the former and the two approaches resulted in different posterior probability patterns. Our method revealed different strengths of evidence for different types of G×T interactions across feature-SNP pairs. This type of information is not provided by existing methods for analyzing response molecular QTLs and can be effective for the interpretation and prioritization of genetic variants underlying the diversity in treatment response among individuals.

## Methods

### Datasets and preprocessing

Primary human neural progenitor cell (hNPC) genotypes, RNA-seq, and ATAC-seq data were obtained from a previous study [11] and were based on the GRCh38 genome assembly. The genotype data was coded as {0,1,2} to represent the number of minor (alternative) alleles and contained 78 and 72 donors for the RNA- and ATAC-seq data, respectively. Among the previously generated data with multiple treatments, we focused on the vehicle and CHIR treatments as control and treated conditions, respectively. The dataset contained one sample per combination of donor and treatment condition. That is, the donors were shared in both control and treated conditions, and there was no missingness.

For assessing the allelic additivity assumption, we considered a set of feature-SNP pairs for which a significant association between the genotype and the phenotype was identified at least in one of the control and treated conditions by the previous study [11]. We excluded feature-SNP pairs for which the number of donors at any genotype level is zero since such data is not suited to the larger model that assumes three genotype levels (Eq (7)). For eQTL and caQTL data, the filtering resulted in 3073 and 83488 feature-SNP pairs, respectively. For Bayesian model selection (BMS), we considered 98 response eQTLs as well as 1775 response caQTLs, identified by the previous study [11]. For both types of analysis, we only included SNPs on autosomes.

In the previous study, the count data was transformed using a variance stabilizing transformation other than the log ⁡ transformation [11]. To achieve compatibility with the nonlinear model that assumes the log ⁡ transformation (Eq (11)), we reprocessed the raw count data according to previous studies on the allelic additivity [18,19]. Briefly, we first scaled the original count data as


$$c_{i,g}=\frac{d_{i,g}}{l_{i}∕G}\bar{d},$$

where *i* = 1 , *…* , *n* indexes the samples, *g* = 1 , *…* , *G* indexes the features, such as genes and ATAC-seq peak regions, *d_i,g_* denote elements of the original count matrix, $l_{i}=\sum_{g=1}^{G}d_{i,g}$ be the library size for sample *i*, and $\bar{d}=\frac{\sum_{i=1}^{n}\sum_{g=1}^{G}d_{i,j}}{Gn}$ is the overall mean. We note that the experiments have similar sequencing depth and, thus, that the variance of the scaled counts is similar to that of the original counts. For a given gene *g*, we transformed *c_i_* as


$$y_{i}=\log(c_{i}+1),$$

where *y_i_* is the scaled, log ⁡ -transformed count data. Note that the subscript *g* is omitted for simplicity. The total numbers of genes and ATAC peak regions were 22354 and 172887, respectively. To aid comparisons between competing methods, we additionally transform count data $y={\left\{y_{i}\right\}}_{i=1}^{n}$ using the rank inverse normal transformation (RINT),


$$\text{RINT}(y_{i})=\Phi^{-1}\left(\frac{r_{i}-\frac{1}{2}}{n}\right),$$

where *r_i_* is the rank order of *y_i_* in **y**, and Φ is the cumulative density function of the standard Normal distribution. For response molecular QTL mapping and BMS, we applied the transformation to the phenotype data residualized with respect to confounding variables (see **SI Text**).

### Assessing the allelic additivity assumption in a given condition

For assessing the allelic additivity assumption in a condition-specific manner, we computed the maximum likelihood estimates (MLE) of the model parameters based on the previously developed nonlinear model [18,19] (Eqs (s) and (3), and (4)) and compared the values with those obtained from a standard linear model for QTL mapping with a continuous genotype variable (Eq (5)) as well as those from a larger model with a categorical genotype variable (Eq (7)). Note that the model in Eq (7) subsumes those in Eqs (2) and (5). In our notation, for a given condition and the *i*-th donor (*i* = 1 , *…* , *n*), the nonlinear model representing the allelic additivity can be written as


$$\begin{array}{ccc} y_{i} & =f_{g}(g_{i})+\varepsilon_{i}, & \end{array}$$ (2)

$$\begin{array}{ccc} \varepsilon_{i} & \overset{iid}{∼}N(0,\sigma^{2}), & \end{array}$$ (3)

$$\begin{array}{ccc} f_{g}(g_{i}) & =\log\left(\left(1-\frac{g_{i}}{2})\exp(\beta_{0})+(\frac{g_{i}}{2})\exp(\beta_{0}+2\beta_{g}\right)\right), & \end{array}$$ (4)

where *n* is the number of donors, *y_i_* is the log ⁡ -transformed molecular count data, *g_i_* is the genotype coded as {0,1,2} or the imputation-based allelic dosage $g_{i}\in [0,2]$, *ε_i_* is the residual error, and *σ*^2^ is the residual error variance, and *β*_0_ is the intercept. The parameter *β*_g_ represents the half the difference in the expected values of *y_i_* for the major allele homozygote vs the minor allele homozygote. That is, the quantities can be defined as


$$\begin{array}{llll} f_{g}(g=0) & =\beta_{0},\; & & \;\\ f_{g}(g=2) & =\beta_{0}+2\beta_{g},\; & & \; \end{array}$$

but where $f_{g}(g=1)\neq \beta_{0}+\beta_{g}$ for any non-zero *β_g_*. Our definitions and interpretations of the coefficients differ from those in the previous studies [18,19] to make the scales comparable to those in the standard linear model for QTL mapping, which can be cast as


$$\begin{array}{ccc} y_{i} & =\beta_{0}+\beta_{g}g_{i}+\varepsilon_{i}, & \\ \varepsilon_{i} \end{array}$$ (5)

$$\begin{array}{cc} \overset{iid}{∼}N(0,\sigma^{2}). & \end{array}$$ (6)

The larger model is cast as


$$\begin{array}{ccc} y_{i} & =\beta_{0}1(g_{i}=0)+\beta_{1}1(g_{i}=1)+\beta_{2}1(g_{i}=2)+\varepsilon_{i}, & \\ \varepsilon_{i} \end{array}$$ (7)

$$\begin{array}{cc} \overset{iid}{∼}N(0,\sigma^{2}), & \end{array}$$ (8)

where **1**(⋅) is an indicator function, and parameters *β*_0_, *β*_1_, and *β*_2_ are, respectively, the expected values of *y_i_* for donors with the homozygous major allele, the heterozygote, and the homozygous minor allele. Note that, for a given condition, the hNPC data contained one measurement per donor and that, for the ease of exposition, confounding factors are omitted (for model formulation with confounding factors, see **SI Text**).

### Extending the allelic additivity model forG×Tanalysis

The nonlinear allelic additivity model above can be modified for G×T analysis as follows. For the *i*-th sample (*i* = 1 , *…* , *n*), we model


$$\begin{array}{ccc} y_{i} & =f_{g,t}(g_{i},t_{i})+\varepsilon_{i}, & \\ \varepsilon_{i} \end{array}$$ (9)

$$\begin{array}{cc} \overset{iid}{∼}N(0,\sigma^{2}), & \end{array}$$ (10)

$$\begin{array}{cccc} f_{g,t}(g_{i},t_{i}) & =\log((1-\frac{g_{i}}{2})(1-t_{i})\exp(\beta_{0}) & & \\ \;+(\frac{g_{i}}{2})(1-t_{i})\exp(\beta_{0}+2\beta_{g}) & & \\ \;+(1-\frac{g_{i}}{2})(t_{i})\exp(\beta_{0}+\beta_{t}) & & \\ \;+(\frac{g_{i}}{2})(t_{i})\exp(\beta_{0}+2\beta_{g}+\beta_{t}+2\beta_{g\times t})), & & \end{array}$$ (11)

where *y_i_*, *g_i_*, *ε_i_*, *σ*^2^, and *β*_0_ are defined as earlier, *t_i_* denotes an indicator variable for a treatment, *β_g_* now represents the half the difference in the expected values of *y_i_* for the major allele homozygote vs the minor allele homozygote *under the control condition*, *β_t_* is the difference in the expected values of *y_i_* in the control condition vs the treated condition *for the major allele homozygote*, and $\beta_{g\times t}$ allows the effect of genotype to vary between treatment groups. That is, the quantities can be defined as


$$\begin{array}{llll} f_{g,t}(g=0,t=0) & =\beta_{0},\; & & \;\\ f_{g,t}(g=2,t=0) & =\beta_{0}+2\beta_{g},\; & & \;\\ f_{g,t}(g=0,t=1) & =\beta_{0}+\beta_{t},\; & & \;\\ f_{g,t}(g=2,t=1) & =\beta_{0}+2\beta_{g}+\beta_{t}+2\beta_{g\times t}.\; & & \; \end{array}$$

Our definitions and interpretations of the coefficients differ from those in the previous studies [18,19] to make the scales comparable to those in the standard linear model for G×T analysis, namely


$$\begin{array}{ccc} y_{i} & =\beta_{0}+\beta_{g}g_{i}+\beta_{t}t_{i}+\beta_{g\times t}g_{i}t_{i}+\varepsilon_{i}, & \\ \varepsilon_{i} \end{array}$$ (12)

$$\begin{array}{cc} \overset{iid}{∼}N(0,\sigma^{2}). & \end{array}$$ (13)

Note that, for the ease of exposition, confounding factors are omitted (for model formulation with confounding factors, see **SI Text**).

### Bayesian model selection

To classify the types of G×T interactions, we propose a Bayesian model selection approach considering eight models where the $\beta_{g}$, $\beta_{t}$, and $\beta_{g\times t}$ parameters are either zero or non-zero in Eq (11). The approach consists of four steps as in Bayesian approaches more generally. First, we specify the likelihood and prior probabilities. Second, we compute the posterior probabilities. Third, we summarize the posterior probabilities of interest. Fourth, optionally, we make a decision based on a loss function.

Using a vector of indicators, $m=(m_{g},m_{t},m_{g\times t})\in M={\left\{0,1\right\}}^{3}$, that specifies one of the eight candidate models in the set *M*, we cast the model as


$$\begin{array}{ccc} y_{i} & =f_{g,t}(g_{i},t_{i})+\varepsilon_{i}, & \end{array}$$ (14)

$$\begin{array}{ccc} \varepsilon_{i} & \overset{iid}{∼}N(0,\sigma^{2}), & \end{array}$$ (15)

$$\begin{array}{cccc} f_{g,t,m}(g_{i},t_{i},m) & =\log((1-\frac{g_{i}}{2})(1-t_{i})\exp(\beta_{0}) & & \\ \;+(\frac{g_{i}}{2})(1-t_{i})\exp(\beta_{0}+2m_{g}\beta_{g}) & & \\ & \;+(1-\frac{g_{i}}{2})(t_{i})\exp(\beta_{0}+m_{t}\beta_{t}) & & \end{array}$$

$$\begin{array}{cc} \;+(\frac{g_{i}}{2})(t_{i})\exp(\beta_{0}+2m_{g}\beta_{g}+m_{t}\beta_{t}+2m_{g\times t}\beta_{g\times t})), & \end{array}$$ (16)

where $y_{i}$, $g_{i}$, $t_{i}$, $\varepsilon_{i}$, and $\sigma^{2}$ are defined as earlier and $\beta ={\left(\beta_{0},\beta_{g},\beta_{t},\beta_{g\times t}\right)}^{T}$ denotes the regression coefficients. For concreteness, we write


$$M=\{m_{1},m_{2},m_{3},m_{4},m_{5},m_{6},m_{7},m_{8}\},$$

where


$$\begin{array}{llll} m_{1} & =(0,0,0),\;m_{2}=(1,0,0),\;m_{3}=(0,1,0),\;m_{4}=(1,1,0),\; & & \;\\ m_{5} & =(0,0,1),\;m_{6}=(1,0,1),\;m_{7}=(0,1,1),\;m_{8}=(1,1,1).\; & & \; \end{array}$$

Using *j* to index the models, we have


$$m_{j}=(m_{g,j},m_{t,j},m_{g\times t,j}),$$

for *j* = 1 , *…* , 8. The conditional joint likelihood is


$$\begin{array}{ccc} y|g,t,m,\beta ,\sigma^{2}∼N_{n}(f_{g,t,m}(g,t,m),\sigma^{2}I), & & \end{array}$$ (17)

where $y\in {\mathbb{R}}^{n}$ denotes a vector of log ⁡ -transformed molecular count data, $g\in {\mathbb{R}}^{n}$ denotes a vector of genotypes, $t\in {\mathbb{R}}^{n}$ denotes a vector of treatment indicators, $\beta ={\left(\beta_{0},\beta_{g},\beta_{t},\beta_{g\times t}\right)}^{T}$ and $f_{g,t,m}(g,t,m)={\left(f_{g,t,m}(g_{1},t_{1}),\ldots ,f_{g,t,m}(g_{n},t_{n})\right)}^{T}\in {\mathbb{R}}^{n}$ denotes a vector of mean values in the untransformed scale. In an exact mathematical form, Eq (17) corresponds to


p(y|g,t,m,β,σ2)= ∏i=1n(2πσ2)−12 exp ⁡  {−12σ−2(yi−fg,t,m(gi,ti,m))2}.

A BMS with linear regression can be formulated by replacing Eq (14) with


$$\begin{array}{llll} y_{i} & =\beta_{0}+m_{g}\beta_{g}g_{i}+m_{t}\beta_{t}t_{i}+m_{g\times t}\beta_{g\times t}g_{i}t_{i}+\varepsilon_{i}.\; & & \; \end{array}$$

In BMS, it is necessary to specify a prior probability that each of the model is correct, $Pr(m=m_{j})$, and prior distributions for the non-zero parameters in the models, $\beta_{g}$, $\beta_{t}$, and $\beta_{g\times t}$. We call the former and latter model and effect priors, respectively. For the model prior, by default, we place the uniform prior probability across the eight models, which corresponds to


$$Pr(m=m_{j})=\frac{1}{8}$$

for *j* = 1 , *…* , 8, where *j* indexes the models. This choice is reasonably justified since we apply BMS to a pre-selected set of feature-SNP pairs that are likely to have significant associations. For the effect prior, we use the Normal-Gamma prior, which is commonly used due to its conjugacy for linear models. That is, we set


$$\begin{array}{cccc} \sigma^{2} & ∼IG\left(\frac{\kappa }{2},\frac{\nu }{2}\right), & & \end{array}$$

$$\begin{array}{cccc} \beta_{0}|\sigma^{2} & ∼N(0,\phi_{0}^{2}\sigma^{2}), & & \end{array}$$

$$\begin{array}{ccc} \beta_{g}|\sigma^{2} & ∼N(0,\phi_{g}^{2}\sigma^{2}), & \end{array}$$ (18)

$$\begin{array}{cccc} \beta_{t}|\sigma^{2} & ∼N(0,\phi_{t}^{2}\sigma^{2}), & & \end{array}$$ (19)

$$\begin{array}{cccc} \beta_{g\times t}|\sigma^{2} & ∼N(0,\phi_{g\times t}^{2}\sigma^{2}), & & \end{array}$$ (20)

where *κ*, *ν*, $\phi_{0}$ and $\phi ={\left(\phi_{g},\phi_{t},\phi_{g\times t}\right)}^{T}$ are the prior hyperparameters. For the residual variance $\sigma^{2}$ and the intercept $\beta_{0}$, nearly non-informative priors are used by setting $\phi_{0}=10^{-\frac{3}{2}}$, $\kappa =\nu =2\times 10^{-3}$. The hyperparameters $\phi =(\phi_{g},\phi_{t},\phi_{g\times t})$ control the effect sizes relative to the residual error standard deviation and need to be properly calibrated for the computation of the marginal likelihood. We employ an empirical Bayes approach where the hyperparameter values maximizing the sum of the marginal likelihood are sought by a grid search.

In our BMS procedure, the second step corresponds to computing the posterior probabilities of the model, $Pr(m=m_{j}|y)\propto p(y|m=m_{j})\;Pr(m=m_{j})$, which is proportional to the product of the marginal likelihood conditional on the model, *p* ( *y* ∣ *m* ) , and the prior for the models, $Pr(m=m_{j})$. The marginal likelihood is defined as


$$p(y|m_{j})=\int \int p(y|\beta_{j},\sigma^{2})p(\beta_{j}|\sigma^{2})p(\sigma^{2})\;d\beta_{j}\;d\sigma^{2},$$

for *j* = 1 , *…* , 8. $\beta_{j}$ is a vector consisting of the non-zero coefficients. For example, $\beta_{4}={\left(\beta_{0},\beta_{g},\beta_{t}\right)}^{T}$ and $\beta_{8}={\left(\beta_{0},\beta_{g},\beta_{t},\beta_{g\times t}\right)}^{T}$. The posterior probability of the *j*th model is computed as


$$Pr(m=m_{j}|y)=\frac{p(y|m=m_{j})\;Pr(m=m_{j})}{\overset{8}{\underset{k=1}{\sum }}p(y|m=m_{k})\;Pr(m=m_{k})}.$$

This involves fitting each of the eight models separately. Since the posterior distributions of the parameters and the marginal likelihood, $p(y|m=m_{j})$, are intractable, we obtain approximate values by either of two methods. In the first method, we fit the models using Markov Chain Monte Carlo (MCMC) as implemented in the R package “rstan” [51] and compute the marginal likelihood using bridge sampling [52]. In all analyses presented in this manuscript, for MCMC, we used the default setting of the sampling() function in the rstan package. That is, we set the number of chains to four. For each chain, the numbers of burn-in and post burn-in samples were set to 1000. In the second method, we obtain a maximum *a posteriori* (MAP) estimate using optimization and compute the marginal likelihood using Laplace’s method [48,53] (see **SI Text** for details). After computing the posterior probabilities of the eight models, in the third step of our BMS procedure, we summarize them in a way that is practically informative. We describe this step in detail in **Refining posterior inference through post-processing**. For the optional fourth step in BMS, we make a decision using a 0-1 loss function, which corresponds to selecting the model with the highest posterior probability $Pr(m=m_{j}|y)$ (a maximum *a posteriori*, MAP, estimation).

In principle, this procedure is equivalent to fitting a Bayesian variable selection regression (BVSR) model where spike-and-slab priors are placed on the regression coefficients and all possible models are simultaneously considered in a sampling process [54]. For this type of model, sampling can be trapped in one model, resulting in poor mixing. By contrast, in our approach, all models are separately fit, which is possible since only a small number of models are considered.

#### Conditional and model averaged effect posteriors.

It is straightforward to obtain model-averaged posterior distributions of the effect sizes from the corresponding conditional distributions [55] as


$$\begin{array}{llll} p(\beta_{g}|y) & =\overset{8}{\underset{j=1}{\sum }}\;p(\beta_{g}|m_{j},y)\;Pr(m=m_{j}|y),\; & & \;\\ p(\beta_{t}|y) & =\overset{8}{\underset{j=1}{\sum }}p(\beta_{t}|m_{j},y)\;Pr(m=m_{j}|y),\; & & \;\\ p(\beta_{g\times t}|y) & =\overset{8}{\underset{j=1}{\sum }}\;p(\beta_{g\times t}|m_{j},y)\;Pr(m=m_{j}|y).\; & & \; \end{array}$$

### Refining posterior inference through post-processing

In the third step of our BMS procedure, we summarize the posterior probability of interest.

#### Aggregating model categories.

One approach is to compute posterior probability of an aggregated category, which can be of practical relevance. This is possible for either of the approaches to compute posterior probabilities of the models, namely, MCMC followed by bridge sampling and MAP estimation followed by Laplace approximation (see **Bayesian model selection** above). Since the eight models are mutually exclusive, posterior probabilities of aggregated categories can be obtained by summing posterior probabilities of the corresponding models. For example, the posterior probability of models where the genotype coefficient is non-zero is computed as the sum of the probability of (1,0,0), (1,1,0), (1,0,1), and (1,1,1). We denote this aggregated category by (1,∗,∗). Likewise, a category of models with non-zero G×T interaction can be written as (∗,∗,1), which we refer to the “interaction” effect category. Additionally, the following aggregated categories can be considered. First, the “induced” genotype effect category, corresponding to (0,∗,1), represents an event where the association between the genotype and phenotype emerges only upon treatment. Second, the “altered” genotype effect, corresponding to (1,∗,1), represents an event where the genotype-phenotype association exists in the absence of treatment but is the strength of the association is altered upon treatment. Third, the “restricted” treatment effect, corresponding to (∗,0,1), represents an event where the treatment only affects individuals with genotype levels 1 and 2. Fourth, the “varying” treatment effect, corresponding to (∗,1,1), represents an event where the treatment affects all individuals but to different extents. In practical settings, the “interaction,” “induced,” “altered,” “restricted,” and “varying” effect categories may be more relevant than each of the eight models considered individually.

#### Posterior probability of crossover interaction

From MCMC samples, it is possible to compute posterior probability of the crossover interaction as follows. Let *A* be the event of a crossover interaction. Then, for *j* = 1 , *…* , 8, we have


$$\begin{array}{llll} & Pr(A|m_{j})\; & & \;\\ = & \;Pr(\left(f_{g,t,m}(g=2,t=0,m=m_{j})-f_{g,t,m}(g=0,t=0,m=m_{j})\right)\; & & \;\\ & \;\times \left(f_{g,t,m}(g=0,t=1,m=m_{j})-f_{g,t,m}(g=2,t=1,m=m_{j})\right)<0),\; & & \; \end{array}$$

where $f_{g,t,m}$ is defined as in Eq (16). Since the crossover interactions can appear only when **m** equals **m**_7_ or **m**_8_ and thus $Pr(A|m_{j})=0$ for *j* = 1 , *…* , 6, by the law of total probability, we have


$$\begin{array}{llll} & Pr(A)\; & & \;\\ = & \overset{8}{\underset{j=1}{\sum }}Pr(m=m_{j})\;Pr(A|m=m_{j})\; & & \;\\ = & \;Pr(m=m_{7})\;Pr\left(\beta_{t}(\beta_{t}+\beta_{g\times t})<0|m=m_{7}\right)\; & & \;\\ & \;+Pr(m=m_{8})\;Pr\left(\beta_{t}(\beta_{t}+\beta_{g\times t})<0|m=m_{8}\right).\; & & \; \end{array}$$

For *j* = 7 , 8, we approximated the conditional posterior probability as


$$\begin{array}{llll} & Pr(\beta_{t}(\beta_{t}+\beta_{g\times t})<0|m=m_{j})\; & & \;\\ \approx & \;\frac{1}{S-B}\overset{S}{\underset{s=B+1}{\sum }}1\left(\beta_{t}^{\left(s\right)}{\left(\beta_{t}^{\left(s\right)}+\beta_{g\times t}\right)}^{\left(s\right)}<0|m=m_{7}\right),\; & & \; \end{array}$$

where *S* is the number of MCMC samples, *B* is the number of burn-in iterations, $\beta_{t}^{\left(s\right)}$ and $\beta_{g\times t}^{\left(s\right)}$ denote samples at the *s*-th iteration. We set *S*=2000 and *B*=1000.

#### Subcategories of models based on the sign of effects.

To account for the sign of the effect sizes for a given model, we examined the sign of the posterior means or MAP estimates of non-zero coefficients and assigned the same amount of probability to the model with corresponding sign combination and zero probability to others. For example, if $m=m_{7}=(0,1,1)$, we examined the sign of the estimates $\hat{\beta_{t}}$ and ${\hat{\beta }}_{g\times t}$ given that the seventh model **m**_7_ was correct. If ${\hat{\beta }}_{t}<0$ and ${\hat{\beta }}_{g\times t}>0$, we set


$$Pr(m^{*}=(0,-1,1))=Pr(m=m_{7})$$

and


$$Pr(m*=(0,1,1))=Pr(m^{*}=(0,1,-1))=Pr(m^{*}=(0,-1,-1))=0,$$

where $m^{*}={\left\{-1,0,1\right\}}^{3}$ is a vector specifying the 27 model categories.

### Generating data for simulations

For simulation experiments, we generated 1000 sets of feature-SNP pairs for each of the eight models specified in Eq (16), summing to 8000 feature-SNP pairs in total. Each simulated feature-SNP pair comprised the genotypes for 80 individuals, with these drawn from a Binomial distribution, *Binom* ( *n* = 2 , *p* = *π* ) , where π∼Uniform(0.05,0.5), corresponding to minor allele frequency (MAF) ranging from 0.05 to 0.5, and simulated normalized count data $y^{*}={\left\{y_{i}^{*}\right\}}_{i=1}^{160}$ (i.e., the phenotype vector). In generating a simulated phenotype vector from a given model, we fixed the intercept and residual error standard deviation to $\beta_{0}=0$ and *σ* = 1, set the donor random effect variance $\sigma_{u}^{2}$ to 0.2, and set the kernel matrix **K** to the identity. The regression coefficients were drawn from Normal distributions as in Eq (18), (19), and (20) with $\phi {\left(\phi_{g},\phi_{t},\phi_{g\times t}\right)}^{T}={\left(1.5,2.0,1.0\right)}^{T}$, with these values based on the results of hyperparameter calibration using the hNPC eQTL data. This generated 80 phenotype values for each of the control and treated conditions (160 in total) based on Eqs (14) and (16). For evaluating relative performance of log ⁡ -NL, log ⁡ -LM, and RINT-LM, we analyzed the entire set of 8000 simulated feature-SNP pairs using both MAP estimation followed by Laplace approximation to get posterior model probabilities, and MCMC followed by bridge sampling to compute the posterior probability of crossover interaction. In the other MCMC analyses comparing the three approaches, we used 100 feature-SNP pairs, summing to 800 in total, due to the computational cost. In our comparisons of estimating the marginal likelihoods via Laplace approximation vs bridge sampling, we used 10 feature-SNP pairs, summing to 80 in total, albeit for 125 combinations of hyperparameter values.

### Additional methods

Additional details are in the **Supplementary methods** section in SI Text, specifically in the following subsections: **The log-NL model of G×T analysis**, **Review of previously developed methods**, **Computing the marginal likelihood by Laplace approximation**, **Hyperparameter optimization using empirical Bayes**, and **Including covariates**.

## Supporting information

S1 Fig Assessing the allelic additivity assumption in hNPCs. A. Scatterplots comparing the maximized likelihood between nonlinear and linear regression for 3073 gene-SNP pairs under the control condition. B. The same as in A but between the model with a categorical genotype variable consisting of three levels and nonlinear regression. C. The same as in A but between the model with a categorical genotype variable and linear regression. D. The same as in A but under the treated condition. E. The same as in B but under the treated condition. F. The same as in C but under the treated condition. G. Scatterplots comparing the maximized likelihood between nonlinear and linear regression for 83488 cCRE-SNP pairs under the control condition. H. The same as in G but between the model with a categorical genotype variable and nonlinear regression. I. The same as in G but between the model with a categorical genotype variable and linear regression. J. The same as in G but under the treated condition. K. The same as in H but under the treated condition. L. The same as in I but under the treated condition. (PDF)

S2 Fig ROC curves assessing the performance of BMS with log ⁡ -NL, log ⁡ -LM, and RINT-LM for the no-G×T, induced, and altered categories using MCMC and bridge sampling. Shown are results from 800 simulations without random effect, which we call scenario 1 (A), those with donor random effect in model fitting but not in data generation (scenario 2) (B), those with donor random effect in data generation but not in model fitting (scenario 3) (C), and those with donor random effect in both data generation and model fitting (scenario 4) (D). See the repository [37] for results of BMS using MAP estimation and Laplace approximation. (PDF)

S3 Fig Calibration of BMS with log ⁡ -NL, log ⁡ -LM, and RINT-LM for the no-G×T, induced, and altered categories using MCMC and bridge sampling. The *x*- and *y*-axis represent the posterior probability and the fraction of the corresponding events, respectively. The results from 800 simulations are grouped into ten equally-spaced bins. The vertical bars represent the standard errors assuming a binomial distribution. The panels A to D show the results for scenarios 1 to 4, which are defined in the legend to S2 Fig. See the repository [37] for results of BMS using MAP estimation and Laplace approximation. (PDF)

S4 Fig Stratified histograms of posterior probability of the eight models obtained by BMS with log ⁡ -NL using MCMC and bridge sampling. In each panel, the rows and columns represent the data-generating and posterior mode models, respectively. The panels A to D show the results for scenarios 1 to 4, which are defined in the legend to S2 Fig. See the repository [37] for other simulation scenarios and results of BMS using MAP estimation and Laplace approximation. (PDF)

S5 Fig Partial ROC curves assessing the performance of BMS with log ⁡ -NL, log ⁡ -LM, and RINT-LM for the no-G×T, induced, and altered categories using MCMC and bridge sampling. The panels A to D show the results for scenarios 1 to 4, which are defined in the legend to S2 Fig. See the repository [37] for results of BMS using MAP estimation and Laplace approximation. (PDF)

S6 Fig Posterior probability of the correct and incorrect models for aggregated categories obtained by BMS using MCMC and bridge sampling. Violin plots for comparing the performance of BMS with log ⁡ -NL, log ⁡ -LM, and RINT-LM based on the distribution of posterior probability of the correct and incorrect models for the no-G×T, induced, and altered model categories. The closed circles represent median values. The panels A to D show the results for scenarios 1 to 4, which are defined in the legend to S2 Fig. See the repository [37] for results of BMS using MAP estimation and Laplace approximation. (PDF)

S7 Fig Posterior probability of the correct and incorrect models for the eight categories obtained by BMS using MCMC and bridge sampling on data generated without random effect. Violin plots for comparing the performance of BMS with log ⁡ -NL, log ⁡ -LM, and RINT-LM based on the distribution of posterior probability of the correct and incorrect models for each of the eight model categories. The closed circles represent median values. The panels A and B respectively show the results for scenarios 1 and 2, which are defined in the legend to S2 Fig. See the repository [37] for other simulation scenarios and results of BMS using MAP estimation and Laplace approximation. (PDF)

S8 Fig Comparison of effect estimates by Bayesian model averaging with log ⁡ -NL, log ⁡ -LM, and RINT-LM. A. Scatter plots comparing estimation of the genotype, treatment, and G×T interaction effects relative to the residual standard deviation against the true values for scenario 1, which is defined in the legend to S2 Fig. Each point represents each of 8000 feature-SNP pairs. B. The same as in A but for scenario 2. C. The same as in A but for scenario 3. D. The same as in A but for scenario 4. See the repository [37] for results obtained by MAP estimation and Laplace approximation. (PDF)

S9 Fig Comparison of posterior probability at varying hyperparameter values between two computational approaches on synthetic data generated without donor random effect. A. Scatter plots comparing posterior probabilities obtained by MCMC followed by bridge sampling and those obtained by MAP estimation followed by Laplace approximation for scenario 1, which is defined in the legend to S2 Fig. Each point represents each of the eight models for a feature-SNP pair. The values are compared across eight models and 686 feature-SNP pairs for which minor allele homozygotes were present (i.e., 5488 combinations). B. The same as in A but for 114 feature-SNP pairs for which minor allele homozygotes were absent (i.e., 912 combinations). C. The same as in A but for scenario 2. D. The same as in B but for scenario 2. See the repository [37] for other simulation scenarios. (PDF)

S10 Fig Comparison of the sum of the log ⁡ of marginal likelihood across combinations of hyperparameter values between two computational approaches for log ⁡ -NL, log ⁡ -LM, and RINT-LM with and without donor random effect. Scatter plots comparing results obtained by MCMC followed by bridge sampling and those obtained by MAP estimation followed by Laplace approximation. The panels A to D show the results for scenarios 1 to 4, which are defined in the legend to S2 Fig. Each point represents the log ⁡ of the marginal likelihood summed over 80 feature-SNP pairs. The values are compared across 125 combinations of the hyperparameter values (see SI Text for details). (PDF)

S11 Fig ROC curves assessing the impact of the effect prior on the performance of BMS using MCMC and bridge sampling. The colors represent varying hyperparameter values. The restrictive and permissive values represent half and twice of the optimal values, respectively. The panels A to D show the results for scenarios 1 to 4, which are defined in the legend to S2 Fig. In each panel, the rows and columns represent modeling approaches and aggregated categories, respectively. (PDF)

S12 Fig Partial ROC curves assessing the impact of the effect prior on the performance of BMS using MCMC and bridge sampling. The colors represent varying hyperparameter values (see the legend to S11 Fig). The panels A to D show the results for scenarios 1 to 4, which are defined in the legend to S2 Fig. In each panel, the rows and columns represent modeling approaches and aggregated categories, respectively. (PDF)

S13 Fig Assessing the impact of the effect prior on the posterior probability of the correct and incorrect models from analyses without random effect using MCMC and bridge sampling. Violin plots showing the distribution of posterior probability of the correct (A) and incorrect (B) models for each of the eight model categories with varying hyperparameter values (see the legend to S11 Fig). The closed circles represent median values. Shown is the results for scenario 1, which is defined in the legend to S2 Fig. See the repository [37] for other simulation scenarios and results of BMS using MAP estimation and Laplace approximation. (PDF)

S14 Fig Posterior probability of the models with and without accounting for the sign of effect size for the response eQTL data in hNPCs. The heatmaps show the posterior probability of the eight models for the 98 response eQTLs, which represent gene-SNP pairs with significant G×T interactions (A), as well as that of the 27 models accounting for the sign of effect size (B). The rows and columns represent the models and gene-SNP pairs, respectively. The gene-SNP pairs are ordered by *P* values for significant G×T interactions from a previous study [11]. The leftmost column corresponds to the smallest *P* value. (PDF)

S15 Fig Comparison of posterior probability between results with donor random effect and those with polygenic random effect for response eQTLs. Scatter plots comparing results obtained by BMS with polygenic (kinship) random effect and those with donor random effect. Each point represents the posterior probability of a mode for a feature-SNP pair. The values are compared across eight models and 98 feature-SNP pairs (i.e., 784 combinations). (PDF)

S16 Fig Posterior probability of the models with and without accounting for the sign of effect size for the response caQTL data in hNPCs. The same as in S14 Fig but for 1775 response caQTLs. (PDF)

S17 Fig Representative BMS results for the response caQTL data in hNPCs. The same as in Fig 5 but for response caQTLs. (PDF)

S18 Fig Examples of response caQTLs with the crossover interaction in hNPCs. The same as in Fig 6 but for response caQTLs. (PDF)

S1 Text Supplementary methods and results. (PDF)

S1 TableConvergence diagnostics of simulation analyses. Shown in the Convergence column are the maximum values of the Gelman–Rubin statistic ($\hat{R}$) across parameters and feature-SNP pairs. Note that $\hat{R}$ indicates convergence. The Scenario column indicates simulation settings, which are defined in the legend to S2 Fig. The Method column indicates modeling approaches used in the analysis. In the Effect prior column, “optimal” corresponds to the hyperparameter values that maximize the sum of the log-marginal likelihood across feature-SNP pairs. “Restrictive” and “permissive” correspond to half and twice the optimal value, respectively.(CSV)

S2 Table Convergence diagnostics of analyses of experimental data. The Method and Convergence columns are as in S1 Table. The Data column indicates the type of data. (CSV)

S3 Table Runtime and memory usage of BMS using MCMC followed by bridge sampling. Shown are the runtime and memory usage of BMS for five randomly chosen feature-SNP pairs. Each row corresponds to each feature-SNP pair. The Data, Scenario, and Method columns are as in S1 and S2 Tables. The Index column represents the indices of the five feature-SNP pairs. The Mean and SD columns contain the mean values and standard deviations of the runtime in second across 10 replicates. The Memory column contains the maximum resident set size in megabyte across 10 replicates. (CSV)

S4 Table Runtime and memory usage of BMS using MAP estimation followed by Laplace approximation. Shown are the runtime and memory usage of BMS for five randomly chosen feature-SNP pairs. Each row corresponds to each feature-SNP pair. The columns are as in S3 Table. (CSV)

S5 Table Optimal hyperparameter values for simulation. Shown are values of the hyperparameters *ϕ* that maximize the sum of the log ⁡ -marginal likelihood across 8000 feature-SNP pairs. The Method column indicates modeling approaches used in the analysis. The Scenario column indicates simulation settings, which are defined in the legend to S2 Fig. The Genotype, Treatment, and Interaction columns correspond to *ϕ_g_*, *ϕ_t_*, and *ϕ_g×t_*, respectively. (CSV)

S6 Table Optimal hyperparameter values for the hNPC data. Shown are values of the hyperparameters *ϕ* that maximize the sum of the log ⁡ -marginal likelihood across feature-SNP pairs. The Method, Genotype, Treatment, and Interaction columns are as in S5 Table. The Data and Random effect columns indicate the type of data and random effect, respectively. (CSV)

S1 File A frozen version (0.1.0) of classifygxt, the R package implementation. Available also from Zenodo [1]. (ZIP)

## Acknowledgments

We thank Samir Kelada and Yun Li of the University of North Carolina at Chapel Hill for helpful discussion and suggestions on this work.
