# Supplementary material for: Probabilistic classification of gene-by-treatment interactions on molecular count phenotypes
Source: PLoS Genet. 2025 Apr 9;21(4):e1011561. doi: 10.1371/journal.pgen.1011561 (PMC12021428; doi:10.1371/journal.pgen.1011561)
Supplement: S1 Fig — (PDF) [file pgen.1011561.s001.pdf]

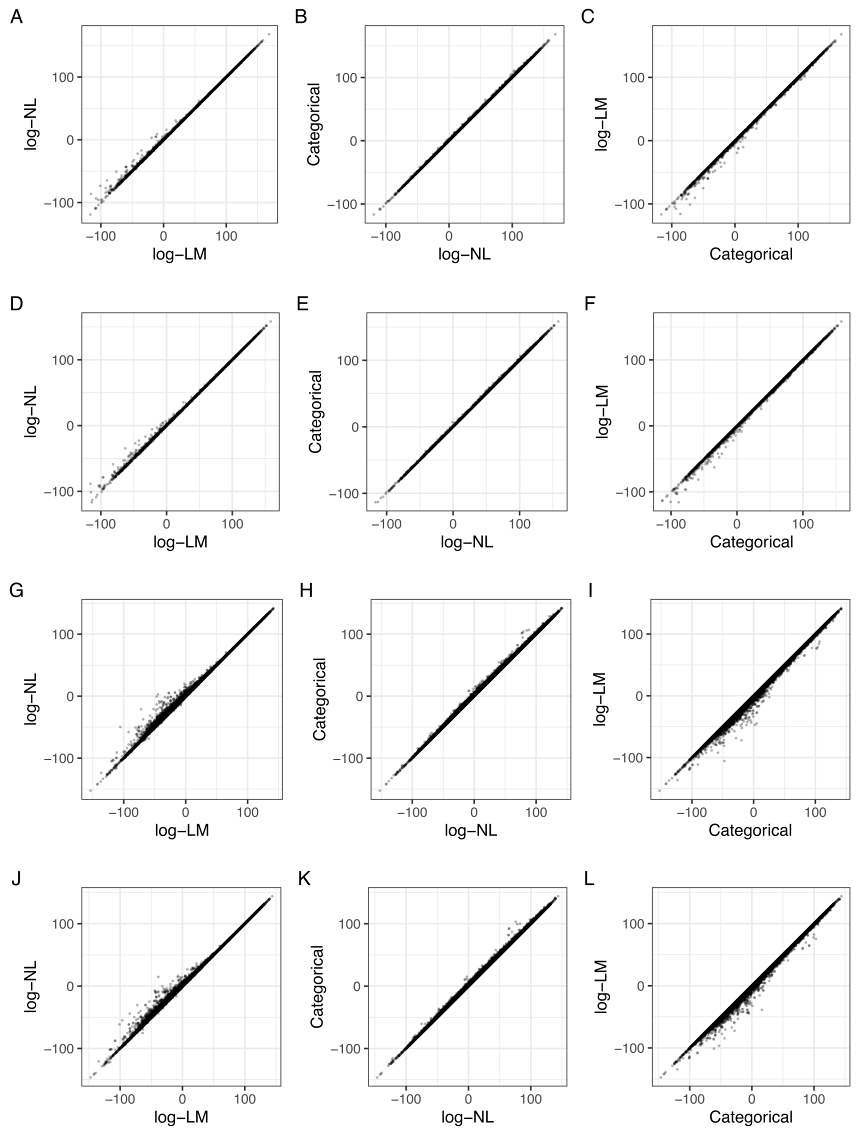

S1 Fig. Assessing the allelic additivity assumption in hNPCs. A. Scatterplots comparing the maximized likelihood between nonlinear and linear regression for 3073 gene-SNP pairs under the control condition. **B.** The same as in **A** but between the model with a categorical genotype variable consisting of three levels and nonlinear regression. **C.** The same as in **A** but between the model with a categorical genotype variable and linear regression. **D.** The same as in **A** but under the treated condition. **E.** The same as in **B** but under the treated condition. **F.** The same as in **C** but under the treated condition. **G.** Scatterplots comparing the maximized likelihood between nonlinear and linear regression for 83488 cCRE-SNP pairs under the control condition. **H.** The same as in **G** but between the model with a categorical genotype variable and nonlinear regression. **I.** The same as in **G** but between the model with a categorical genotype variable and linear regression. **J.** The same as in **G** but under the treated condition. **K.** The same as in **H** but under the treated condition. **L.** The same as in **I** but under the treated condition.
