# Supplementary material for: Probabilistic classification of gene-by-treatment interactions on molecular count phenotypes
Source: PLoS Genet. 2025 Apr 9;21(4):e1011561. doi: 10.1371/journal.pgen.1011561 (PMC12021428; doi:10.1371/journal.pgen.1011561)
Supplement: S2 Fig — (PDF) [file pgen.1011561.s002.pdf]

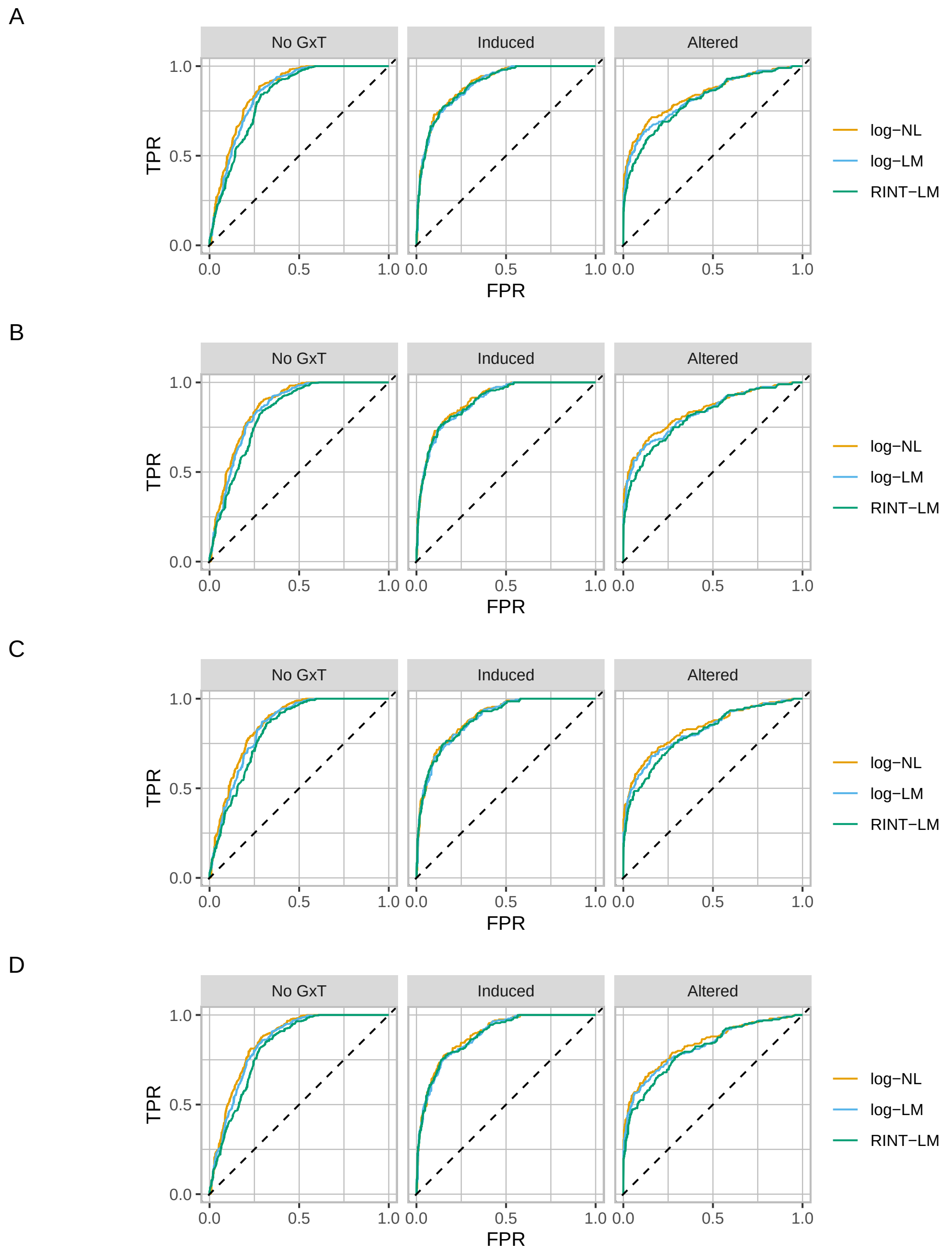

S2 Fig. ROC curves assessing the performance of BMS with log**-NL,** log**-LM, and RINT-LM for the no-G$\times$T, induced, and altered categories using MCMC and bridge sampling.** Shown are results from 800 simulations without random effect, which we call scenario 1 (**A**), those with donor random effect in model fitting but not in data generation (scenario 2) (**B**), those with donor random effect in data generation but not in model fitting (scenario 3) (**C**), and those with donor random effect in both data generation and model fitting (scenario 4) (**D**). See the repository (https://doi.org/10.5281/zenodo.14827827) for results of BMS using MAP estimation and Laplace approximation.
