# Supplementary material for: Probabilistic classification of gene-by-treatment interactions on molecular count phenotypes
Source: PLoS Genet. 2025 Apr 9;21(4):e1011561. doi: 10.1371/journal.pgen.1011561 (PMC12021428; doi:10.1371/journal.pgen.1011561)
Supplement: S3 Fig — (PDF) [file pgen.1011561.s003.pdf]

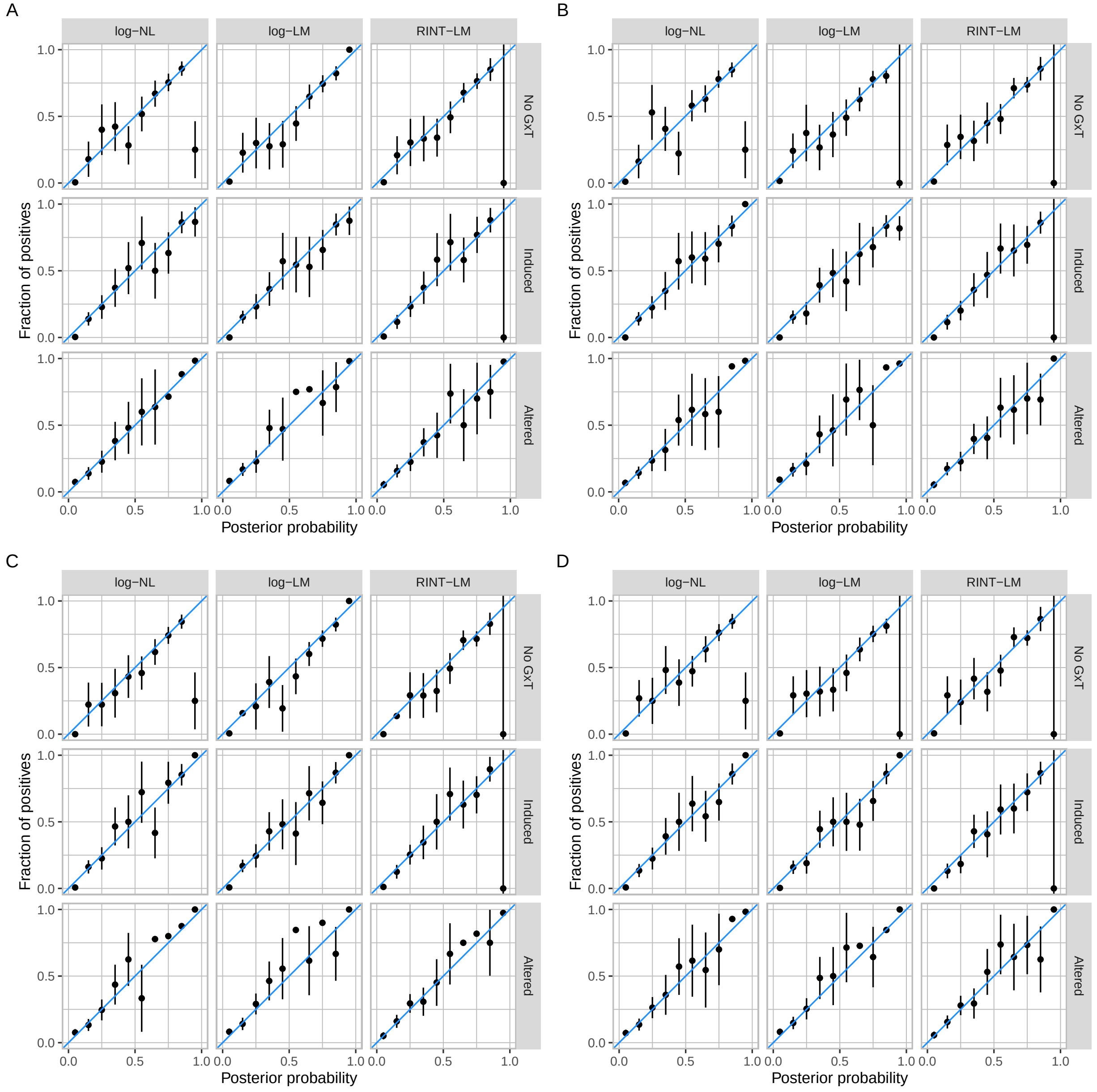

S3 Fig. Calibration of BMS with log-**NL,** log-**LM, and RINT-LM for the no-G×T, induced, and altered categories using MCMC and bridge sampling.** The x- and y-axis represent the posterior probability and the fraction of the corresponding events, respectively. The results from 800 simulations are grouped into ten equally-spaced bins. The vertical bars represent the standard errors assuming a binomial distribution. The panels **A** to **D** show the results for scenarios 1 to 4, which are defined in the legend to **S2 Fig**. See the repository (https://doi.org/10.5281/zenodo.14827827) for results of BMS using MAP estimation and Laplace approximation.
