# Supplementary material for: Probabilistic classification of gene-by-treatment interactions on molecular count phenotypes
Source: PLoS Genet. 2025 Apr 9;21(4):e1011561. doi: 10.1371/journal.pgen.1011561 (PMC12021428; doi:10.1371/journal.pgen.1011561)
Supplement: S4 Fig — (PDF) [file pgen.1011561.s004.pdf]

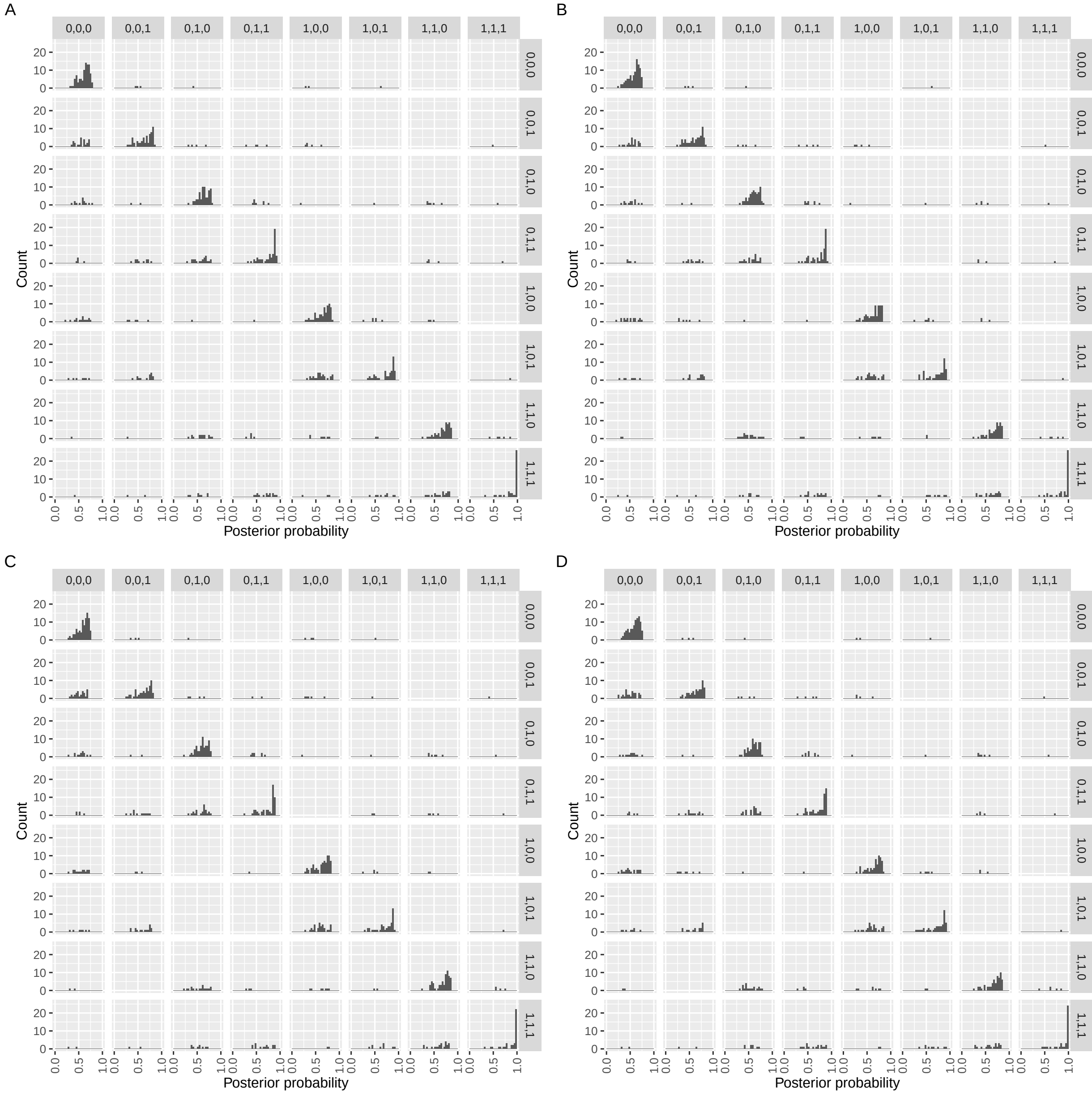

S4 Fig. Stratified histograms of posterior probability of the eight models obtained by BMS with log**-NL using MCMC and bridge sampling.** In each panel, the rows and columns represent the data-generating and posterior mode models, respectively. The panels **A** to **D** show the results for scenarios 1 to 4, which are defined in the legend to **S2 Fig**. See the repository (https://doi.org/10.5281/zenodo.14827827) for other simulation scenarios and results of BMS using MAP estimation and Laplace approximation.
