# Supplementary material for: Probabilistic classification of gene-by-treatment interactions on molecular count phenotypes
Source: PLoS Genet. 2025 Apr 9;21(4):e1011561. doi: 10.1371/journal.pgen.1011561 (PMC12021428; doi:10.1371/journal.pgen.1011561)
Supplement: S6 Fig — (PDF) [file pgen.1011561.s006.pdf]

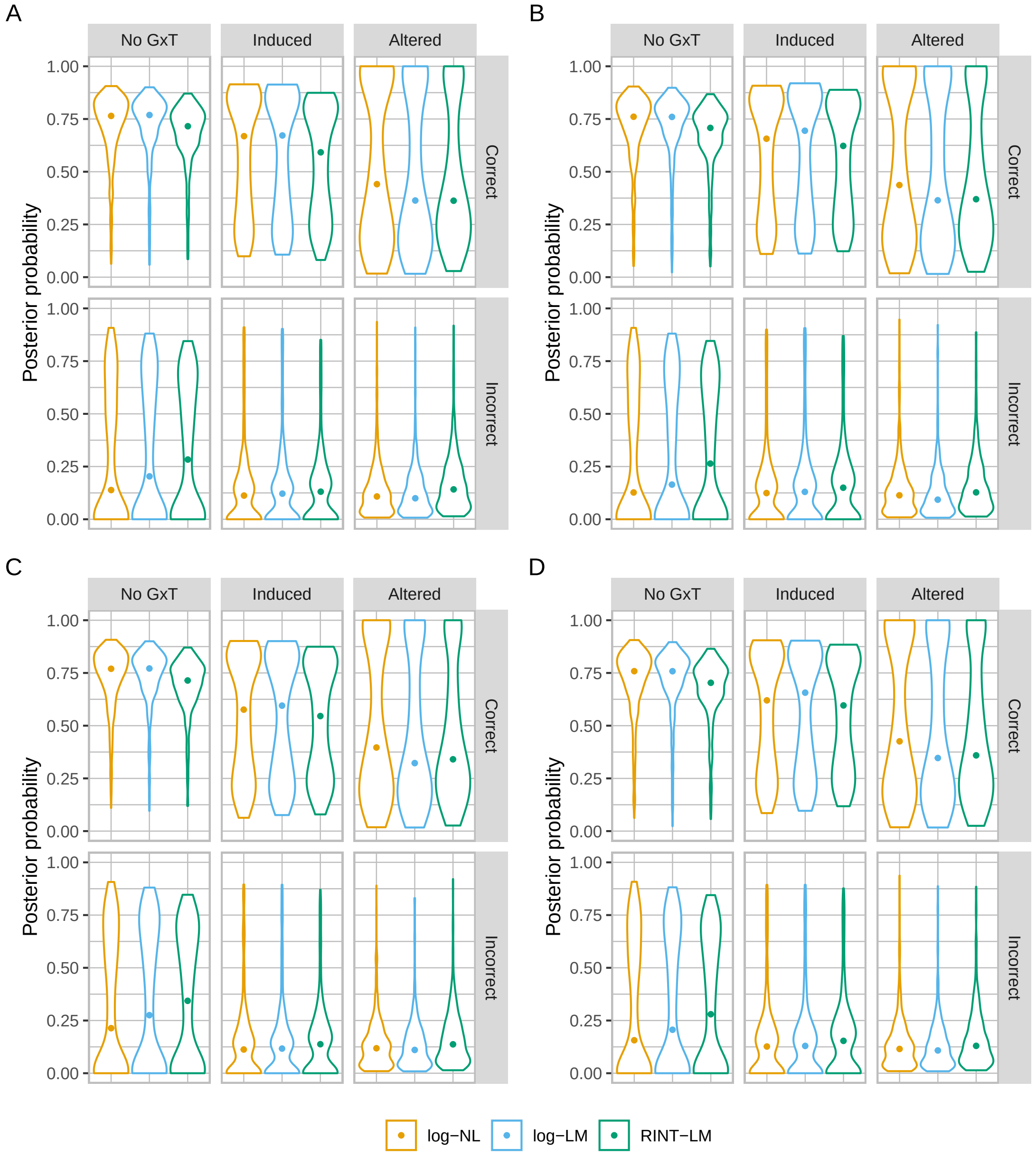

S6 Fig. Posterior probability of the correct and incorrect models for aggregated categories obtained by BMS using MCMC and bridge sampling. Violin plots for comparing the performance of BMS with log-NL, log-LM, and RINT-LM based on the distribution of posterior probability of the correct and incorrect models for the no-G×T, induced, and altered model categories. The closed circles represent median values. The panels **A** to **D** show the results for scenarios 1 to 4, which are defined in the legend to **S2 Fig**. See the repository (https://doi.org/10.5281/zenodo.14827827) for results of BMS using MAP estimation and Laplace approximation.
