# Supplementary material for: Probabilistic classification of gene-by-treatment interactions on molecular count phenotypes
Source: PLoS Genet. 2025 Apr 9;21(4):e1011561. doi: 10.1371/journal.pgen.1011561 (PMC12021428; doi:10.1371/journal.pgen.1011561)
Supplement: S7 Fig — (PDF) [file pgen.1011561.s007.pdf]

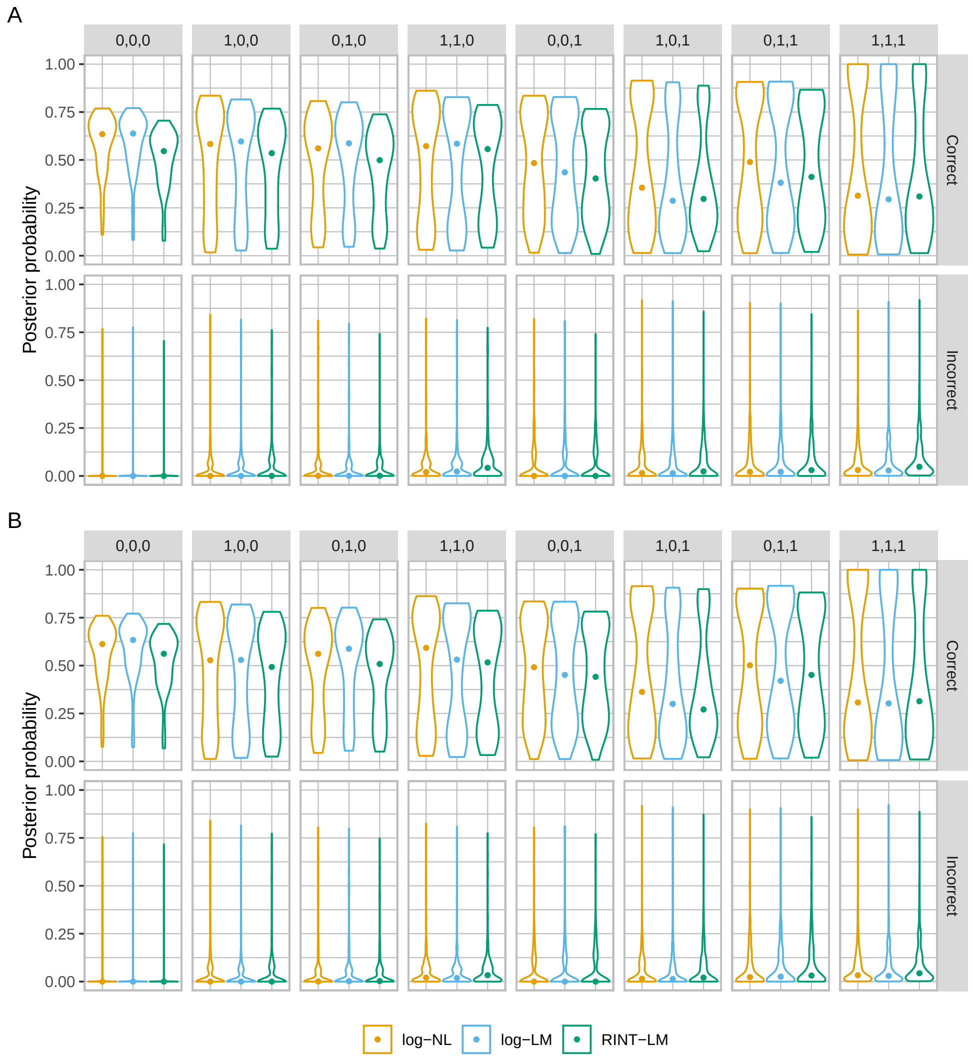

S7 Fig. Posterior probability of the correct and incorrect models for the eight categories obtained by BMS using MCMC and bridge sampling on data generated without random effect. Violin plots for comparing the performance of BMS with log-NL, log-LM, and RINT-LM based on the distribution of posterior probability of the correct and incorrect models for each of the eight model categories. The closed circles represent median values. The panels **A** and **B** respectively show the results for scenarios 1 and 2, which are defined in the legend to **S2 Fig**. See the repository (https://doi.org/10.5281/zenodo.14827827) for other simulation scenarios and results of BMS using MAP estimation and Laplace approximation.
