# Supplementary material for: Probabilistic classification of gene-by-treatment interactions on molecular count phenotypes
Source: PLoS Genet. 2025 Apr 9;21(4):e1011561. doi: 10.1371/journal.pgen.1011561 (PMC12021428; doi:10.1371/journal.pgen.1011561)
Supplement: S8 Fig — (PDF) [file pgen.1011561.s008.pdf]

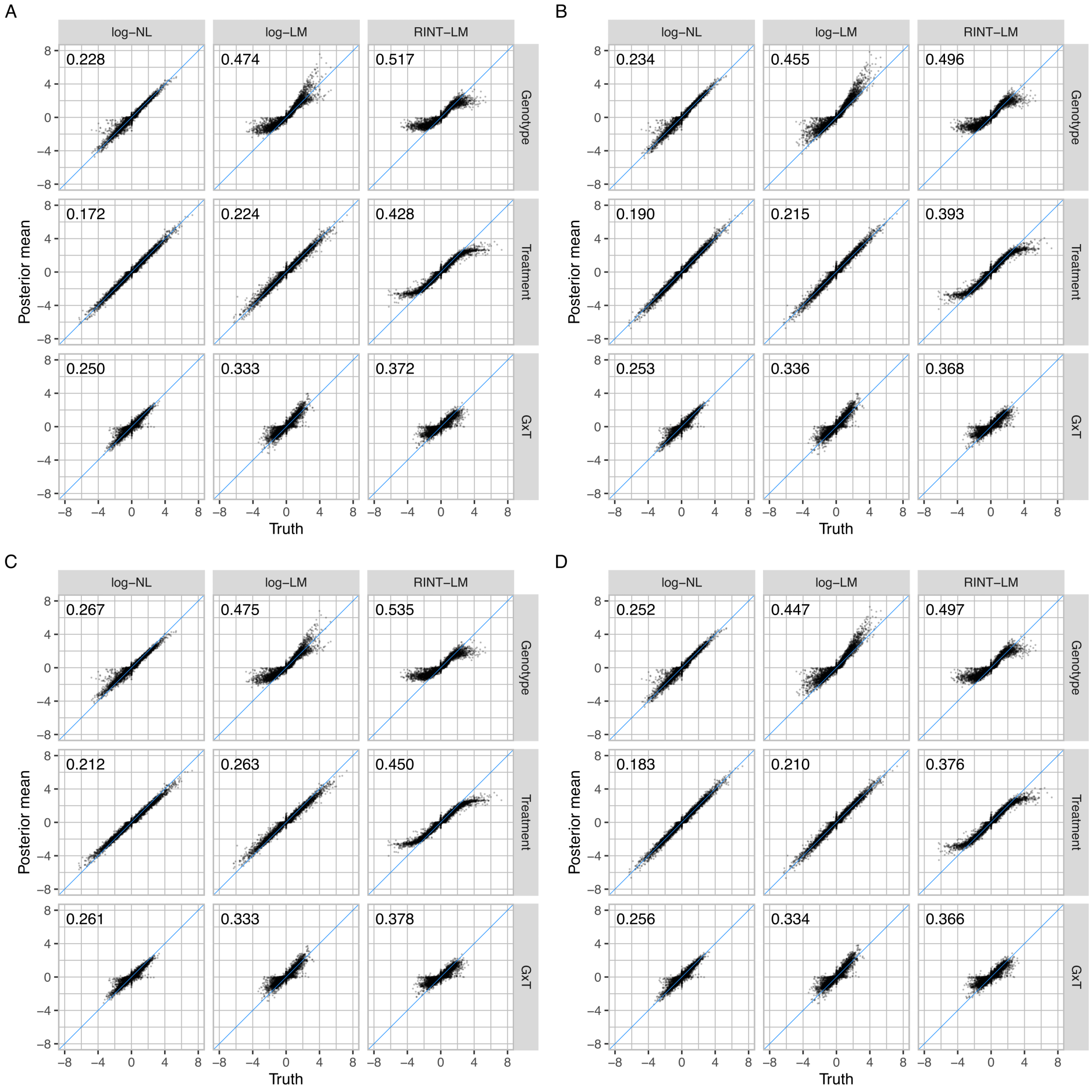


**S8 Fig. Comparison of effect estimates by Bayesian model averaging with** log-**NL,** log-**LM, and RINT-LM. A.** Scatter plots comparing estimation of the genotype, treatment, and G×T interaction effects relative to the residual standard deviation against the true values for scenario 1, which is defined in the legend to **S2 Fig**. Each point represents each of 8000 feature-SNP pairs. Shown in the top-left corner of each panel is the root mean squared error (RMSE). **B.** The same as in **A** but for scenario 2. **C.** The same as in **A** but for scenario 3. **D.** The same as in **A** but for scenario 4. See the repository (https://doi.org/10.5281/zenodo.14827827) for results obtained by MAP estimation and Laplace approximation.
