# Supplementary material for: Probabilistic classification of gene-by-treatment interactions on molecular count phenotypes
Source: PLoS Genet. 2025 Apr 9;21(4):e1011561. doi: 10.1371/journal.pgen.1011561 (PMC12021428; doi:10.1371/journal.pgen.1011561)
Supplement: S9 Fig — (PDF) [file pgen.1011561.s009.pdf]

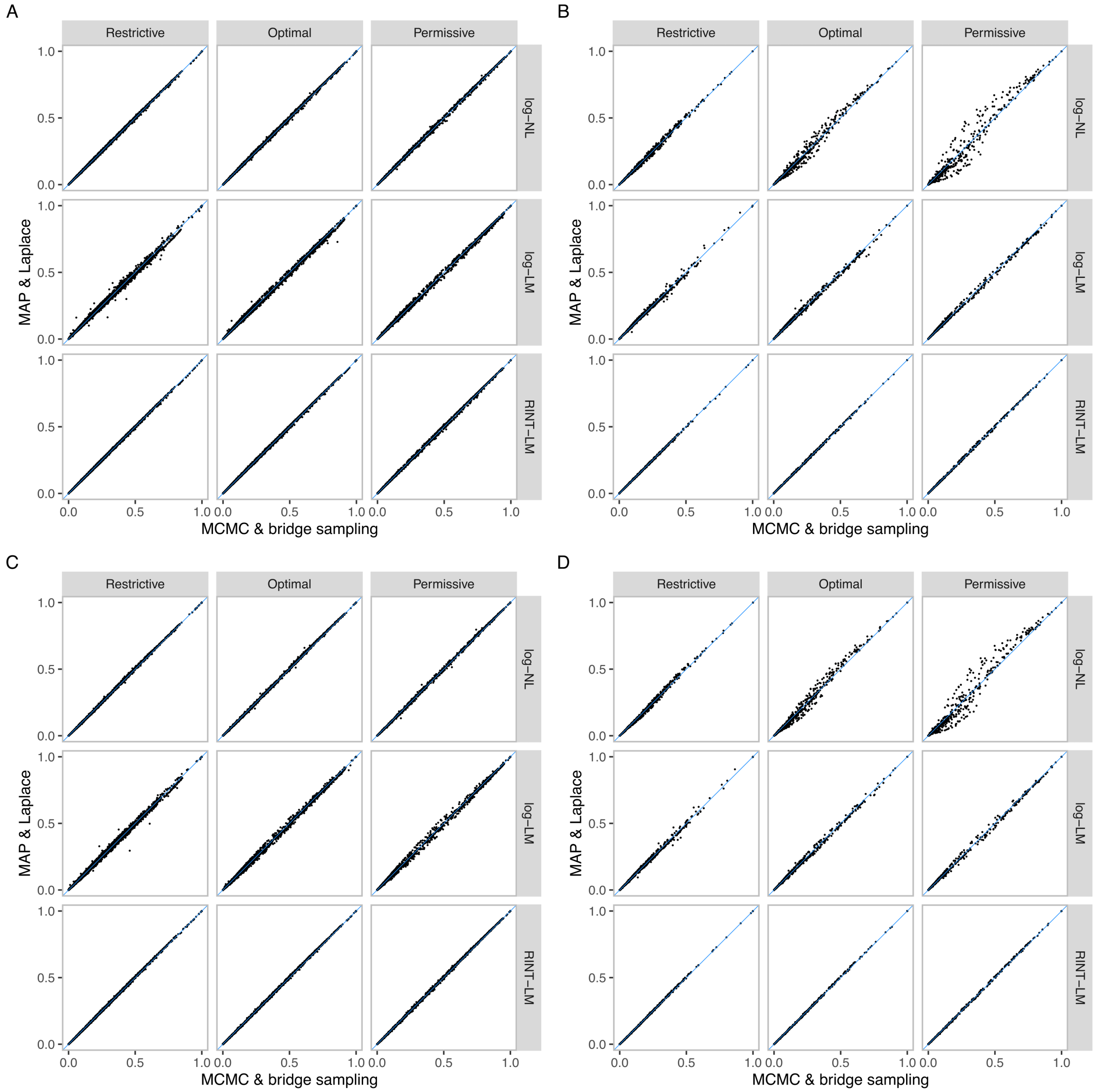

S9 Fig. Comparison of posterior probability at varying hyperparameter values between two computational approaches on synthetic data generated without donor random effect. A. Scatter plots comparing posterior probabilities obtained by MCMC followed by bridge sampling and those obtained by MAP estimation followed by Laplace approximation for scenario 1, which is defined in the legend to **S2 Fig**. Each point represents each of the eight models for a feature-SNP pair. The values are compared across eight models and 686 feature-SNP pairs for which minor allele homozygotes were present (i.e., 5488 combinations). **B.** The same as in **A** but for 114 feature-SNP pairs for which minor allele homozygotes were absent (i.e., 912 combinations). **C.** The same as in **A** but for scenario 2. **D.** The same as in **B** but for scenario 2. See the repository (https://doi.org/10.5281/zenodo.14827827) for other simulation scenarios.
