# Supplementary material for: Probabilistic classification of gene-by-treatment interactions on molecular count phenotypes
Source: PLoS Genet. 2025 Apr 9;21(4):e1011561. doi: 10.1371/journal.pgen.1011561 (PMC12021428; doi:10.1371/journal.pgen.1011561)
Supplement: S10 Fig — (PDF) [file pgen.1011561.s010.pdf]

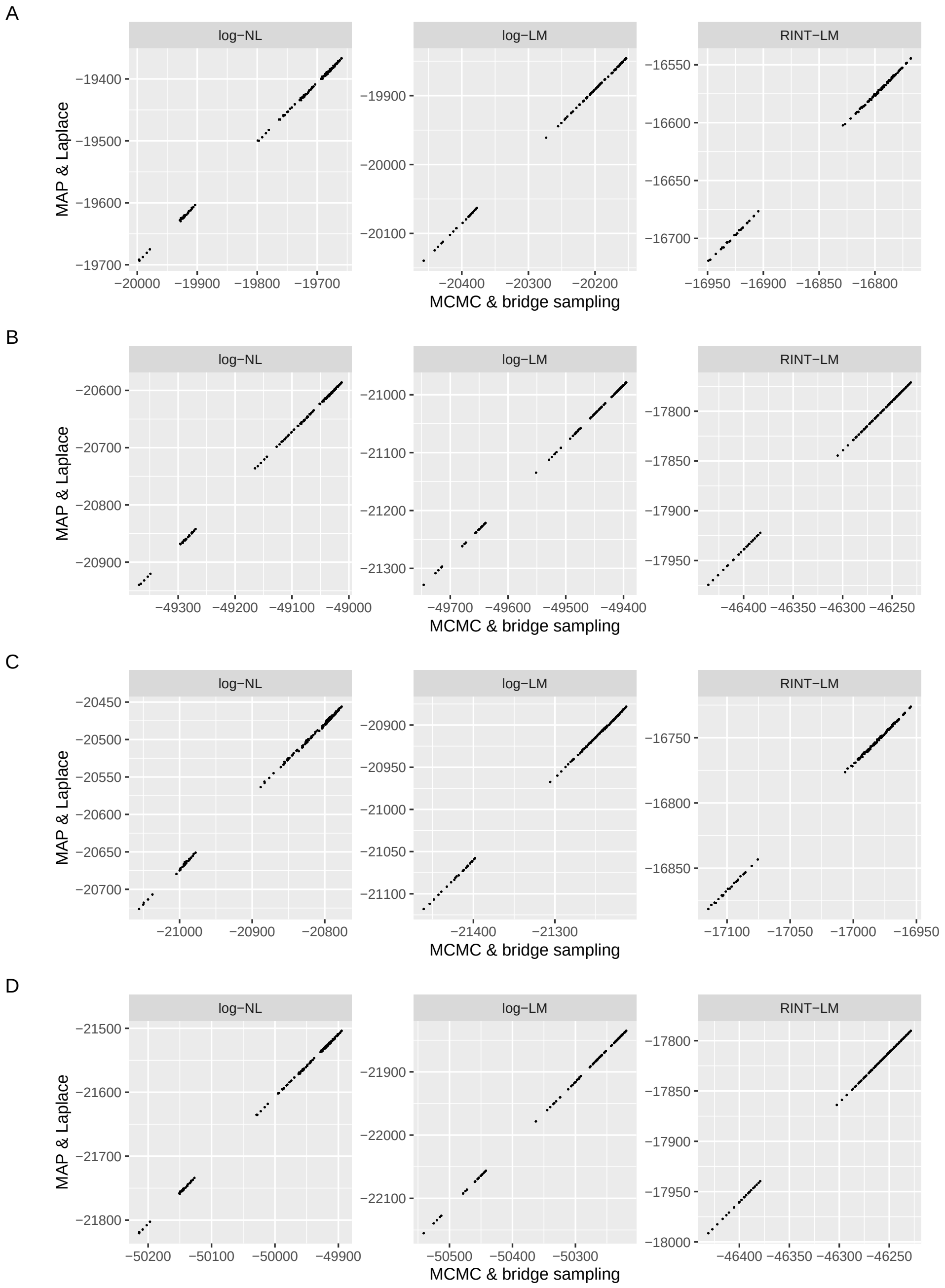

S10 Fig. Comparison of the sum of the log **of marginal likelihood across combinations of hyperparameter values between two computational approaches for** log**-NL,** log**-LM, and RINT-LM with and without donor random effects.** Scatter plots comparing results obtained by MCMC followed by bridge sampling and those obtained by MAP estimation followed by Laplace approximation. The panels **A** to **B** show the results for scenarios 1 to 4, which are defined in the legend to **S2 Fig**. Each point represents the log of the marginal likelihood summed over 80 feature-SNP pairs. The values are compared across 125 combinations of the hyperparameter values (see **S1 Text** for details).
