# Supplementary material for: Probabilistic classification of gene-by-treatment interactions on molecular count phenotypes
Source: PLoS Genet. 2025 Apr 9;21(4):e1011561. doi: 10.1371/journal.pgen.1011561 (PMC12021428; doi:10.1371/journal.pgen.1011561)
Supplement: S11 Fig — (PDF) [file pgen.1011561.s011.pdf]

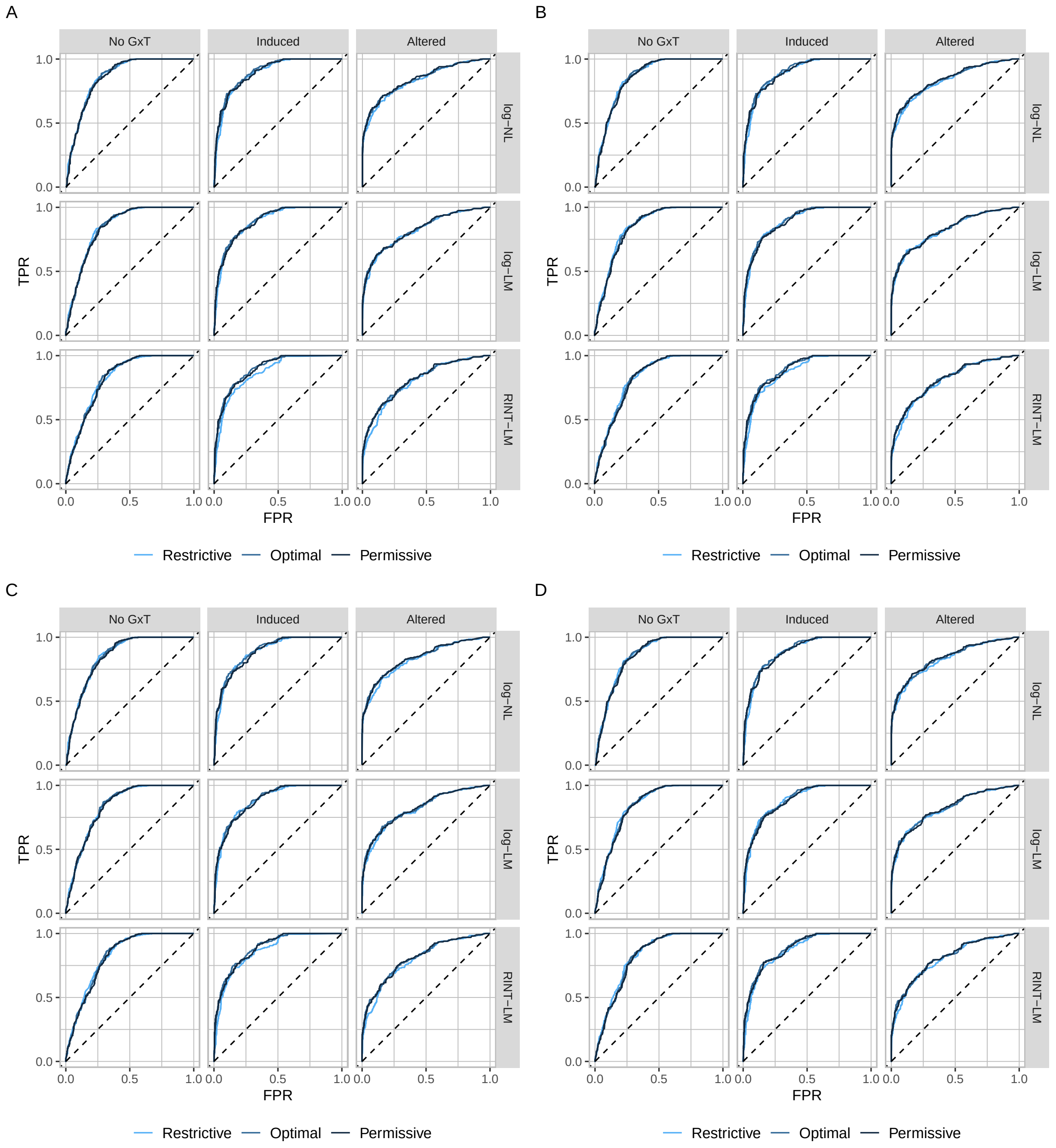

S11 Fig. ROC curves assessing the impact of the effect prior on the performance of BMS using MCMC and bridge sampling. The colors represent varying hyperparameter values. The restrictive and permissive values represent half and twice of the optimal values, respectively. The panels **A** to **D** show the results for scenarios 1 to 4, which are defined in the legend to **S2 Fig**. In each panel, the rows and columns represent modeling approaches and aggregated categories, respectively.
