# Supplementary material for: Probabilistic classification of gene-by-treatment interactions on molecular count phenotypes
Source: PLoS Genet. 2025 Apr 9;21(4):e1011561. doi: 10.1371/journal.pgen.1011561 (PMC12021428; doi:10.1371/journal.pgen.1011561)
Supplement: S12 Fig — (PDF) [file pgen.1011561.s012.pdf]

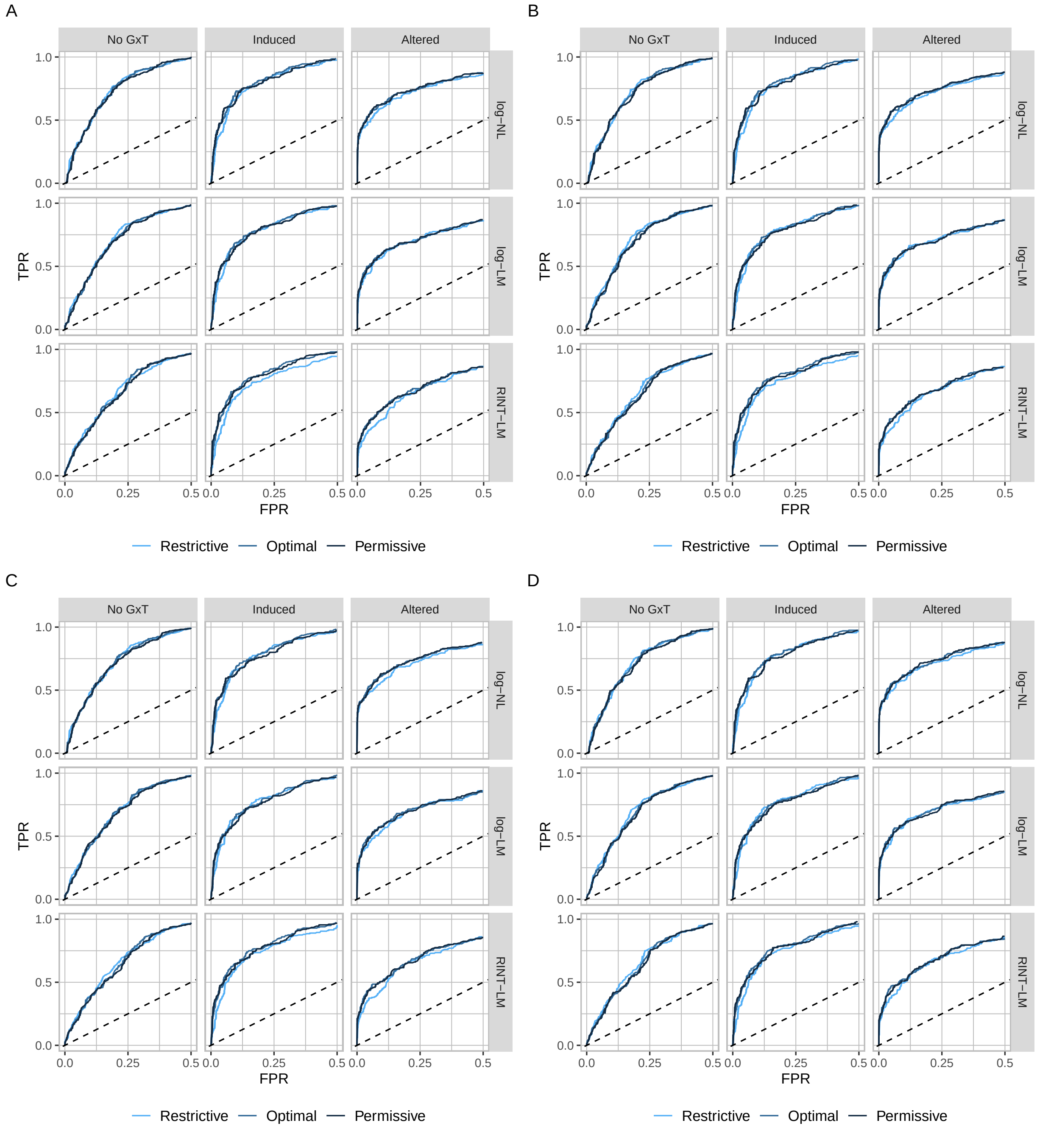

S12 Fig. Partial ROC curves assessing the impact of the effect prior on the performance of BMS using MCMC and bridge sampling. The colors represent varying hyperparameter values (see the legend to **S11 Fig**). The panels **A** to **D** show the results for scenarios 1 to 4, which are defined in the legend to **S2 Fig**. In each panel, the rows and columns represent modeling approaches and aggregated categories, respectively.
