# Supplementary material for: Probabilistic classification of gene-by-treatment interactions on molecular count phenotypes
Source: PLoS Genet. 2025 Apr 9;21(4):e1011561. doi: 10.1371/journal.pgen.1011561 (PMC12021428; doi:10.1371/journal.pgen.1011561)
Supplement: S13 Fig — (PDF) [file pgen.1011561.s013.pdf]

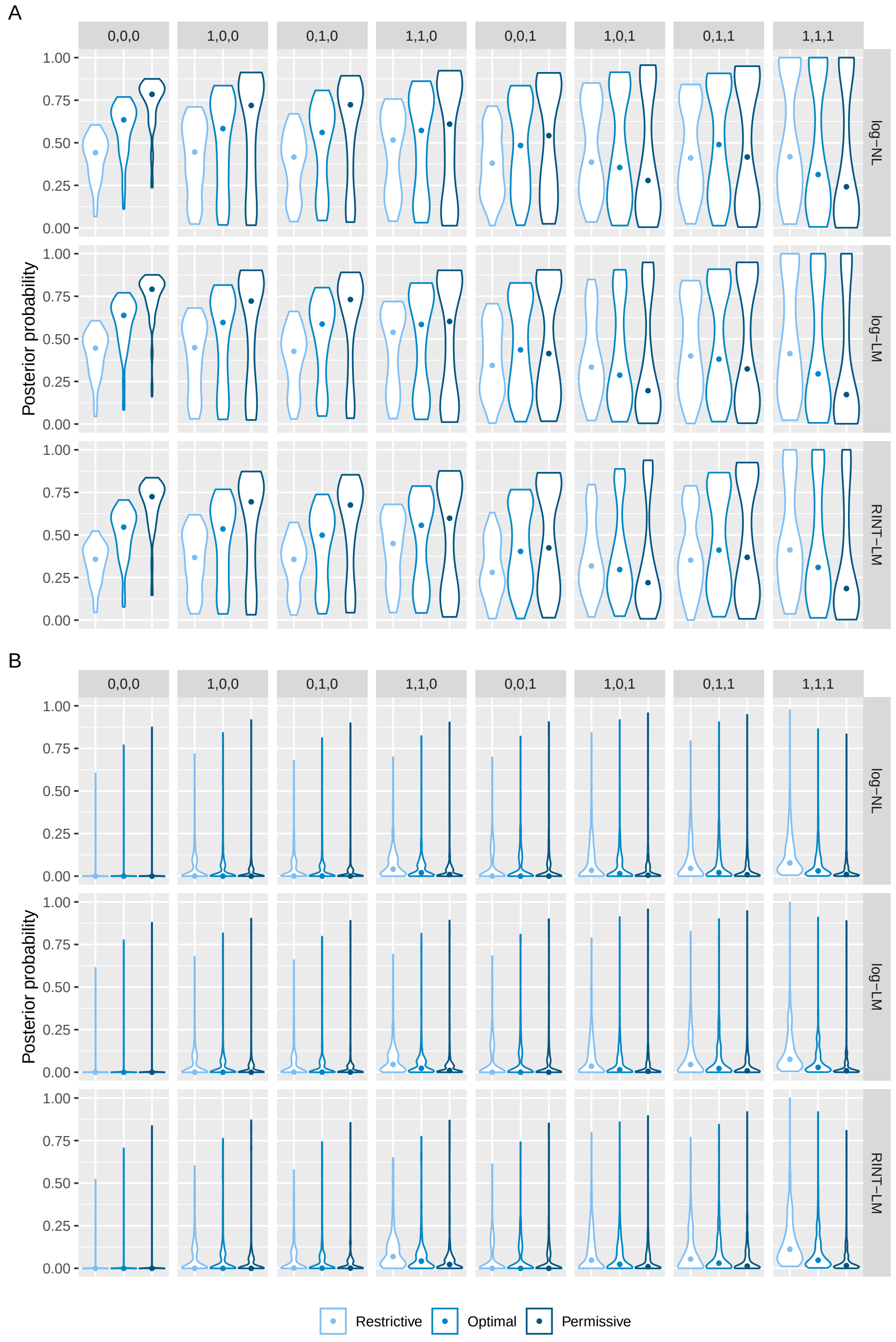

S13 Fig. Assessing the impact of the effect prior on the posterior probability of the correct and incorrect models from analyses without random effect using MCMC and bridge sampling. Violin plots showing the distribution of posterior probability of the correct (**A**) and incorrect (**B**) models for each of the eight model categories with varying hyperparameter values (see the legend to **S11 Fig**). The closed circles represent median values. Shown is the results for scenario 1, which is defined in the legend to **S2 Fig**. See the repository (https://doi.org/10.5281/zenodo.14827827) for other simulation scenarios and results of BMS using MAP estimation and Laplace approximation.
