# Supplementary material for: Probabilistic classification of gene-by-treatment interactions on molecular count phenotypes
Source: PLoS Genet. 2025 Apr 9;21(4):e1011561. doi: 10.1371/journal.pgen.1011561 (PMC12021428; doi:10.1371/journal.pgen.1011561)
Supplement: S14 Fig — (PDF) [file pgen.1011561.s014.pdf]

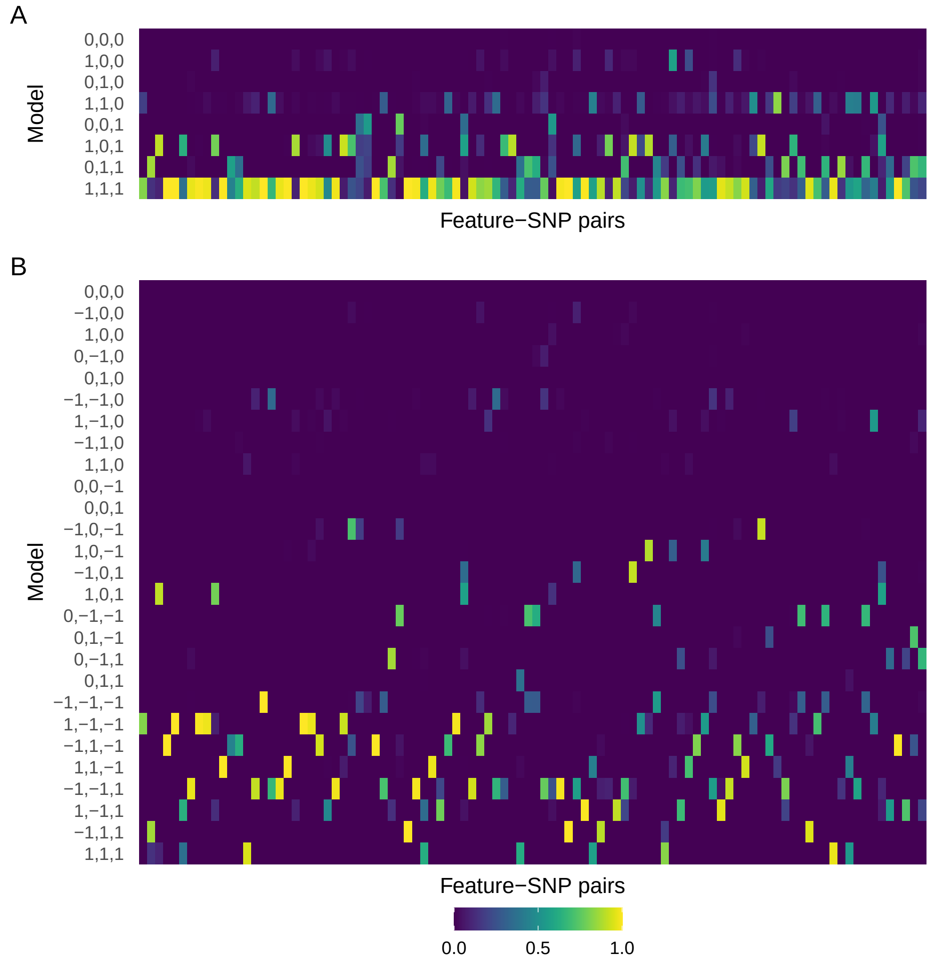

S14 Fig. Posterior probability of the models with and without accounting for the sign of effect size for the response eQTL data in hNPCs. The heatmaps show the posterior probability of the eight models for the 98 response eQTLs, which represent gene-SNP pairs with significant G×T interactions (**A**), as well as that of the 27 models accounting for the sign of effect size (**B**). The rows and columns represent the models and gene-SNP pairs, respectively. The gene-SNP pairs are ordered by P values for significant G×T interactions. The leftmost column corresponds to the smallest P value.
