# Supplementary material for: Probabilistic classification of gene-by-treatment interactions on molecular count phenotypes
Source: PLoS Genet. 2025 Apr 9;21(4):e1011561. doi: 10.1371/journal.pgen.1011561 (PMC12021428; doi:10.1371/journal.pgen.1011561)
Supplement: S15 Fig — (PDF) [file pgen.1011561.s015.pdf]

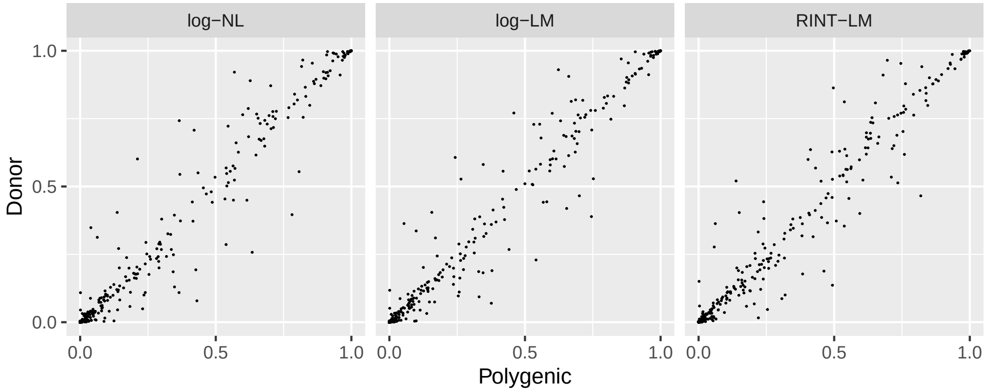

S15 Fig. Comparison of posterior probability between results with donor random effect and those with polygenic random effect for response eQTLs. Scatter plots comparing results obtained by BMS with polygenic (kinship) random effect and those with donor random effect for log-NL, log-LM, and RINT-LM. Each point represents the posterior probability of a mode for a feature-SNP pair. The values are compared across eight models and 98 feature-SNP pairs (i.e., 784 combinations).
