# Supplementary material for: Probabilistic classification of gene-by-treatment interactions on molecular count phenotypes
Source: PLoS Genet. 2025 Apr 9;21(4):e1011561. doi: 10.1371/journal.pgen.1011561 (PMC12021428; doi:10.1371/journal.pgen.1011561)
Supplement: S17 Fig — (PDF) [file pgen.1011561.s017.pdf]

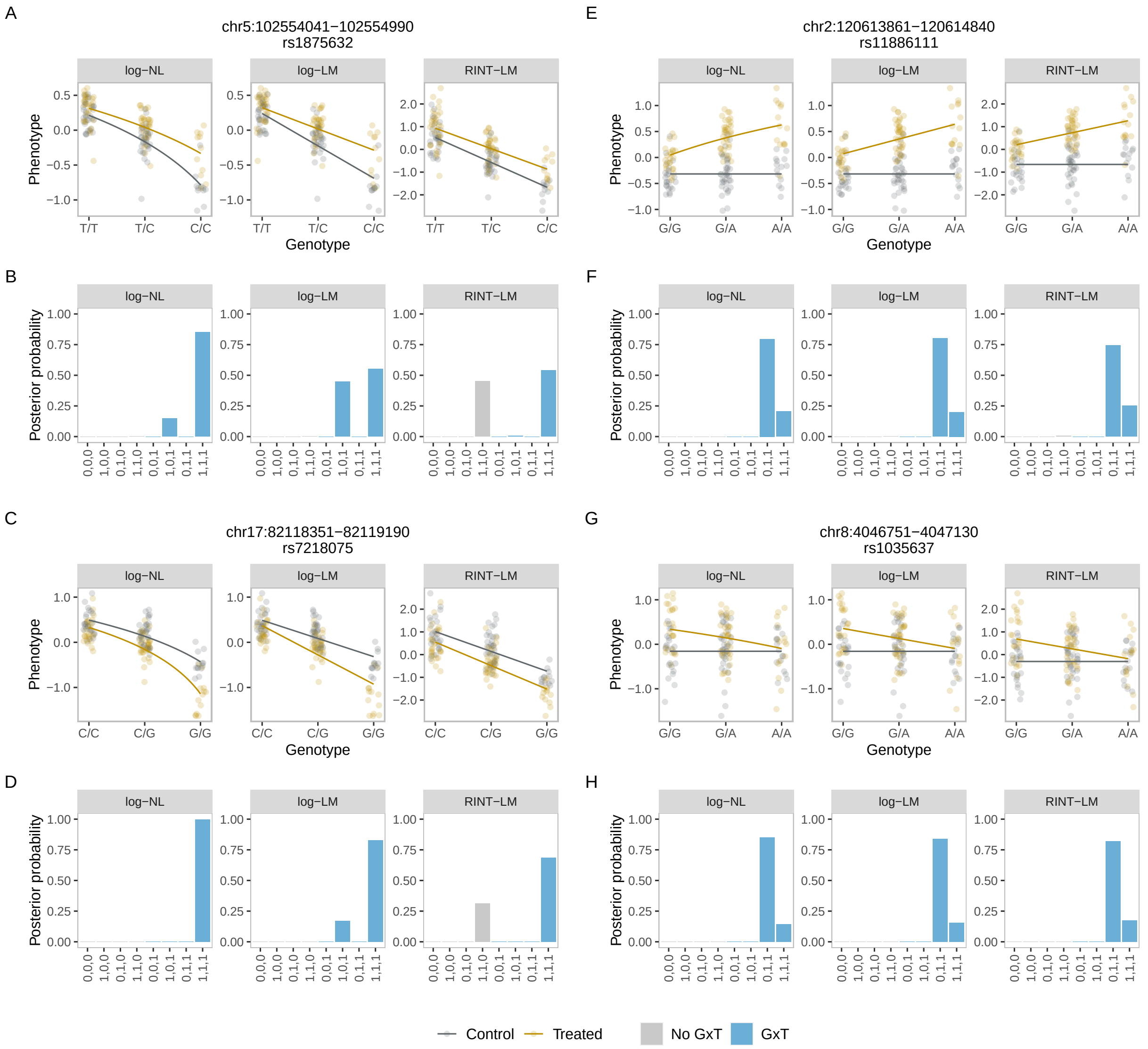

S17 Fig. Representative BMS results for the response caQTL data in hNPCs. The same as in **Fig 5** but for response caQTLs.
