# Supplementary material for: Probabilistic classification of gene-by-treatment interactions on molecular count phenotypes
Source: PLoS Genet. 2025 Apr 9;21(4):e1011561. doi: 10.1371/journal.pgen.1011561 (PMC12021428; doi:10.1371/journal.pgen.1011561)
Supplement: S18 Fig — (PDF) [file pgen.1011561.s018.pdf]

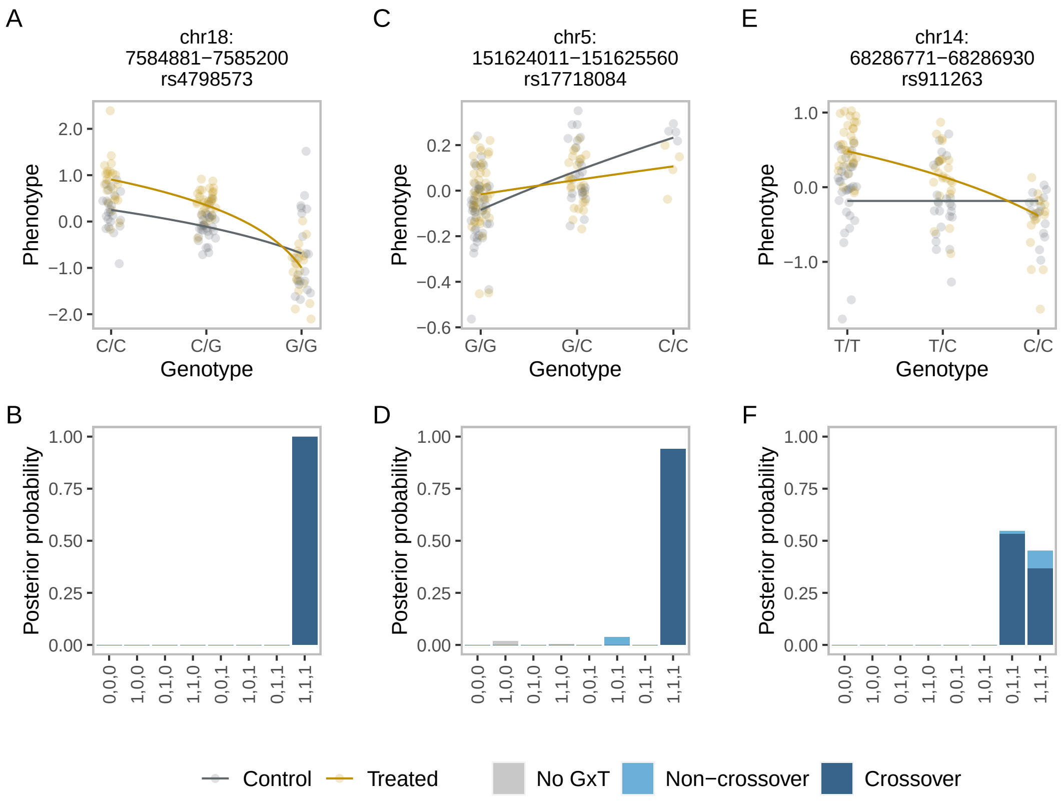

S18 Fig. Examples of response caQTLs with the crossover interaction in hNPCs. The same as in **Fig 6** but for response caQTLs.
