# Supplementary material for: Probabilistic classification of gene-by-treatment interactions on molecular count phenotypes
Source: PLoS Genet. 2025 Apr 9;21(4):e1011561. doi: 10.1371/journal.pgen.1011561 (PMC12021428; doi:10.1371/journal.pgen.1011561)
Supplement: S1 Text — (PDF) [file pgen.1011561.s019.pdf]

S1 Text

Yuriko Harigaya, Nana Matoba, Brandon D. Le, Jordan M. Valone, Jason L. Stein,
Michael I. Love†, William Valdar†
† These authors contributed equally to this work.

February 10, 2025

Contents

1 Supplementary methods **1**
1.1 The log-NL model of G×T analysis . . . 1
1.1.1 Derivation . . . 2
1.1.2 An alternative notation . . . 3
1.2 Review of previously developed methods . . . 4
1.2.1 The aFC method . . . 4
1.2.2 The ACME model . . . 4
1.3 Computing the marginal likelihood by Laplace approximation . . . 5
1.4 Hyperparameter optimization using empirical Bayes . . . 5
1.5 Including covariates . . . 6

2 Supplementary results **7**
2.1 Evaluating the Laplace approximation . . . 7
2.2 Optimizing hyperparameters . . . 8

1 Supplementary methods

1.1 The log-NL model of G×T analysis

Consider the association of a molecular phenotype, measured for a given feature, such as a gene or accessible chromatin region, with a putative *cis*-regulatory variant that has two alleles in the population, A and B, under two mutually exclusive treatment conditions. We model

$$\begin{aligned} y_i &= \log(\mu_{g,t}(g_i, t_i)) + \varepsilon_i, \\ \varepsilon_i &\overset{\text{iid}}{\sim} \mathcal{N}(0, \sigma^2), \\ \mu_{g,t}(g_i, t_i) &= (1 - \tfrac{g_i}{2})(1 - t_i)\exp(\beta_0) + (\tfrac{g_i}{2})(1 - t_i)\exp(\beta_0 + 2\beta_g) \\ &\quad + (1 - \tfrac{g_i}{2})(t_i)\exp(\beta_0 + \beta_t) + (\tfrac{g_i}{2})(t_i)\exp(\beta_0 + 2\beta_g + \beta_t + 2\beta_{g\times t}), \end{aligned} \tag{1}$$

where y_i denotes log-transformed molecular phenotype for the i-th individual ($i = 1, \cdots, n$), g_i denotes the genotype coded as $\{0, 1, 2\}$ or the imputation-based allelic dosage as $g_i \in [0, 2]$, t_i denotes an indicator variable for a treatment, β_0, β_g, β_t, and $\beta_{g\times t}$ denote regression coefficients, ε_i denotes the residual error, and σ^2 denotes the residual error variance. The $\mu_{g,t}(g, t)$ function returns a value corresponding to the phenotype in the original count scale except that, according to previous

studies ([1, 2]), we do not model a pseudocount of one, which is typically added to empirical count data. Note that, in the main text, we define $f_{g,t}(g,t) = \log(\mu_{g,t}(g,t))$. For simplicity, confounding factors are omitted.

1.1.1 Derivation

The functional form of $\mu_{g,t}(g_i, t_i)$ in Eq (1) can be derived as follows. In what follows, the subscript i is omitted for simplicity. Denote the major and minor alleles of a given SNP as A and B, respectively. Let $\mu_{AA,c}$ and $\mu_{AA,t}$ denote the phenotype values in the control and treated conditions, for the major allele homozygous respectively. Let $\mu_{BB,c}$ and $\mu_{BB,t}$ denote the phenotype values in the control and treated conditions, for the minor allele homozygous respectively. The quantities can be defined as

$$\begin{aligned}
\log(\mu_{AA,c}) &= \log(\mu_{g,t}(g=0, t=0)) = \beta_0, \\
\log(\mu_{BB,c}) &= \log(\mu_{g,t}(g=2, t=0)) = \beta_0 + 2\beta_g, \\
\log(\mu_{AA,t}) &= \log(\mu_{g,t}(g=0, t=1)) = \beta_0 + \beta_t, \\
\log(\mu_{BB,t}) &= \log(\mu_{g,t}(g=2, t=1)) = \beta_0 + 2\beta_g + \beta_t + 2\beta_{g\times t}.
\end{aligned}$$

Then, we can write

$$\begin{aligned}
\log(\mu_{AB,c}) &= \log(\mu_{g,t}(g=1, t=0)) = \log(\tfrac{1}{2}\exp(\beta_0) + \tfrac{1}{2}\exp(\beta_0 + 2\beta_g)), \\
\log(\mu_{AB,t}) &= \log(\mu_{g,t}(g=1, t=1)) = \log(\tfrac{1}{2}\exp(\beta_0 + \beta_t) + \tfrac{1}{2}\exp(\beta_0 + 2\beta_g + \beta_t + 2\beta_{g\times t})).
\end{aligned}$$

By exponentiating sides, we have

$$\mu_{AA,c} = \exp(\beta_0), \tag{2}$$
$$\mu_{BB,c} = \exp(\beta_0 + 2\beta_g), \tag{3}$$
$$\mu_{AA,t} = \exp(\beta_0 + \beta_t), \tag{4}$$
$$\mu_{BB,t} = \exp(\beta_0 + 2\beta_g + \beta_t + 2\beta_{g\times t}), \tag{5}$$

with

$$\begin{aligned}
\mu_{AB,c} &= \tfrac{\mu_{AA,c}+\mu_{BB,c}}{2}, \\
\mu_{AB,t} &= \tfrac{\mu_{AA,t}+\mu_{BB,t}}{2},
\end{aligned}$$

such that the expression values are linear with respect to the genotype. Thus, by considering the interpretation of the coefficients in a linear regression model, we have

$$\begin{aligned}
\mu_{g,t}(g,t) &= \mu_{AA,c} + \tfrac{\mu_{BB,c}-\mu_{AA,c}}{2} g + (\mu_{AA,t} - \mu_{AA,c})t + (\tfrac{\mu_{BB,t}-\mu_{AA,t}}{2} - \tfrac{\mu_{BB,c}-\mu_{AA,c}}{2})gt \qquad (6)\\
&= (1 - \tfrac{g}{2})(1-t)\mu_{AA,c} + (\tfrac{g}{2})(1-t)\mu_{BB,c} + (1-\tfrac{g}{2})(t)\mu_{AA,t} + (\tfrac{g}{2})(t)\mu_{BB,t} \\
&= (1 - \tfrac{g}{2})(1-t)\exp(\beta_0) + (\tfrac{g}{2})(1-t)\exp(\beta_0 + 2\beta_g) \\
&\quad + (1-\tfrac{g}{2})(t)\exp(\beta_0 + \beta_t) + (\tfrac{g}{2})(t)\exp(\beta_0 + 2\beta_g + \beta_t + 2\beta_{g\times t}),
\end{aligned}$$

for any $g \in [0, 2]$ and $t \in \{0, 1\}$.

1.1.2 An alternative notation

Similarly to a previous study by Mohammadi *et al.* [1], we let $\mu_{A,c} = \alpha$ denote the contribution to the phenotype value in the original count scale, $\exp(y)$, from one copy of the reference allele A in the control condition. We denote the fraction of an increase in the phenotype value per one copy of the alternative allele in the control condition by δ_g. Likewise, we denote the fraction of an increase in the phenotype value by treatment for one copy of the reference allele by δ_t. We then denote the fraction of an increase in the phenotype value per one copy of the alternative allele in the treatment condition relative to that in the control condition by $\delta_{g\times t}$. Then, the contribution from one copy of a given allele in a given condition is given by

$$\begin{aligned}\mu_{B,c} &= \alpha\delta_g,\\ \mu_{A,t} &= \alpha\delta_t,\\ \mu_{B,t} &= \alpha\delta_g\delta_t\delta_{g\times t}.\end{aligned}$$

Thus, the phenotype values can be written as

$$\begin{aligned}\mu_{AA,c} &= \mu_{A,c} + \mu_{A,c} = 2\alpha, &(7)\\ \mu_{AB,c} = \mu_{BA,c} &= \mu_{A,c} + \mu_{B,c} = (1+\delta_g)\alpha,\\ \mu_{BB,c} &= \mu_{B,c} + \mu_{B,c} = 2\alpha\delta_g, &(8)\\ \mu_{AA,t} &= \mu_{A,t} + \mu_{A,t} = 2\alpha\delta_t, &(9)\\ \mu_{AB,t} = \mu_{BA,t} &= \mu_{A,t} + \mu_{B,t} = (1+\delta_g\delta_{g\times t})\alpha\delta_t,\\ \mu_{BB,t} &= \mu_{B,t} + \mu_{B,t} = 2\alpha\delta_g\delta_t\delta_{g\times t}, &(10)\end{aligned}$$

based on the assumption of allelic additivity. Substituting Eq (7), (8), (9), and (10) for $\mu_{A,c}$, $\mu_{A,t}$, $\mu_{B,c}$, and $\mu_{B,t}$, respectively, in Eq (6), we have

$$\mu_{g,t} = 2\alpha + \alpha(\delta_g - 1)g + 2\alpha(\delta_t - 1)t + \alpha(\delta_g\delta_t\delta_{g\times t} - \delta_g - \delta_t + 1)gt, \tag{11}$$

for any $g \in [0, 2]$ and $t \in \{0, 1\}$. From a comparison of Eq (2), (3), (4), (5), (6), and (11), we have

$$\begin{aligned}\alpha &= \frac{1}{2}\exp(\beta_0),\\ \delta_g &= \exp(2\beta_g),\\ \delta_t &= \exp(\beta_t),\\ \delta_{g\times t} &= \exp(2\beta_{g\times t}).\end{aligned}$$

The eight models can be obtained as

$$\mu_{g,t} = \begin{cases} 2\alpha, & \text{if } \delta_g = 1,\ \delta_t = 1,\ \delta_{g\times t} = 1\\ 2\alpha + \alpha(\delta_g - 1)g, & \text{if } \delta_g \neq 1,\ \delta_t = 1,\ \delta_{g\times t} = 1\\ 2\alpha + 2\alpha(\delta_t - 1)t, & \text{if } \delta_g = 1,\ \delta_t \neq 1,\ \delta_{g\times t} = 1\\ 2\alpha + 2\alpha(\delta_g - 1)g + 2\alpha(\delta_t - 1)t + \alpha(\delta_g\delta_t - \delta_g - \delta_t + 1)gt, & \text{if } \delta_g \neq 1,\ \delta_t \neq 1,\ \delta_{g\times t} = 1\\ 2\alpha + \alpha(\delta_{g\times t} - 1)gt, & \text{if } \delta_g = 1,\ \delta_t = 1,\ \delta_{g\times t} \neq 1\\ 2\alpha + \alpha(\delta_g - 1)g + \alpha\delta_g(\delta_{g\times t} - 1)gt, & \text{if } \delta_g \neq 1,\ \delta_t = 1,\ \delta_{g\times t} \neq 1\\ 2\alpha + 2\alpha(\delta_t - 1)t + \alpha\delta_t(\delta_{g\times t} - 1)gt, & \text{if } \delta_g = 1,\ \delta_t \neq 1,\ \delta_{g\times t} \neq 1\\ 2\alpha + \alpha(\delta_g - 1)g + 2\alpha(\delta_t - 1)t + \alpha(\delta_g\delta_t\delta_{g\times t} - \delta_g - \delta_t + 1)gt, & \text{if } \delta_g \neq 1,\ \delta_t \neq 1,\ \delta_{g\times t} \neq 1. \end{cases}$$

Moreover, in the control and treated conditions ($t = 0$ and $t = 1$), we respectively have

$$\mu_g = 2\alpha + \alpha(\delta_g - 1)g$$

and

$$\mu_g = 2\alpha\delta_t + \alpha\delta_t(\delta_g\delta_{g\times t} - 1)g.$$

Here, α and $\alpha\delta_t$ correspond to the phenotype values for the major allele homozygous donors in the control and treated conditions, respectively. δ_g and $\delta_g\delta_{g\times t}$ correspond to the allelic fold changes (the fraction of an increase in the phenotype value per one copy of the alternative allele) in the control and treated conditions, respectively. Thus, $\delta_{g\times t} = 1$ ($\beta_{g\times t} = 0$) corresponds to a situation where the allelic fold changes are identical between the two conditions. Note that, if $\delta_t = \delta_{g\times t} = 1$, this model reduces to the nonlinear model for estimating the aFC value (1.2.1) and the ACME model (1.2.2). The α parameter corresponds to e_0 and $\frac{1}{2}\beta_0$ in the aFC and ACME models, respectively. The δ_g parameter corresponds to $\exp(\log_2(\delta_{0,1}))$ and $2\eta + 1$ in the aFC and ACME models, respectively.

1.2 Review of previously developed methods

In this section, we review previously identified methods for single-condition molecular QTL mapping based on the allelic additivity assumption. Note that we use notations by the authors, which are not consistent with those in other sections or the main text.

1.2.1 The aFC method

Mohammadi *et al.* [1] proposed a use of allelic fold change (aFC), $\delta_{0,1}$, for quantifying the effect size of *cis*-eQTLs. In the study, they described a model to estimate aFC from eQTL data with multiplicative noise, which is closely related to our approach. The model is cast as

$$y_i = \{(2 - t_n) - t_n\delta_{1,0}\}e_0\,\varepsilon_n, \tag{12}$$

where y_n denotes the gene expression in the original count scale for the n-th individual, t_n denotes the number of alternative alleles coded as $\{0, 1, 2\}$, e_0 is the expression per copy of the reference allele, and ε_n denotes a noise such that $\log_2(\varepsilon_n)$ is normally distributed with null mean and unknown variance. Taking the logarithm of the both-hand sides of Eq (12) yields

$$\log_2(y_n) = \log_2\{(2 - t_n) + t_n\delta_{1,0}\} + \log_2 e_0 + \log_2 \varepsilon_n.$$

Note that confounding factors are omitted for the ease of exposition.

1.2.2 The ACME model

Palowitch *et al.* [2] proposed the Additive Contribution on the original expression scale with Multiplicative Error (ACME) model. The model is cast as

$$\begin{aligned} y_i &= \log(\beta_0 + \beta_1 s_i) + \mathbf{Z}_i^T\boldsymbol{\gamma} + \varepsilon_i, \\ \varepsilon_i &\overset{\text{iid}}{\sim} \mathcal{N}(0, \sigma^2), \end{aligned} \tag{13}$$

where y_i denotes the log-transformed read count for the i-th individual ($i = 1, \cdots, n$), s_i denotes the genotype (the minor allele count) coded as $\{0, 1, 2\}$, β_0 is the baseline mean expression, β_1 is the additive contribution of each allele, $\mathbf{Z}_i \in \mathbb{R}^{p\times 1}$ denotes a vector of covariates, $\boldsymbol{\gamma} \in \mathbb{R}^{p\times 1}$ denotes

corresponding coefficients, ε_i denotes independent residual errors, and σ^2 denotes the residual error variance. Eq (13) can be rewritten as

$$\begin{aligned} y_i &= \log(\beta_0) + \log\left(1 + \frac{\beta_1}{\beta_0}\right) + \mathbf{Z}_i\boldsymbol{\gamma} + \varepsilon_i, \\ &= \log(\beta_0) + \log(1+\eta) + \mathbf{Z}_i\boldsymbol{\gamma} + \varepsilon_i, \end{aligned}$$

where β and η determine the baseline expression and the effect of genotype, respectively.

1.3 Computing the marginal likelihood by Laplace approximation

The marginal likelihood of $\mathbf{y} = \{y_i\}_{i=1}^n$ given model $\mathbf{m}$ is

$$p(\mathbf{y} \mid \mathbf{m}) = \int p(\mathbf{y}|\boldsymbol{\theta}, \mathbf{m})p(\boldsymbol{\theta}|\mathbf{m})\,\mathrm{d}\boldsymbol{\theta}\,,$$

where $\boldsymbol{\theta} \in \mathbb{R}^p$ is the parameter vector of length $p \in \{2, \ldots, 5\}$ that comprises β_0, σ^2, and some, all, or none of β_g, β_t, and $\beta_{g\times t}$, as determined by the model $\mathbf{m}$, and $p(\mathbf{y}|\boldsymbol{\theta}, \mathbf{m})$ and $p(\boldsymbol{\theta}|\mathbf{m})$ are the corresponding likelihood and prior. To approximate the marginal likelihood deterministically, we use the Laplace approximation, namely,

$$\int \exp(-nh(\boldsymbol{\theta}))\,\mathrm{d}\boldsymbol{\theta} \approx \exp\left(-nh(\hat{\boldsymbol{\theta}})\right)(2\pi)^{\frac{p}{2}}|\boldsymbol{\Sigma}|^{\frac{1}{2}}n^{-\frac{p}{2}}\,,$$

where $h(\boldsymbol{\theta})$ is a function of the (unnormalized) log posterior, namely,

$$h(\boldsymbol{\theta}) = -\frac{1}{n}\log p(\mathbf{y}|\boldsymbol{\theta}, \mathbf{m}) - \frac{1}{n}\log p(\boldsymbol{\theta}|\mathbf{m})\,,$$

and where $\hat{\boldsymbol{\theta}}$ the value of $\boldsymbol{\theta}$ that maximizes $-h(\boldsymbol{\theta})$, i.e., the *maximum a posteriori* (MAP) estimate of $\boldsymbol{\theta}$, and $\boldsymbol{\Sigma} \in \mathbb{R}^{p\times p}$ is the inverse negative of the Hessian matrix evaluated at the MAP. The MAP estimate and corresponding approximate Hessian are obtained numerically using the BFGS algorithm implemented in the function `optim()` in the R package "stats." This calculation of the marginal likelihood via Laplace approximation is performed for each of the eight models.

1.4 Hyperparameter optimization using empirical Bayes

In BMS, properly optimizing the prior hyperparameters can improve inference. For the optimization, we undertook an empirical Bayes approach, where we used a grid search to obtain the values of $\boldsymbol{\phi}$ that maximize the sum of the log of marginal likelihood across all feature-SNP pairs under consideration. Note that $\boldsymbol{\phi} = (\phi_g, \phi_t, \phi_{g\times t})$ control the effect sizes relative to the residual error standard deviation. In this approach, the sum of the log of marginal likelihood is maximized over a grid of candidate hyperparameter values. For simulation analysis, optimal values were searched over a 3-dimensional grid spanning values from 0.25 to 2.5 in 0.25 increments. For analysis of the experimental data, searches were performed over a 3-dimensional grid spanning values from 0.25 to 3.0 in 0.25 increments. For computational efficiency, we use MAP estimation via optimization followed by Laplace approximation rather than MCMC followed by bridge sampling. In principle, the sum of the maximum likelihood across all candidate feature-SNP pairs in the data needs to be maximized. For computational efficiency, however, we estimate hyperparameter values only from the pre-selected set of response molecular QTLs that are being categorized. This choice can be

justified as follows. Assume that, for a given feature-SNP pair, the "correct" model is $\mathbf{m} = (1, 1, 0)$. Then, consider the marginal likelihood

$$\begin{aligned} p(\mathbf{y}) &= \sum_{\mathbf{m} \in \mathcal{M}} p(\mathbf{y} \mid \mathbf{m}) \\ &= p(\mathbf{y} \mid \mathbf{m} = (1,1,0)) \Pr(\mathbf{m} = (1,1,0)) + \sum_{\mathbf{m} \neq (1,1,0)} p(\mathbf{y} \mid \mathbf{m}) \Pr(\mathbf{m}). \end{aligned}$$

In the last line, the first term has a large $p(\mathbf{y} \mid \mathbf{m})$ value and does not contain $\phi_{g\times t}$. The second term is small since all other $p(\mathbf{y} \mid \mathbf{m})$ values are small. Thus, $p(\mathbf{y})$ does not substantially depend on $\phi_{g\times t}$. That is, this pair does not substantially affect the functional relationship between the marginal likelihood and $\phi_{g\times t}$. If the "correct" model is $\mathbf{m} = (0, 0, 0)$, none of the $\boldsymbol{\phi}$ elements substantially impact the marginal likelihood. Thus, it is reasonable to focus on feature-SNP pairs with significant associations to estimate optimal values of $\boldsymbol{\phi}$. We also note that, for the accuracy of model selection, hyperparameter values only need to be in an appropriate scale but do not need to be exactly maximizing the likelihood of the data. The hyperparameter optimization results are described in **Optimizing hyperparameters**.

1.5 Including covariates

In analysis of experimental data, it is crucial to control for confounding factors to avoid spurious inference. To specify the model that includes confounding factors as fixed or random effects, we let r denote the number of donors and n_r denote the number of samples in the r-th donor group. That is, $n = \sum_{r=1}^{R} n_r$. Then, for the i-th sample in the r-th donor group ($i = 1, \ldots, n_r$, $r = 1, \ldots, R$), a G×T interaction model can be cast as

$$\begin{aligned} y_{i,r} &= f_{g,t}(g_{i,r}, t_{i,r}) + \mathbf{x}_{i,r}^T \boldsymbol{\gamma} + u_r + \varepsilon_{i,r}, \\ \mathbf{u} &\sim \mathcal{N}_R(0, \sigma_u^2 \mathbf{K}), \\ \varepsilon_{i,r} &\overset{\text{iid}}{\sim} \mathcal{N}(0, \sigma^2), \end{aligned}$$

where $y_{i,r}$ denotes the phenotype, $f_{g,t}(\cdot,\cdot)$ is defined as in Eq (11) in the main text, $\mathbf{x}_{i,r} \in \mathbb{R}^q$ denotes a vector of fixed-effect factors, $\boldsymbol{\gamma} \in \mathbb{R}^q$ denotes a vector of the corresponding coefficient, $\mathbf{u} \in \mathbb{R}^R$ denotes a vector of random effects, σ_u denotes the random effect standard deviation, $\varepsilon_{i,r}$ denotes the residual error, and σ^2 denotes the residual error variance. The matrix $\mathbf{K}$ denotes a kernel matrix and can be a known kinship matrix, which represents genetic relatedness between donors. Alternatively, when the genetic relatedness is included as a fixed effect, $\mathbf{K}$ can be set to the R-dimensional identity matrix $\mathbf{I}_R$. In the matrix form, we have

$$\mathbf{y} \sim \mathcal{N}_n(f_{g,t}(\mathbf{g}, \mathbf{t}) + \mathbf{X}\boldsymbol{\gamma} + \mathbf{Z}\mathbf{u}, \sigma^2 \mathbf{I}),$$

where $\mathbf{y} \in \mathbb{R}^n$ denotes the outcome vector,

$$\begin{aligned} f_{g,t}(\mathbf{g}, \mathbf{t}) = (&f_{g,t}(g_{1,1}, t_{1,1}), \ldots, f_{g,t}(g_{n_1,1}, t_{n_1,1}), \\ &\ldots, \\ &f_{g,t}(g_{1,R}, t_{1,R}), \ldots, f_{g,t}(g_{n_R,R}, t_{n_R,R}))^T \in \mathbb{R}^n \end{aligned}$$

denotes the mean values, $\mathbf{X} \in \mathbb{R}^{n\times q}$ denotes a matrix of fixed-effect factors, and $\mathbf{Z} \in \mathbb{R}^{n\times R}$ denotes an incidence matrix indicating the group membership of the samples, Since the goal is to estimate the parameters of the $f_{g,t}(\cdot)$ function, $\boldsymbol{\beta}$, we marginalize out the random effects $\mathbf{u}$ and obtain

$$\mathbf{y} \sim \mathcal{N}_n(f_{g,t}(\mathbf{g}, \mathbf{t}) + \mathbf{X}\boldsymbol{\gamma}, \sigma_u^2 \mathbf{Z}\mathbf{K}\mathbf{Z}^T + \sigma^2 \mathbf{I}).$$

Then, the parameters $\boldsymbol{\beta}$, $\boldsymbol{\gamma}$, σ_u, and σ can be estimated simultaneously by a frequentists or a Bayesian approach.

In our G×T analysis of the gene expression and chromatin accessibility data from hNPCs, we included top 10 principal components (PCs) of the molecular count data as fixed effects and the genetic relatedness (kinship) among the donors as a random effect as in the previous study [3]. The kinship matrix was obtained using the R package "GENESIS" [4]. The molecular count PCs were identified in two steps. First, for each feature, we combined the library size-scaled, log-transformed molecular count data in the control and treated conditions and residualized the combined data with respect to the treatment indicator variable. We did not regress out the donor or kinship random effect since the effect is unlikely to be captured in the top PCs. Second, we obtained PCs by performing principal component analysis (PCA) on the residuals. Then residualized phenotype data, y_i, was obtained by regressing out the top 10 PCs from the library size-scaled, log-transformed data before the first step. We included 22354 genes and 172887 ATAC-peak regions as features. Although it is ideal to control for the factors simultaneously with examining the associations of the phenotype with the genotype and treatment, such model formulation can pose computational challenges due to a large number of parameters to be estimated. Therefore, we regressed out the library-size scaled, log-transformed count data with respect to the fixed-effect confounding factors and fit models including the random effect only. Note that we did not consider fixed-effect confounding factors in the simulation experiments.

For the single-condition analysis for assessing the allelic additivity, we modeled

$$\begin{aligned} y_i &= f_g(g_i) + \mathbf{x}_i^T \boldsymbol{\gamma} + \varepsilon_i, \\ \varepsilon_i &\overset{\text{iid}}{\sim} \mathcal{N}(0, \sigma^2), \end{aligned}$$

where y_i, g_i, σ^2, and ε_i are defined as in Eq (2) and (3) in the main text, $f_g(\cdot)$ is defined as in Eq (4) in the main text, $\mathbf{x}_i \in \mathbb{R}^q$ is a vector of fixed-effect factors for the i-th sample, and $\boldsymbol{\gamma} \in \mathbb{R}^q$ is a vector of the corresponding coefficient. As fixed-effect factors, we included top 10 PCs, which we obtained by performing PCA separately for each condition.

2 Supplementary results

2.1 Evaluating the Laplace approximation

Although MCMC followed by bridge sampling [5] provides accurate approximations with sufficient sampling [6], this approach is computationally intensive and is prohibitive for optimizing hyperparameters. MAP estimation followed by Laplace approximation [7, 8] is magnitudes of order faster and suited for a large number of model fitting routines. Since error bounds for Laplace approximation are not easily obtained [7], we empirically assessed its performance by comparing simulation results obtained by MAP estimation followed by Laplace approximation with those obtained by MCMC followed by bridge sampling.

To examine whether MAP estimation followed by Laplace approximation is adequate for optimizing hyperparameters for the effect priors, we compared the sum of log-marginal likelihood over 80 feature-SNP pairs (i.e., 10 instances per data-generating model) across 125 combinations of hyperparameter values, corresponding to a grid spanning from 0.5 to 2.5 by 0.5. We note that, out of the 80 feature-SNP pairs, 14 lacked samples from the minor allele homozygote, that is, only

contained samples at $g = 0$ and $g = 1$. Such instances can occur when the minor allele frequency is low and can occasionally cause computational issues as we discuss below. Nonetheless, we observed that the sum of log-marginal likelihood was nearly identical up to normalization constants across the hyperparameter combinations (S10 Fig). We note that our implementation of Laplace approximation only gives relative log-marginal likelihood values, which suffices for our purposes. The observation can justify the use of MAP estimation and Laplace approximation for optimizing the hyperparameter values based on the sum of log-marginal likelihood across feature-SNP pairs.

To examine the adequacy of MAP estimation followed by Laplace approximation for posterior inference, we compared the posterior probability across the eight models for 800 feature-SNP pairs (i.e., 100 instances per data-generating model) with varying hyperparameter values. Out of the 800 feature-SNP pairs, 114 lacked samples from the minor allele homozygote, that is, only contained samples at $g = 0$ and $g = 1$. Given the suggested adequacy of the computational method for hyperparameter optimization, we obtained the optimal values using MAP estimation followed by Laplace approximation. We then performed MCMC followed by bridge sampling with the optimal hyperparameter values as well as half and twice the values, which are restrictive and permissive, respectively. The analyses led to the following points. First, for feature-SNP pairs with at least one sample from the minor allele homozygote, the posterior probability of the eight models based on log-NL obtained by the two approaches were nearly identical (S9A and S9C Fig) [9]. Second, for feature-SNP pairs lacking minor allele homozygotes, however, the posterior probabilities of the eight models based on log-NL obtained by Laplace approximation deviated from the values obtained by MCMC followed by bridge sampling in some cases (S9B and S9D Fig) [9]. We did not observe this type of deviation for log-LM or RINT-LM (S9B and S9D Fig) [9]. These observations suggest that, in the absence of minor allele homozygotes, MAP estimation and Laplace approximation can lead to inaccuracies in BMS with log-NL.

In our simulation analysis using MAP approximation followed by Laplace approximation (see the main text), out of 8000 feature-SNP pairs (i.e., 1000 instances per data-generating model), 982 were without minor allele homozygote. In scenarios 1, 2, 3, and 4, we respectively observed 1, 2, 2, and 1 instances where the optimization for at least one of the eight models terminated with an estimate of the Hessian matrix that was not positive definite and, thus, the marginal likelihood was not computable. The calibration and ROC curves without these feature-SNP pairs still suggested reasonable overall performances [9].

Given these results, we propose to optimize hyperparameters using MAP estimation followed by Laplace approximation for the computational efficiency and then compute the posterior probability using MCMC and bridge sampling for the accuracy and robustness.

2.2 Optimizing hyperparameters

We optimized the hyperparameters of the effect prior for the simulated and experimental data using MAP estimation followed by Laplace approximation (see **Methods**). For the simulation, we obtained identical optimal values whether we included or excluded feature-SNP pairs lacking minor allele homozygotes (S5 Table). As expected, log-NL recovered the standard deviations of the non-zero coefficients relative to the residual error standard deviation ($(1.50, 2.00, 1.00)^T$) used for generating the data except for the scenario where the data was generated with random effect but BMS was performed without random effect. For the eQTL and caQTL data in hNPCs, we obtained the optimal values including feature-SNP pairs without minor allele homozygotes (S6 Table). We

note that, out of the 98 response eQTLs and 1775 response caQTLs, 15 and 398 respectively lacked minor allele homozygotes.

References

[1] Pejman Mohammadi, Stephane E. Castel, Andrew A. Brown, and Tuuli Lappalainen. Quantifying the regulatory effect size of *cis* -acting genetic variation using allelic fold change. *Genome Research*, 27(11):1872–1884, November 2017.

[2] John Palowitch, Andrey Shabalin, Yi-Hui Zhou, Andrew B. Nobel, and Fred A. Wright. Estimation of cis-eQTL effect sizes using a log of linear model: Effect Sizes of cis-eQTLs Using a Log of Linear Model. *Biometrics*, 74(2):616–625, June 2018.

[3] Nana Matoba, Brandon D Le, Jordan M Valone, Justin M Wolter, Jessica Mory, Dan Liang, Nil Aygün, K Alaine Broadaway, Marielle L Bond, Karen L Mohlke, Mark J Zylka, Michael I Love, and Jason L Stein. Wnt activity reveals context-specific genetic effects on gene regulation in neural progenitors. preprint, Genetics, February 2023.

[4] Stephanie M Gogarten, Tamar Sofer, Han Chen, Chaoyu Yu, Jennifer A Brody, Timothy A Thornton, Kenneth M Rice, and Matthew P Conomos. Genetic association testing using the GENESIS R/Bioconductor package. *Bioinformatics*, 35(24):5346–5348, December 2019.

[5] Quentin F. Gronau, Henrik Singmann, and Eric-Jan Wagenmakers. **bridgesampling** : An *R* Package for Estimating Normalizing Constants. *Journal of Statistical Software*, 92(10), 2020.

[6] Peter D. Hoff. *A First Course in Bayesian Statistical Methods*. Springer Texts in Statistics. Springer New York, New York, NY, 2009.

[7] Jon Wakefield. *Bayesian and Frequentist Regression Methods*. Springer Series in Statistics. Springer New York, New York, NY, 2013.

[8] Jeff Gill. *Bayesian Methods: A Social and Behavioral Sciences Approach*. Chapman & Hall/CRC statistics in the social and behavioral sciences series. CRC Press, Taylor & Francis Group, Boca Raton, third edition edition, 2015.

[9] Yuriko Harigaya, Michael I. Love, and William Valdar. yharigaya/classifygxt-paper: v1.0.0. `https://doi.org/10.5281/zenodo.14827827`, February 2025.
