# Supplementary material for: Probabilistic classification of gene-by-treatment interactions on molecular count phenotypes
Source: PLoS Genet. 2025 Apr 9;21(4):e1011561. doi: 10.1371/journal.pgen.1011561 (PMC12021428; doi:10.1371/journal.pgen.1011561)
Supplement: S1 File — (ZIP) [file pgen.1011561.s026.zip › classifygxt-0.1.0/docs/articles/tensorqtl.html]

Using ClassifyGxT with TensorQTL • classifygxt
 


  


 


Toggle navigation


classifygxt
0.1.0

- Get started
- Reference
- Articles
  - Using ClassifyGxT with TensorQTL
- Changelog

# Using ClassifyGxT with TensorQTL

#### Yuriko Harigaya, Michael Love, William Valdar

#### 2024-11-22

Source: `vignettes/tensorqtl.Rmd`

`tensorqtl.Rmd`

## Introduction

This vignette demonstrates how to format input data for ClassifyGxT
from example input/output data for a widely used program for expression
QTL mapping, TensorQTL. The
procedure described here focuses on data without repeated measurements
per donor (e.g., gene expression data from clinical samples). For
analyzing data with repeated measurements (e.g., molecular phenotypes
from *in-vitro* cell systems), example code can be found at https://bitbucket.org/steinlabunc/wnt-rqtls and https://github.com/yharigaya/classifygxt-paper.

Since the demonstration relies on large external data, the code in
this vignette is not executed. To execute the code, it is necessary to
download example
data from the TensorQTL website and store them locally. Execution
also requires installation of TensorQTL and PLINK2.

## Data preprocessing

We recommend processing molecular count data as illustrated in steps
1-6 of Data
preprocessing prior to running TensorQTL.

## Running TensorQTL

In this section, we briefly illustrate how to run interaction QTL
mapping using TensorQTL prior to running ClassifyGxT.
See the instruction on TensorQTL for
details.

### Interaction QTL mapping in general

We use the following input data for performing interaction QTL
mapping using TensorQTL in a way that is compatible with
ClassifyGxT.

- PLINK2 genotype files (`.pgen`, `.psam`,
  `.pvar`)
- Molecular phenotypes file: A tab-delimited file of processed gene
  expression data (or other molecular phenotypes) with rows and columns
  representing gene (or feature) IDs and sample IDs, respectively.
- Covariates file: A tab-delimited file of covariates with rows and
  columns representing covariate names and sample IDs, respectively. The
  covariates include molecular phenotype PCs (from step
  6) and, optionally, other nuisance factors (see step
  5).
- Interactions file: A tab-delimited file of the independent variable
  of interest (e.g., treatment conditions coded as \({1, 2}\)). The first and second columns
  must contain sample IDs and the treatment condition variable,
  respectively. A header is not needed.

In general, the following code, taken from the TensorQTL instruction,
performs interaction mapping.

```
python3 -m tensorqtl ${plink_prefix_path} ${expression_bed} ${prefix} \
    --covariates ${covariates_file} \
    --interaction ${interactions_file} \
    --best_only \
    --mode cis_nominal
```

The code will generate an output file,
`${prefix}.cis_qtl_top_assoc.txt.gz`. The
`plink_prefix_path` variable must be the path to the PLINK2
genotype files. That is, the paths to the files must be
`${plink_prefix_path}.pgen`,
`${plink_prefix_path}.pvar`, and
`${plink_prefix_path}.psam`. The `expression_bed`,
`covariates_file`, and `interactions_file`
variables must be the paths to the molecular phenotypes, covariates, and
interactions files, respectively. With this particular setting, nominal
\(P\) values smaller than 0.01 will be
included in the output file.

### An example of interaction QTL mapping

For concreteness, we illustrate the procedure using example
datasets available from the TensorQTL website.

- `GEUVADIS.445_samples.GRCh38.20170504.maf01.filtered.nodup.chr18.pgen`
- `GEUVADIS.445_samples.GRCh38.20170504.maf01.filtered.nodup.chr18.psam`
- `GEUVADIS.445_samples.GRCh38.20170504.maf01.filtered.nodup.chr18.pvar`
- `GEUVADIS.445_samples.covariates.txt`
- `GEUVADIS.445_samples.expression.bed.gz`

We assume that the above files are stored in the `input/`
directory under the working directory. We will save intermediate files
in a separate directory, `processed/`. The covariate file
(`GEUVADIS.445_samples.covariates.txt`) contains 26
covariates including the sex variable (coded as 1 and 2). Here, we
attempt to map QTLs with gene-by-sex interactions. For this purpose, we
first create an interaction file, which contains the sample names and
the sex variable. In R, the following code can be used.

```
library(magrittr)

# read in the data
covar.file <- "input/GEUVADIS.445_samples.covariates.txt"
covar <- covar.file %>% read.delim(row.names=1)

# create a data frame
sample <- colnames(covar)
value <- covar[26, ] %>%
    unlist %>%
    unname %>%
    as.integer
int <- data.frame(sample=sample, value=value)

# specify the name of an interactions file
int.file <- "processed/GEUVADIS.445_samples.interactions.txt")

# write the sex variable to an file
write.table(
    int, file=int.file, quote=FALSE, sep="\t",
    row.names=FALSE, col.names=FALSE)
```

We next create a new covariate file by removing the sex variable from
the covariate file.

```
covar2 <- covar[-26, ]

# specify the name of a new covariates file
covar2.file <- "processed/GEUVADIS.445_samples.covariates2.txt"

# write to the covariates to a file
write.table(
    covar2, file=covar2.file, quote=FALSE, sep="\t",
    row.names=TRUE, col.names=TRUE)
```

We then perform interaction QTL mapping by typing the following code
in a command-line application.

```
# specify the input file names
plink_prefix_path="input/GEUVADIS.445_samples.GRCh38.20170504.maf01.filtered.nodup.chr18"
expression_bed="input/GEUVADIS.445_samples.expression.bed.gz"
covariates_file="processed/GEUVADIS.445_samples.covariates2.txt"
interactions_file="processed/GEUVADIS.445_samples.interactions.txt"

# specify an output prefix
prefix="processed/GEUVADIS.445_samples"

python3 -m tensorqtl ${plink_prefix_path} ${expression_bed} ${prefix} \
    --covariates ${covariates_file} \
    --interaction ${interactions_file} \
    --best_only \
    --mode cis_nominal
```

The result will be saved as
`processed/GEUVADIS.445_samples.cis_qtl_top_assoc.txt.gz`.

## Formatting data for ClassifyGxT

To analyze the interaction QTLs using ClassifyGxT, we first convert
the PLINK2 genotype files to a `.traw` file by typing the
following code in a command-line application.

```
out_prefix="processed/GEUVADIS.445_samples.GRCh38.20170504.maf01.filtered.nodup.chr18"
plink2 --pfile ${plink_prefix_path} --export Av --out ${out_prefix}
```


Next, in R, we specify the input file names.

```
qtl.file <- "processed/GEUVADIS.445_samples.chr18.cis_qtl_top_assoc.txt.gz"
geno.file <- "processed/GEUVADIS.445_samples.GRCh38.20170504.maf01.filtered.nodup.chr18.traw"
pheno.file <- "input/GEUVADIS.445_samples.expression.bed.gz"
covar.file <- "processed/GEUVADIS.445_samples.covariates2.txt"
int.file <- "processed/GEUVADIS.445_samples.interactions.txt"
```

We then attach *classifygxt* and obtain a list object using
`format_input()`.

```
library(classifygxt)

input.list <- format_input(
    qtl=qtl.file, geno=geno.file, pheno=pheno.file,
    covar=covar.file, int=int.file)
```

The `input.list` object above is a list of lists, each of
which corresponds to a feature-SNP pair and can be used as input for
`do_bms()`. Depending on the format of the input files, it
may be necessary to specify additional input arguments. See the function
documentation (`?format_input`) for details.

## Session information

```
sessionInfo()
#> R version 4.1.2 (2021-11-01)
#> Platform: x86_64-apple-darwin17.0 (64-bit)
#> Running under: macOS Big Sur 10.16
#> 
#> Matrix products: default
#> BLAS:   /Library/Frameworks/R.framework/Versions/4.1/Resources/lib/libRblas.0.dylib
#> LAPACK: /Library/Frameworks/R.framework/Versions/4.1/Resources/lib/libRlapack.dylib
#> 
#> locale:
#> [1] en_US.UTF-8/en_US.UTF-8/en_US.UTF-8/C/en_US.UTF-8/en_US.UTF-8
#> 
#> attached base packages:
#> [1] stats     graphics  grDevices utils     datasets  methods   base     
#> 
#> loaded via a namespace (and not attached):
#>  [1] rstudioapi_0.13   knitr_1.34        magrittr_2.0.1    R6_2.5.1         
#>  [5] ragg_1.2.5        rlang_1.1.1       fastmap_1.1.1     stringr_1.4.0    
#>  [9] tools_4.1.2       xfun_0.26         cli_3.6.1         jquerylib_0.1.4  
#> [13] htmltools_0.5.5   systemfonts_1.0.4 yaml_2.2.1        digest_0.6.27    
#> [17] lifecycle_1.0.4   pkgdown_2.0.9     textshaping_0.3.6 purrr_1.0.1      
#> [21] sass_0.4.6        htmlwidgets_1.5.4 vctrs_0.6.2       fs_1.5.0         
#> [25] memoise_2.0.1     cachem_1.0.6      evaluate_0.14     rmarkdown_2.11   
#> [29] stringi_1.7.4     compiler_4.1.2    bslib_0.3.0       desc_1.4.3       
#> [33] jsonlite_1.7.2
```

## Contents

Developed by Yuriko Harigaya, Michael Love, William Valdar.

Site built with pkgdown 2.0.9.
