# Supplementary material for: Probabilistic classification of gene-by-treatment interactions on molecular count phenotypes
Source: PLoS Genet. 2025 Apr 9;21(4):e1011561. doi: 10.1371/journal.pgen.1011561 (PMC12021428; doi:10.1371/journal.pgen.1011561)
Supplement: S1 File — (ZIP) [file pgen.1011561.s026.zip › classifygxt-0.1.0/docs/authors.html]

Authors and Citation • classifygxt       

Toggle navigation


classifygxt
0.1.0

- Get started
- Reference
- Articles
  - Using ClassifyGxT with TensorQTL
- Changelog

# Authors and Citation

- **Yuriko Harigaya**. Author, maintainer.
- **Michael Love**. Author.
- **William Valdar**. Author.

# Citation

Source: `DESCRIPTION`

Harigaya Y, Love M, Valdar W (2024).
*classifygxt: ClassifyGxT - classifying gene-by-treatment interactions*.
R package version 0.1.0, https://github.com/yharigaya/classifygxt.

```
@Manual{,
  title = {classifygxt: ClassifyGxT - classifying gene-by-treatment interactions},
  author = {Yuriko Harigaya and Michael Love and William Valdar},
  year = {2024},
  note = {R package version 0.1.0},
  url = {https://github.com/yharigaya/classifygxt},
}
```

Developed by Yuriko Harigaya, Michael Love, William Valdar.

Site built with pkgdown 2.0.9.
