# Supplementary material for: Probabilistic classification of gene-by-treatment interactions on molecular count phenotypes
Source: PLoS Genet. 2025 Apr 9;21(4):e1011561. doi: 10.1371/journal.pgen.1011561 (PMC12021428; doi:10.1371/journal.pgen.1011561)
Supplement: S1 File — (ZIP) [file pgen.1011561.s026.zip › classifygxt-0.1.0/docs/reference/classifygxt-package.html]

The 'classifygxt' package. — classifygxt-package • classifygxt       

Toggle navigation


classifygxt
0.1.0

- Get started
- Reference
- Articles
  - Using ClassifyGxT with TensorQTL
- Changelog

# The 'classifygxt' package.

Source: `R/classifygxt-package.R`

`classifygxt-package.Rd`

A DESCRIPTION OF THE PACKAGE

## References

Stan Development Team (2022). RStan: the R interface to Stan. R package version 2.21.5. https://mc-stan.org

## Contents

Developed by Yuriko Harigaya, Michael Love, William Valdar.

Site built with pkgdown 2.0.9.
