# Supplementary material for: Probabilistic classification of gene-by-treatment interactions on molecular count phenotypes
Source: PLoS Genet. 2025 Apr 9;21(4):e1011561. doi: 10.1371/journal.pgen.1011561 (PMC12021428; doi:10.1371/journal.pgen.1011561)
Supplement: S1 File — (ZIP) [file pgen.1011561.s026.zip › classifygxt-0.1.0/docs/reference/format_gp.html]

Prepare data for a genotype-phenotype plot — format\_gp • classifygxt       

Toggle navigation


classifygxt
0.1.0

- Get started
- Reference
- Articles
  - Using ClassifyGxT with TensorQTL
- Changelog

# Prepare data for a genotype-phenotype plot

Source: `R/plot.R`

`format_gp.Rd`

This is a function to prepare data for visualization using
`make_gp_plot`.

```
format_gp(data, fit, seed = 1)
```

## Arguments

data
:   A list containing phenotype, genotype, treatment, and
    subject, which must be named "y", "g", "t", and "subject ",
    respectively.

fit
:   A list obtained from the `do_bms`.

seed
:   A seed for RNG.

## Value

A list of data frames.

## Contents

Developed by Yuriko Harigaya, Michael Love, William Valdar.

Site built with pkgdown 2.0.9.
