# Supplementary material for: Probabilistic classification of gene-by-treatment interactions on molecular count phenotypes
Source: PLoS Genet. 2025 Apr 9;21(4):e1011561. doi: 10.1371/journal.pgen.1011561 (PMC12021428; doi:10.1371/journal.pgen.1011561)
Supplement: S1 File — (ZIP) [file pgen.1011561.s026.zip › classifygxt-0.1.0/docs/reference/format_input.html]

Format input — format\_input • classifygxt       

Toggle navigation


classifygxt
0.1.0

- Get started
- Reference
- Articles
  - Using ClassifyGxT with TensorQTL
- Changelog

# Format input

Source: `R/utils.R`

`format_input.Rd`

This function takes input and output files of
TensorQTL and
returns a list object that can be used as input for
`do_bms`. It currenly only handles completely unpaired data,
where there is no repeated measurement per donor (subject). This
includes typical molecular data from clinical trials.

```
format_input(
  qtl,
  pheno,
  geno,
  covar,
  int,
  pheno.col = "phenotype_id",
  variant.col = "variant_id",
  feat.col = "gene_id",
  snp.col = "SNP",
  pheno.rm = 1:4,
  geno.rm = 1:6
)
```

## Arguments

qtl
:   A character string specfying the name of the output file
    from interacton QTL mapping from TensorQTL.

pheno
:   A character string specifynig the name of the file
    containing molecular phenotes. This must be the same as the
    input file used for TensorQTL.

geno
:   A character string specifying the name of the genotype
    file in PLINK2
    `.traw` format.

covar
:   A character string specifyng the name of the file
    containing covariates. This must be the same as the input file
    used for TensorQTL.

int
:   A character string specifying the name of the file
    containing the condition variable of interest, coded as 1,
    2. This must be the same as the input file used for TensorQTL.

pheno.col
:   A character string specifying the name of the
    column of `qtl` containing the feature IDs.

variant.col
:   A character string specifying the name of the
    column of `qtl` containing the feature IDs.

feat.col
:   A character string specifying the name of the
    column of `pheno` containing the feature IDs.

snp.col
:   A character string specifying the name of the
    column of `geno` containing the SNP IDs.

pheno.rm
:   A vector of integers corresponding to the column
    numbers for non-phenotype entries in `pheno`.

geno.rm
:   A vector of integers corresponding to the column
    numbers for non-phenotype entries in `geno`.

## Value

A list of lists containing:

- `y` - A vector of phenotypes.
- `g` - A vector of genotypes.
- `t` - A vector of treatment indicators.
- `subject` - A vector of subject.
- `feat.id` - A character string spcifyng the feature ID.
- `snp.id` - A character string specifying the SNP ID.

## Contents

Developed by Yuriko Harigaya, Michael Love, William Valdar.

Site built with pkgdown 2.0.9.
