# Supplementary material for: Probabilistic classification of gene-by-treatment interactions on molecular count phenotypes
Source: PLoS Genet. 2025 Apr 9;21(4):e1011561. doi: 10.1371/journal.pgen.1011561 (PMC12021428; doi:10.1371/journal.pgen.1011561)
Supplement: S1 File — (ZIP) [file pgen.1011561.s026.zip › classifygxt-0.1.0/docs/reference/format_pp.html]

Prepare data for a barplot of posterior probability — format\_pp • classifygxt       

Toggle navigation


classifygxt
0.1.0

- Get started
- Reference
- Articles
  - Using ClassifyGxT with TensorQTL
- Changelog

# Prepare data for a barplot of posterior probability

Source: `R/plot.R`

`format_pp.Rd`

This is a function to prepare data for visualization using
`make_pp_plot`.

```
format_pp(fit, co = FALSE)
```

## Arguments

fit
:   A list obtained from the `do_bms`.

co
:   A logical varialbe as to whether to visualize the
    probability of crossover interaction. If this is set to
    `TRUE`, `summary` must be set to `FALSE` when
    running `do_bms`.

## Value

A data frame.

## Contents

Developed by Yuriko Harigaya, Michael Love, William Valdar.

Site built with pkgdown 2.0.9.
