# Supplementary material for: Probabilistic classification of gene-by-treatment interactions on molecular count phenotypes
Source: PLoS Genet. 2025 Apr 9;21(4):e1011561. doi: 10.1371/journal.pgen.1011561 (PMC12021428; doi:10.1371/journal.pgen.1011561)
Supplement: S1 File — (ZIP) [file pgen.1011561.s026.zip › classifygxt-0.1.0/docs/reference/get_est.html]

Extract parameter estimates — get\_est • classifygxt       

Toggle navigation


classifygxt
0.1.0

- Get started
- Reference
- Articles
  - Using ClassifyGxT with TensorQTL
- Changelog

# Extract parameter estimates

Source: `R/core.R`

`get_est.Rd`

This is a function to extract parameter estimates from
the output from `do_bms`. If `model` is not
specified, estimates for the MAP model is returned.

```
get_est(fit, model = NULL)
```

## Arguments

fit
:   A list obtained from the `do_bms`.

model
:   A character string or integer specifying the model.

## Value

A named vector of parameter estimates.

## Contents

Developed by Yuriko Harigaya, Michael Love, William Valdar.

Site built with pkgdown 2.0.9.
