# Supplementary material for: Probabilistic classification of gene-by-treatment interactions on molecular count phenotypes
Source: PLoS Genet. 2025 Apr 9;21(4):e1011561. doi: 10.1371/journal.pgen.1011561 (PMC12021428; doi:10.1371/journal.pgen.1011561)
Supplement: S1 File — (ZIP) [file pgen.1011561.s026.zip › classifygxt-0.1.0/docs/reference/get_pp.html]

Extract posterior probability — get\_pp • classifygxt       

Toggle navigation


classifygxt
0.1.0

- Get started
- Reference
- Articles
  - Using ClassifyGxT with TensorQTL
- Changelog

# Extract posterior probability

Source: `R/core.R`

`get_pp.Rd`

This is a function to extract posterior probability from
the output from `do_bms`. It optionally
aggregates the model categories.

```
get_pp(fit, aggregate = "none")
```

## Arguments

fit
:   A list obtained from the `do_bms`.

aggregate
:   An optional character string specifying
    whether and how to aggregate the model categories.
    This must be one of "none", "genotype", and "treatment".

## Value

a named vector of posterior probability

## Contents

Developed by Yuriko Harigaya, Michael Love, William Valdar.

Site built with pkgdown 2.0.9.
