# Supplementary material for: Probabilistic classification of gene-by-treatment interactions on molecular count phenotypes
Source: PLoS Genet. 2025 Apr 9;21(4):e1011561. doi: 10.1371/journal.pgen.1011561 (PMC12021428; doi:10.1371/journal.pgen.1011561)
Supplement: S1 File — (ZIP) [file pgen.1011561.s026.zip › classifygxt-0.1.0/docs/reference/get_sign_names.html]

Get the names of the 27 models — get\_sign\_names • classifygxt       

Toggle navigation


classifygxt
0.1.0

- Get started
- Reference
- Articles
  - Using ClassifyGxT with TensorQTL
- Changelog

# Get the names of the 27 models

Source: `R/utils.R`

`get_sign_names.Rd`

This function returns an ordered vector of character strings
corresponding to the names of the 27 models accounting for the
sign of effect sizes.

```
get_sign_names()
```

## Value

A vector containing the eight model names.

## Contents

Developed by Yuriko Harigaya, Michael Love, William Valdar.

Site built with pkgdown 2.0.9.
