# Supplementary material for: Probabilistic classification of gene-by-treatment interactions on molecular count phenotypes
Source: PLoS Genet. 2025 Apr 9;21(4):e1011561. doi: 10.1371/journal.pgen.1011561 (PMC12021428; doi:10.1371/journal.pgen.1011561)
Supplement: S1 File — (ZIP) [file pgen.1011561.s026.zip › classifygxt-0.1.0/docs/reference/make_gp_plot.html]

Make a genotype-phenotype plot with model fit — make\_gp\_plot • classifygxt       

Toggle navigation


classifygxt
0.1.0

- Get started
- Reference
- Articles
  - Using ClassifyGxT with TensorQTL
- Changelog

# Make a genotype-phenotype plot with model fit

Source: `R/plot.R`

`make_gp_plot.Rd`

This function generates a genotype-phenotype plot with regression
lines in the control and treated conditions based on the MAP model.

```
make_gp_plot(gp, title = NULL, seed = 1)
```

## Arguments

gp
:   A list object obtained from the `format_gp`
    function.

title
:   A character string specifying a title.

seed
:   A seed for RNG.

## Value

A `ggplot2` object

## Contents

Developed by Yuriko Harigaya, Michael Love, William Valdar.

Site built with pkgdown 2.0.9.
