# Supplementary material for: Probabilistic classification of gene-by-treatment interactions on molecular count phenotypes
Source: PLoS Genet. 2025 Apr 9;21(4):e1011561. doi: 10.1371/journal.pgen.1011561 (PMC12021428; doi:10.1371/journal.pgen.1011561)
Supplement: S1 File — (ZIP) [file pgen.1011561.s026.zip › classifygxt-0.1.0/docs/reference/make_heatmap.html]

Make a heatmap of posterior probability — make\_heatmap • classifygxt       

Toggle navigation


classifygxt
0.1.0

- Get started
- Reference
- Articles
  - Using ClassifyGxT with TensorQTL
- Changelog

# Make a heatmap of posterior probability

Source: `R/plot.R`

`make_heatmap.Rd`

This is a function to make a heatmap of posterior probability for a
set of feature-SNP pairs.

```
make_heatmap(input)
```

## Arguments

input
:   A matrix containing posterior probability. Rows and
    columns must represent feature-SNP pairs and model categories,
    respectively.

## Value

A `ggplot2` object.

## Contents

Developed by Yuriko Harigaya, Michael Love, William Valdar.

Site built with pkgdown 2.0.9.
